# Defying strain in the synthesis of an electroactive bilayer helicene†
†Electronic supplementary information (ESI) available. CCDC 1864289 and 1864290. For ESI and crystallographic data in CIF or other electronic format see DOI: 10.1039/c8sc04216k


**DOI:** 10.1039/c8sc04216k

**Published:** 2018-11-20

**Authors:** Margarita Milton, Nathaniel J. Schuster, Daniel W. Paley, Raúl Hernández Sánchez, Fay Ng, Michael L. Steigerwald, Colin Nuckolls

**Affiliations:** a Department of Chemistry , Columbia University , New York , New York 10027 , USA . Email: njs2154@columbia.edu ; Email: mls2064@columbia.edu ; Email: cn37@columbia.edu; b Columbia Nano Initiative , Columbia University , New York , New York 10027 , USA; c Department of Chemistry , University of Pittsburgh , Pittsburgh , Pennsylvania 15260 , USA

## Abstract

We report the synthesis of a bilayer chiral nanographene incorporating a [7]helicene scaffold and two perylene-diimide (PDI) subunits. Twofold visible-light-induced oxidative cyclization of a phenanthrene framework selects for the desired PDI-helicene, despite the immense strain that distinguishes this helicene from two other accessible isomers. This strain arises from the extensive intramolecular overlap of the PDI subunits, which precludes racemization, even at elevated temperatures. Relative to a smaller homologue, this PDI-helicene exhibits amplified electronic circular dichroism. It also readily and reversibly accepts four electrons electrochemically. Modifications to the core phenanthrene subunit change the fluorescence and electrochemistry of the PDI-helicene without significantly impacting its electronic circular dichroism or UV-visible absorbance.

## Abstract 2

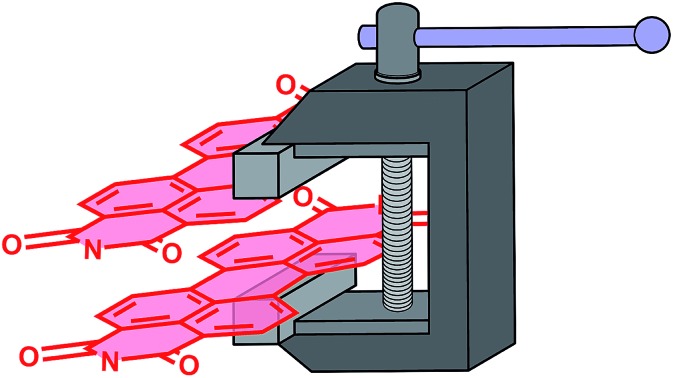
Visible-light-induced oxidative cyclization of a phenanthrene framework overcomes immense strain to yield a bilayer perylene-diimide helicene.

## Introduction

**Fig. 1:**
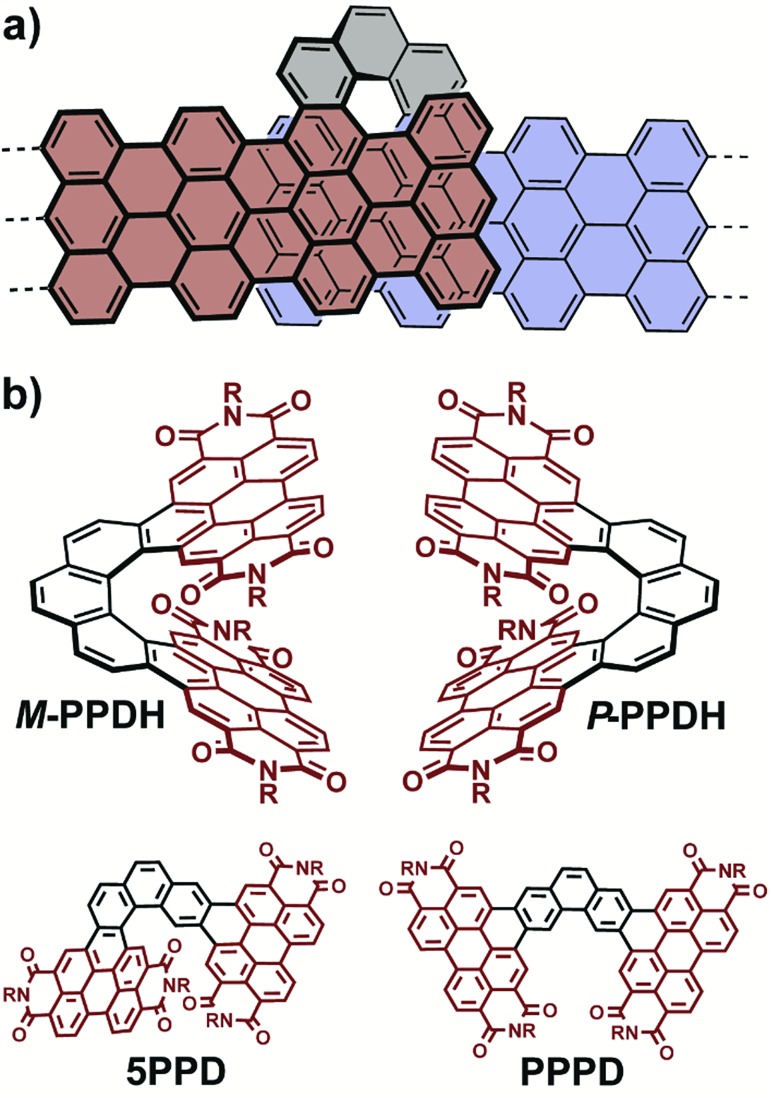
(a) The fusion of two armchair nanographenes with phenanthrene provides a π-extended bilayer helicene. (b) Fusion with two PDI subunits *via* oxidative photocyclization at elevated temperatures overwhelmingly favors the formation of **PPDH** over its isomers, **5PPD** and **PPPD**.

This study concerns the regioselective synthesis and characterization of the newest and most highly strained perylene-3,4,9,10-tetracarboxylic-diimide (PDI) helicene. In general, interactions between π-surfaces account for a variety of interesting electronic, optical, and magnetic phenomena. For instance, π-to-π overlap promotes charge transport within organic semiconductors,^1^,^2^ a key consideration in the design of organic electronic and optoelectronic devices.^3^,^4^ π-Extension of the termini of helicenes – helices of fused aromatic subunits surrounding a nonintersecting stereogenic axis – provides chiral nanographenes with considerable intramolecular π-to-π overlap (Fig. 1a). Such chiral nanographenes may exhibit very large circular dichroism,^5^–^7^ circularly polarized luminescence,^8^,^9^ non-linear optical properties,^10^,^11^ and chiral-induced spin selectivity.^12^

The synthesis of helicenes, especially those with extensively eclipsed π-surfaces, hinges on the strategic accumulation of strain.^13^–^15^ Some synthetic complications can be avoided by attaching bulky polyaromatics after forming the helicene core.^16^ For other approaches, the possibility of unwanted cyclizations and attenuated reactivity must be considered.^17^–^24^ Clear synthetic guidelines exist for the preparation of helicenes *via* the oxidative photocyclization of stilbene-like scaffolds incorporating phenylene and naphthylene subunits.^25^ However, ensuring the exclusive formation of helicenes from the oxidative photocyclization of phenanthrene-based substrates remains difficult,^26^,^27^ especially when both twisted and planar (or nearly planar) polyaromatic products can be formed.^28^

Here we disclose the synthesis of a π-extended [7]helicene *via* the regioselective visible-light-induced oxidative fusion of phenanthrene with two PDI subunits. This phenanthryl-linked PDI-dimer helicene (**PPDH**, Fig. 1b) belongs to an emerging class of chiral, shape-persistent, PDI-based materials prepared by intramolecular oxidative photocyclizations.^29^–^31^ Extensive intramolecular overlap of the π-surface distinguishes **PPDH** from its PDI-dimer helicene predecessors and the vast majority of π-extended helicenes.^32^–^41^ The strain attendant on this overlap disfavors the exclusive synthesis of **PPDH** near room temperature: the undesired [5]helicene isomer (**5PPD**, Fig. 1b) also forms. Simply raising the reaction temperature gives near-exclusive production of **PPDH**. This regioselectivity belies the disparity in strain between **PPDH**, **5PPD**, and their planar isomer (**PPPD**, Fig. 1b). Single-crystal X-ray diffraction (SCXRD) of **PPDH** confirmed extensive intramolecular overlap of the π-surface, which contorts to mitigate intramolecular PDI-to-PDI contact. **PPDH** exhibits large electronic circular dichroism (ECD) in the visible range, especially relative to a smaller helical homologue, and readily accepts up to four electrons electrochemically. Finally, the fluorescence and electrochemistry of **PPDH** can be tuned by changing the substituents on its phenanthrene core.

## Results and discussion

**Scheme 1:**
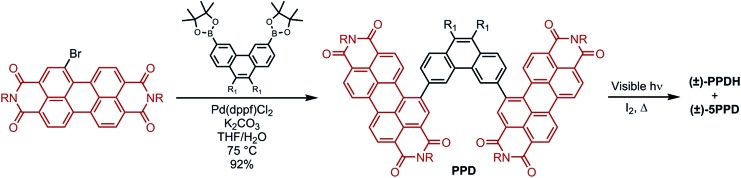
Two-step synthesis of **PPDH** from brominated PDI [R = CH(C_5_H_11_)_2_].

Scheme 1 depicts our synthesis of **PPDH**. Intramolecular oxidative photocyclizations have been used to synthesize several helicenes from aryl-linked PDI-oligomers.^42^,^43^ Acenes, in particular, fuse preferentially at the more sterically hindered *peri*-position under these reaction conditions,^29^–^31^,^44^ whereas oxidative photocyclizations onto 3,6-disubstituted phenanthrene (such as **PPD** in Scheme 1) may occur at any combination of the adjacent positions. We found that visible-light-induced oxidative cyclization of **PPD** at 110 °C formed **PPDH** almost exclusively (entry 3, Table 1). Repeating the oxidative photocyclization of **PPD** at 70 °C lowered the regioselectivity for **PPDH**: a 9 : 1 mixture of **PPDH** and **5PPD**, respectively, resulted. A reaction temperature of 30 °C further decreased this ratio of **PPDH**-to-**5PPD** to 7 : 3. We never detected **PPPD**, no matter the reaction temperature. This absence of sterically unencumbered **PPPD** and the modest yields of **5PPD** underscore a strong electronic preference for **PPDH** under these reaction conditions. Substituting two pentoxy substituents onto the phenanthrene linker does not alter this preference: these pentoxy substituents slowed the oxidative photocyclization of **PPD** (entry 4, Table 1), but did not dramatically alter the yield.

**Table 1:** Optimization of the twofold oxidative photocyclization of **PPD**^*a*^

Entry	R_1_	*T* (°C)	Solvent	Time (h)	**PPDH** : **5PPD**^*b*^	Isolated yield of **PPDH** (%)
1	H	30	PhH	24	70 : 30	63
2	H	70	PhH	24	91 : 9	83
3	H	110	PhCl	24	>95 : <5	91
4	OC_5_H_11_	70	PhH	76	—^*c*^	88^*d*^

^*a*^The solutions of **PPD** (0.2 mM) and iodine (1.3 mM) were irradiated with two 55 W compact fluorescent lamps. Only a silica plug was necessary to remove **5PPD** from **PPDH**. See the ESI for additional details.

^*b*^Determined by ^1^H NMR of the product mixture.

^*c*^ **5PPD** was not observed, perhaps due to decomposition.

^*d*^Reaction did not go to completion; the remainder consisted of mono-cyclized intermediate and decomposition byproduct.

The strain energies of **5PPD**, **PPDH**, and **PPPD** have no bearing on the regioselectivity observed for the twofold oxidative photocyclization of **PPD**. The DFT-optimized (B3LYP/6-31G**) structures of **5PPD** and **PPDH** are more strained (by 11 and 21 kcal mol^–1^, respectively) than **PPPD** (see Section VII of the ESI† for the calculation details). Despite this strain, **PPDH** does not rearrange or decompose otherwise under ambient conditions, enabling the resolution of its left- (*M*) and right-handed (*P*) helices *via* chiral HPLC (Fig. S1†). The obvious rigidity of this PDI-helicene precludes inversion: optically pure **PPDH** heated in diphenyl ether at 250 °C under air for one hour showed neither racemization nor decomposition by HPLC (Fig. S2†). This stability contrasts with the relative lability of [7]helicene alone, which racemizes completely in solution in just over 3 h at ∼257 °C.^45^

**Fig. 2:**
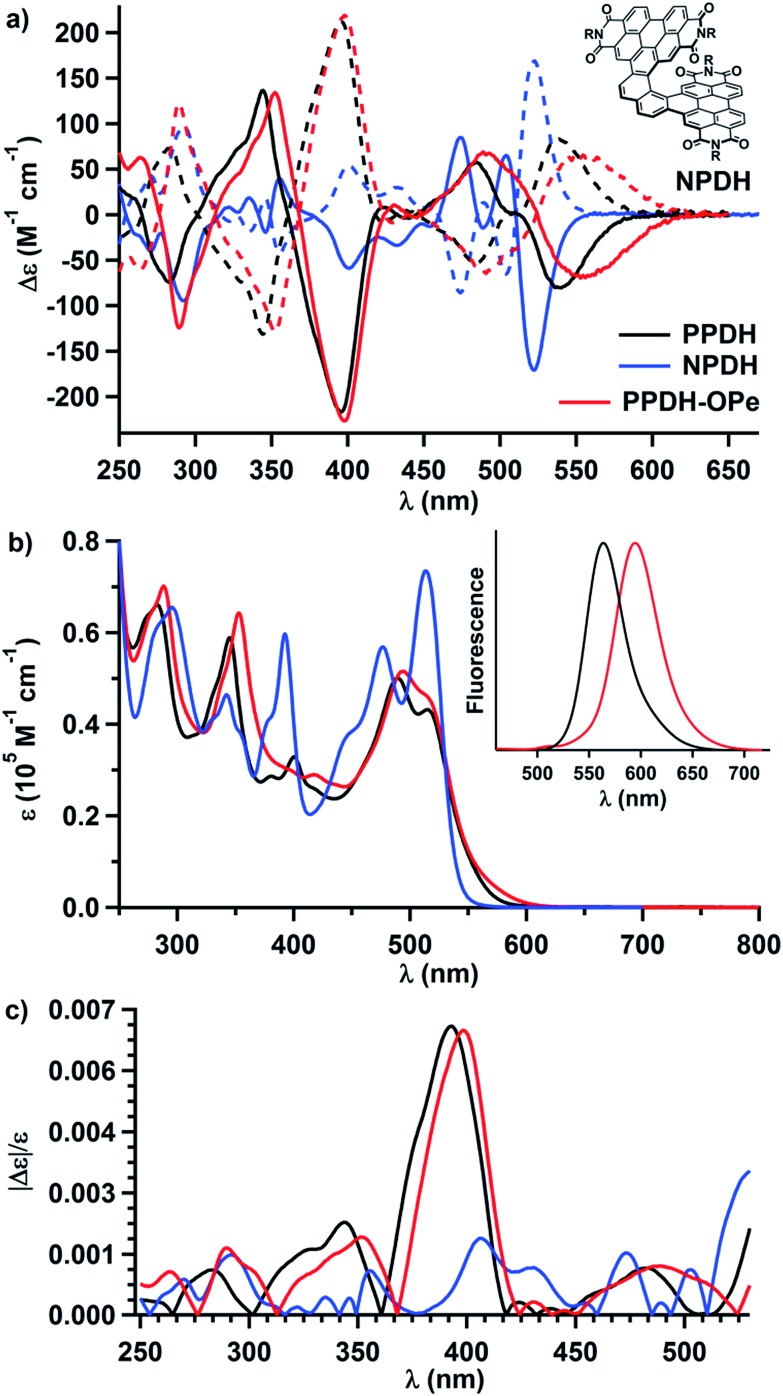
(a) ECD, (b) UV-visible absorbance, and (c) *g*-factors of **PPDH**, **PPDH-OPe**, and **NPDH** in THF (10 μM, 1 cm path length). (Inset of b) Normalized fluorescence intensities of **PPDH** and **PPDH-OPe** in cyclohexane (3 μM, *λ*_ex_ = 410 nm; *Φ*_f_ = 41% and 15% for **PPDH** and **PPDH-OPe**, respectively). For **NPDH**, R = CH(C_5_H_11_)_2_.

Fig. 2a displays the ECD spectra of the resolved enantiomers of **PPDH** and its bis(pentoxy) analogue, **PPDH-OPe** (entry 4, Table 1). They exhibit many Cotton effects across the UV-visible range. The largest transitions of **PPDH** manifest as a bisignate pair centered at ∼360 nm: |Δ*ε*| of 134 M^–1^ cm^–1^ at 344 nm and 214 M^–1^ cm^–1^ at 396 nm. Naphthyl-linked PDI-dimer helicene (**NPDH**; see the inset of Fig. 2a) – the [6]helicene homologue of **PPDH** – shares this feature, albeit its corresponding Cotton effects are diminished: |Δ*ε*| of 41 M^–1^ cm^–1^ at 355 nm and 56 M^–1^ cm^–1^ at 401 nm (Fig. 2a). This strong enhancement in ECD from **NPDH** to **PPDH** deviates from the trend observed for the most intense bisignate pair shared by the carbohelicenes. Specifically, lengthening the carbohelicenes (*e.g.*, [6]helicene to [7]helicene) has little effect on the intensity of the longer wavelength Cotton effect of this pair, which plateaus at ∼260 M^–1^ cm^–1^.^46^

Chiral oligomers often exhibit ECD whose intensity scales linearly (or nearly linearly) with UV-visible absorbance;^23^,^47^ however, the large increase in ECD from **NPDH** to **PPDH** in the 340–410 nm regime cannot be attributed to an increase in absorbance of unpolarized light. Relative to the Cotton effects of **NPDH** at 355 and 401 nm, the Cotton effects of **PPDH** at 344 and 396 nm show a greater than threefold increase in Δ*ε*. The same increases are not observed between the UV-visible absorbance spectra of these PDI-helicenes (Fig. 2b). In fact, the absorbance of **NPDH** and **PPDH** essentially match at 401 and 396 nm, respectively, resulting in a significant disparity in *g*-factor (|Δ*ε*|/*ε*) at these wavelengths: 1.5 × 10^–3^ for **NPDH** and 6.8 × 10^–3^ for **PPDH** (Fig. 2c).

In addition to amplifying the ECD of **PPDH** compared to **NPDH**, the phenanthrene bridge also tunes photoluminescence. The pentoxy chains of **PPDH-OPe** lower the fluorescence quantum yield (*Φ*_f_) to 15%, compared to 41% for unsubstituted **PPDH**. We attribute this decrease to intramolecular donor–acceptor charge transfer following excitation of the PDI-helicene. Excited-state charge transfer also accounts for fluorescence quenching in PDI-based compounds with π-donor substituents.^48^–^50^ On the other hand, the UV-visible and ECD spectra of **PPDH** and **PPDH-OPe** are nearly identical.

**Fig. 3:**
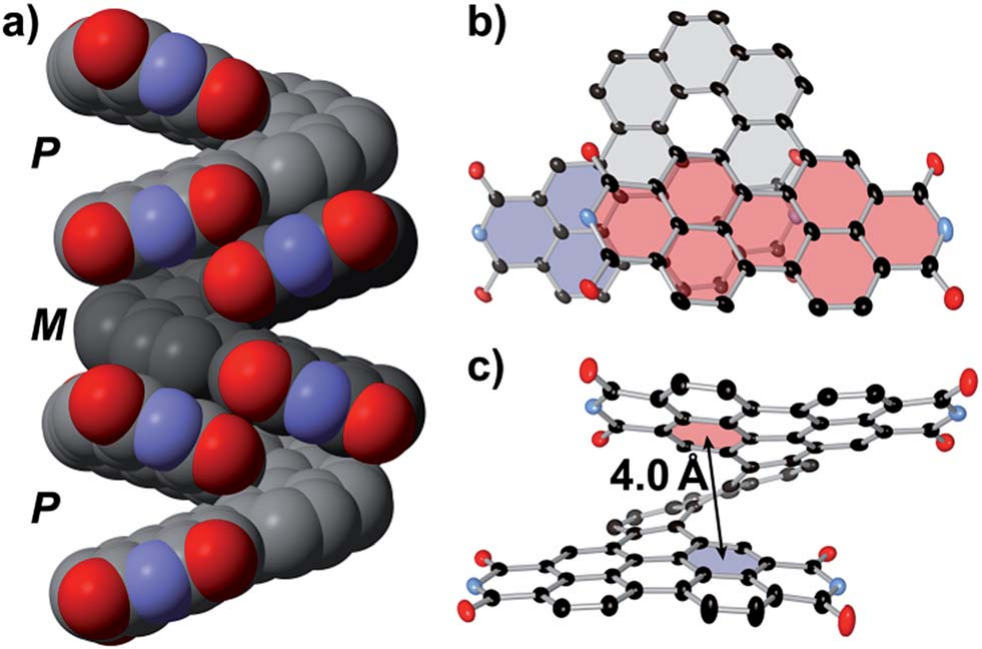
Structure of **PPDH** from SCXRD. Free solvent, the CH(C_5_H_11_)_2_ chains, and hydrogen atoms have been hidden to provide an unobstructed view of the aryl surface. Thermal ellipsoids are set at 30% probability. (a) The racemate assembles as heterochiral columns in the solid state. Extensive intramolecular overlap between the PDI subunits (b) distorts their perylene cores into shallow bowls (c).

SCXRD revealed that the racemate of **PPDH** assembles into columns of alternating *M*- and *P*-helices (Fig. 3a and S3†). The intermolecular junction in these columns consists of 24 pairs of eclipsing π-bonded carbon atoms (see Fig. S4† for clarification), four of which approach to within 3.4 Å (*i.e.*, twice the van der Waals radius of the carbon atom). This heterochiral columnar packing arrangement resembles that observed previously for a π-helix-of-PDI-helicenes^31^ but differs from the crystal structures of other helical bilayer nanographenes.^16^,^51^ For these latter species, C–H···π interactions predominate, stymying intermolecular π-to-π overlap.^52^

SCXRD also revealed extensive intramolecular overlap in **PPDH** (Fig. 3b); this contorts the π-surface. Each PDI subunit warps along its imide-to-imide axis, resulting in bend angles of 9° and 11° between the naphthalene fragments in each perylene (Fig. S5†). For reference, this angle in crystalline PDI-helicenes incorporating [5] and [6]helicene scaffolds measures 3° and 6°, respectively.^31^,^43^ The [7]helicene scaffold in **PPDH** also splays farther from planarity than [7]helicene alone: the dihedral angle defined by the terminal rings of the [7]helicene within **PPDH** measures 40°, whereas the same angle in crystalline [7]helicene measures 32°.^53^,^54^ Moreover, the centroid-to-centroid distance between these same rings in **PPDH** equals 4.0 Å (Fig. 3c); the difference is 3.8 Å in carbo[7]helicene. The resultant gap between the PDI-subunits can host guests: for instance, part of a molecule of α,α,α-trifluorotoluene fills this π-cavity in the crystal structure of **PPDH-OPe** (Fig. S6†).

**Fig. 4:**
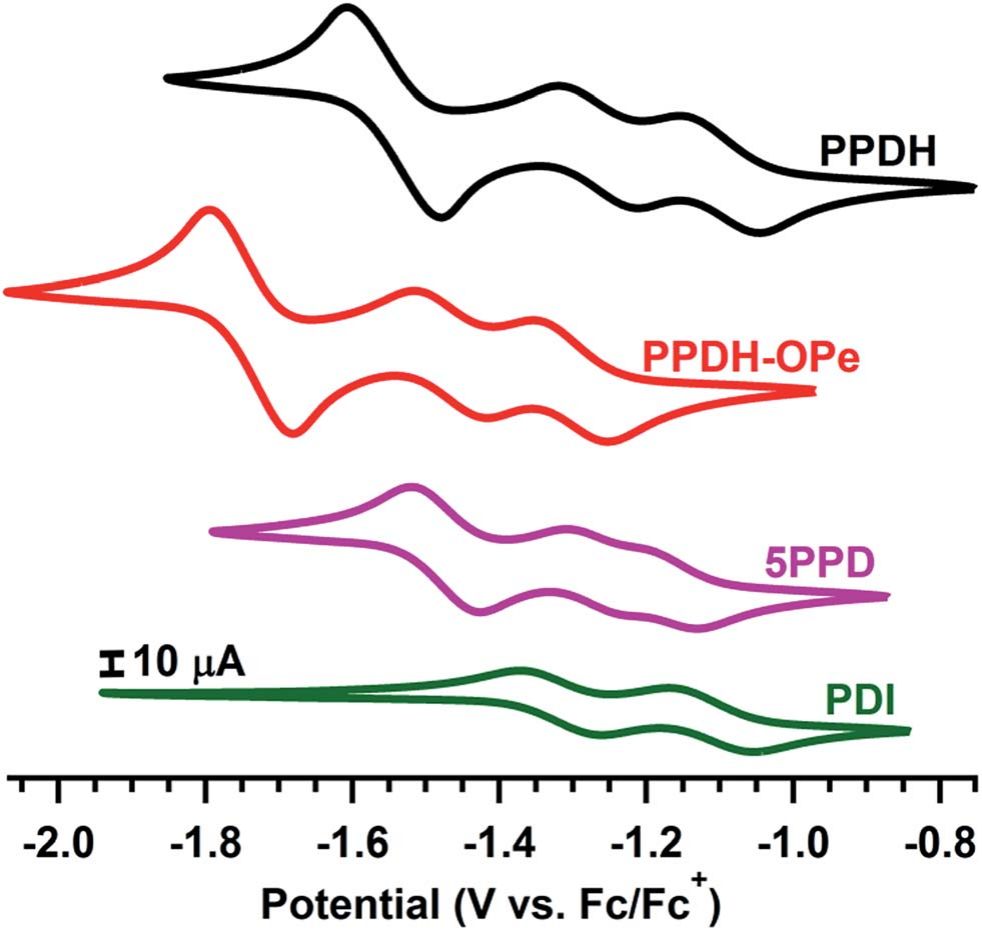
Cyclic voltammograms of **PPDH**, **PPDH-OPe**, **5PPD**, and monomeric PDI (1 mM, 50 mV s^–1^ scan rate) in Ar-sparged DCM with 0.1 M [Bu_4_N][PF_6_] as the supporting electrolyte. For all species, R = CH(C_5_H_11_)_2_.

Cyclic voltammetry (CV) of **PPDH** in dichloromethane revealed three well-resolved reduction events (Fig. 4), all of which are chemically reversible. The two initial events (–1.10 and –1.27 V *vs.* Fc/Fc^+^) are one electron processes, whereas the greater intensity of the third (–1.54 *vs.* Fc/Fc^+^) affirms the near-simultaneous addition of the third and fourth electrons; thus, **PPDH** accepts one electron per imide group. Similar three-event CV profiles have been observed for non-conjugated PDI-dimers with extensive intramolecular π-to-π overlap;^55^–^57^ therefore, one might assume the fused phenanthrene linker in **PPDH** has a limited impact on the electrochemistry of the molecule. In fact, the bis(pentoxy)phenanthrene in **PPDH-OPe** cathodically shifts each reduction event by 200 mV (–1.30, –1.47, and –1.74 V *vs.* Fc/Fc^+^). This LUMO elevation exceeds the –180 mV shift observed upon connecting two dodecoxy substituents directly to the bay regions of monomeric PDI.^58^ As such, the phenanthrene linker can be modified to significantly tune the onset of reduction in **PPDH**, without altering the overall electrochemical profile.

## Conclusions

Visible-light-induced oxidative cyclization of phenanthryl-linked PDI-dimer **PPD** yields the bilayer [7]helicene **PPDH**. Increasing the reaction temperature favors the near-exclusive formation of **PPDH** over its less strained isomers, **5PPD** and **PPPD**. Intramolecular overlap of the π-surface of **PPDH** precludes racemization, even in solution at 250 °C, and results in a voltammetric profile consistent with those of dimeric PDI molecules with similarly eclipsed surfaces. Substitutions onto the phenanthrene linker change the electrochemistry and some of the spectroscopic properties of **PPDH**. For instance, bis(pentoxy)phenanthrene quenches fluorescence and cathodically shifts the onset of reduction; however, it negligibly alters the UV-visible absorbance and ECD of **PPDH**. Racemic **PPDH** self-assembles into heterochiral columns in the solid state. The extensive intermolecular π-to-π overlap within these columns could facilitate charge transport in organic electronic devices. Retention of the same crystalline morphology for optically pure **PPDH** would enable the preparation of transistors that respond preferentially to circularly polarized light,^59^ as well as potentially enhance chiral-induced spin selectivity.^60^

## Conflicts of interest

There are no conflicts to declare.

## Supplementary Material

Supplementary information

Crystal structure data

## References

[cit1] Anthony J. E. (2006). Chem. Rev..

[cit2] Coropceanu V., Cornil J., da Silva Filho D. A., Olivier Y., Silbey R., Brédas J.-L. (2007). Chem. Rev..

[cit3] Wang C., Dong H., Hu W., Liu Y., Zhu D. (2012). Chem. Rev..

[cit4] Ostroverkhova O. (2016). Chem. Rev..

[cit5] Berova N., Di Bari L., Pescitelli G. (2007). Chem. Soc. Rev..

[cit6] Pescitelli G., Di Bari L., Berova N. (2011). Chem. Soc. Rev..

[cit7] Pescitelli G., Di Bari L., Berova N. (2014). Chem. Soc. Rev..

[cit8] Kumar J., Nakashima T., Kawai T. (2015). J. Phys. Chem. Lett..

[cit9] Sánchez-Carnerero E. M., Agarrabeitia A. R., Moreno F., Maroto B. L., Muller G., Ortiz M. J., de la Moya S. (2015). Chem.–Eur. J..

[cit10] Gu B., Zhao C., Baev A., Yong K.-T., Wen S., Prasad P. N. (2016). Adv. Opt. Photonics.

[cit11] Dini D., Calvete M. J. F., Hanack M. (2016). Chem. Rev..

[cit12] Naaman R., Waldeck D. H. (2012). J. Phys. Chem. Lett..

[cit13] Laarhoven W. H., Prinsen W. J. C. (1984). Top. Curr. Chem..

[cit14] Shen Y., Chen C.-F. (2012). Chem. Rev..

[cit15] Gingras M. (2013). Chem. Soc. Rev..

[cit16] Evans P. J., Ouyang J., Favereau L., Crassous J., Fernández I., Perles J., Martín N. (2018). Angew. Chem., Int. Ed..

[cit17] Scholz M., Mühlstädt M., Dietz F. (1967). Tetrahedron Lett..

[cit18] Martin R. H., Flammang-Barbieux M., Cosyn J. P., Gelbcke M. (1968). Tetrahedron Lett..

[cit19] Martin R. H., Marchant M.-J., Baes M. (1971). Helv. Chim. Acta.

[cit20] Mallory F. B., Mallory C. W. (1983). J. Org. Chem..

[cit21] Sudhakar A., Katz T. J. (1986). Tetrahedron Lett..

[cit22] Murguly E., McDonald R., Branda N. R. (2000). Org. Lett..

[cit23] Roose J., Achermann S., Dumele O., Diederich F. (2013). Eur. J. Org. Chem..

[cit24] Nakakuki Y., Hirose T., Sotome H., Miyasaka H., Matsuda K. (2018). J. Am. Chem. Soc..

[cit25] Mori K., Murase T., Fujita M. (2015). Angew. Chem., Int. Ed..

[cit26] El Abed R., Ben Hassine B., Genêt J.-P., Gorsane M., Marinetti A. (2004). Eur. J. Org. Chem..

[cit27] Kogiso T., Yamamoto K., Suemune H., Usui K. (2012). Org. Biomol. Chem..

[cit28] Laarhoven W. H., Cuppen T. H. J. H. M., Nivard R. J. F. (1970). Tetrahedron.

[cit29] Schuster N. J., Paley D. W., Jockusch S., Ng F., Steigerwald M. L., Nuckolls C. (2016). Angew. Chem., Int. Ed..

[cit30] Khokhlov K., Schuster N. J., Ng F., Nuckolls C. (2018). Org. Lett..

[cit31] Schuster N. J., Hernández Sánchez R., Bukharina D., Kotov N. A., Berova N., Ng F., Steigerwald M. L., Nuckolls C. (2018). J. Am. Chem. Soc..

[cit32] Lütke Eversloh C., Liu Z., Müller B., Stangl M., Li C., Müllen K. (2011). Org. Lett..

[cit33] Xiao S., Kang S. J., Wu Y., Ahn S., Kim J. B., Loo Y.-L., Siegrist T., Steigerwald M. L., Li H., Nuckolls C. (2013). Chem. Sci..

[cit34] Fujikawa T., Segawa Y., Itami K. (2015). J. Am. Chem. Soc..

[cit35] Fujikawa T., Segawa Y., Itami K. (2017). J. Org. Chem..

[cit36] Hu Y., Wang X.-Y., Peng P.-X., Wang X.-C., Cao X.-Y., Feng X., Müllen K., Narita A. (2017). Angew. Chem., Int. Ed..

[cit37] Cruz C. M., Márquez I. R., Mariz I. F. A., Blanco V., Sánchez-Sánchez C., Sobrado J. M., Martín-Gago J. A., Cuerva J. M., Maçôas E., Campaña A. G. (2018). Chem. Sci..

[cit38] Reger D., Haines P., Heinemann F. W., Guldi D. M., Jux N. (2018). Angew. Chem., Int. Ed..

[cit39] Zhu Y., Xia Z., Cai Z., Yuan Z., Jiang N., Li T., Wang Y., Guo X., Li Z., Ma S., Zhong D., Li Y., Wang J. (2018). J. Am. Chem. Soc..

[cit40] Kato K., Segawa Y., Scott L. T., Itami K. (2018). Angew. Chem., Int. Ed..

[cit41] Cruz C. M., Castro-Fernández S., Maçôas E., Cuerva J. M., Campaña A. G. (2018). Angew. Chem., Int. Ed..

[cit42] Hartnett P. E., Matte H. S. S. R., Eastham N. D., Jackson N. E., Wu Y., Chen L. X., Ratner M. A., Chang R. P. H., Hersam M. C., Wasielewski M. R., Marks T. J. (2016). Chem. Sci..

[cit43] Meng D., Fu H., Xiao C., Meng X., Winands T., Ma W., Wei W., Fan B., Huo L., Doltsinis N. L., Li Y., Sun Y., Wang Z. (2016). J. Am. Chem. Soc..

[cit44] Li Y., Xu L., Liu T., Yu Y., Liu H., Li Y., Zhu D. (2011). Org. Lett..

[cit45] Martin R. H., Marchant M. J. (1974). Tetrahedron.

[cit46] Nakai Y., Mori T., Inoue Y. (2012). J. Phys. Chem. A.

[cit47] For example: Schaack C. Sidler E. Trapp N. Diederich F., Chem.–Eur. J., 2017, 23 , 14153 –14157 . 28922527 10.1002/chem.201703024

[cit48] Würthner F., Thalacker C., Diele S., Tschierske C. (2001). Chem.–Eur. J..

[cit49] Jiménez Á. J., Spänig F., Rodríguez-Morgade M. S., Ohkubo K., Fukuzumi S., Guldi D. M., Torres T. (2007). Org. Lett..

[cit50] Huang C., Barlow S., Marder S. R. (2011). J. Org. Chem..

[cit51] Buchta M., Rybáček J., Jančařík A., Kudale A. A., Buděšínský M., Chocholoušová J. V., Vacek J., Bednárová L., Císařová I., Bodwell G. J., Starý I., Stará I. G. (2015). Chem.–Eur. J..

[cit52] Hunter C. A., Sanders J. K. M. (1990). J. Am. Chem. Soc..

[cit53] Fuchter M. J., Weimar M., Yang X., Judge D. K., White A. J. P. (2012). Tetrahedron Lett..

[cit54] Fujino S., Yamaji M., Okamoto H., Mutai T., Yoshikawa I., Houjou H., Tani F. (2017). Photochem. Photobiol. Sci..

[cit55] Feng J., Zhang Y., Zhao C., Li R., Xu W., Li X., Jiang J. (2008). Chem.–Eur. J..

[cit56] Schlosser F., Moos M., Lambert C., Würthner F. (2013). Adv. Mater..

[cit57] Lefler K. M., Brown K. E., Salamant W. A., Dyar S. M., Knowles K. E., Wasielewski M. R. (2013). J. Phys. Chem. A.

[cit58] Zhao C., Zhang Y., Li R., Li X., Jiang J. (2007). J. Org. Chem..

[cit59] Yang Y., Correa Da Costa R., Fuchter M. J., Campbell A. J. (2013). Nat. Photonics.

[cit60] Kiran V., Mathew S. P., Cohen S. R., Hernández Delgado I., Lacour J., Naaman R. (2016). Adv. Mater..

