# Supplementary material for: Defying strain in the synthesis of an electroactive bilayer helicene
Source: Chem Sci. 2018 Nov 20;10(4):1029–34. doi: 10.1039/c8sc04216k (PMC6349016; doi:10.1039/c8sc04216k)
Supplement: Supplementary file 1 [file SC-010-C8SC04216K-s001.pdf]

Electronic Supplementary Material (ESI) for Chemical Science.
This journal is © The Royal Society of Chemistry 2018

Defying Strain in the Synthesis of an Electroactive Bilayer Helicene

Margarita Milton,[1†] Nathaniel J. Schuster,*[1†] Daniel W. Paley,[2] Raúl Hernández Sánchez,[3] Fay Ng,[1] Michael L. Steigerwald,*[1] and Colin Nuckolls*[1]

[1]Department of Chemistry, Columbia University, New York, New York 10027, USA
[2]Columbia Nano Initiative, Columbia University, New York, New York 10027, USA
[3]Department of Chemistry, University of Pittsburgh, Pittsburgh, Pennsylvania 15260, USA
[†]M.M. and N.J.S. contributed equally.

Email addresses of the corresponding authors:
njs2154@columbia.edu
mls2064@columbia.edu
cn37@columbia.edu

Table of Contents

**I.**	**Figures Referenced in the Communication**	**2**
**II.**	**General Experimental Details**	**8**
**III.**	**Synthesis and Characterization**	**11**
**IV.**	**Resolution of the Enantiomers of PPDH-OPe**	**21**
**V.**	**Absorbance and Fluorescence of PPDH, PPDH-OPe and NPDH in Cyclohexane**	**22**
**VI.**	**^{1}H-NMR and ^{13}C-NMR Spectra**	**23**
**VII.**	**DFT-Optimized Molecular Structures and TD-DFT Excited State Calculations**	**32**
**VIII.**	**Single-crystal X-ray Diffraction Data**	**65**
**IX.**	**References**	**67**

I. Figures Referenced in the Communication

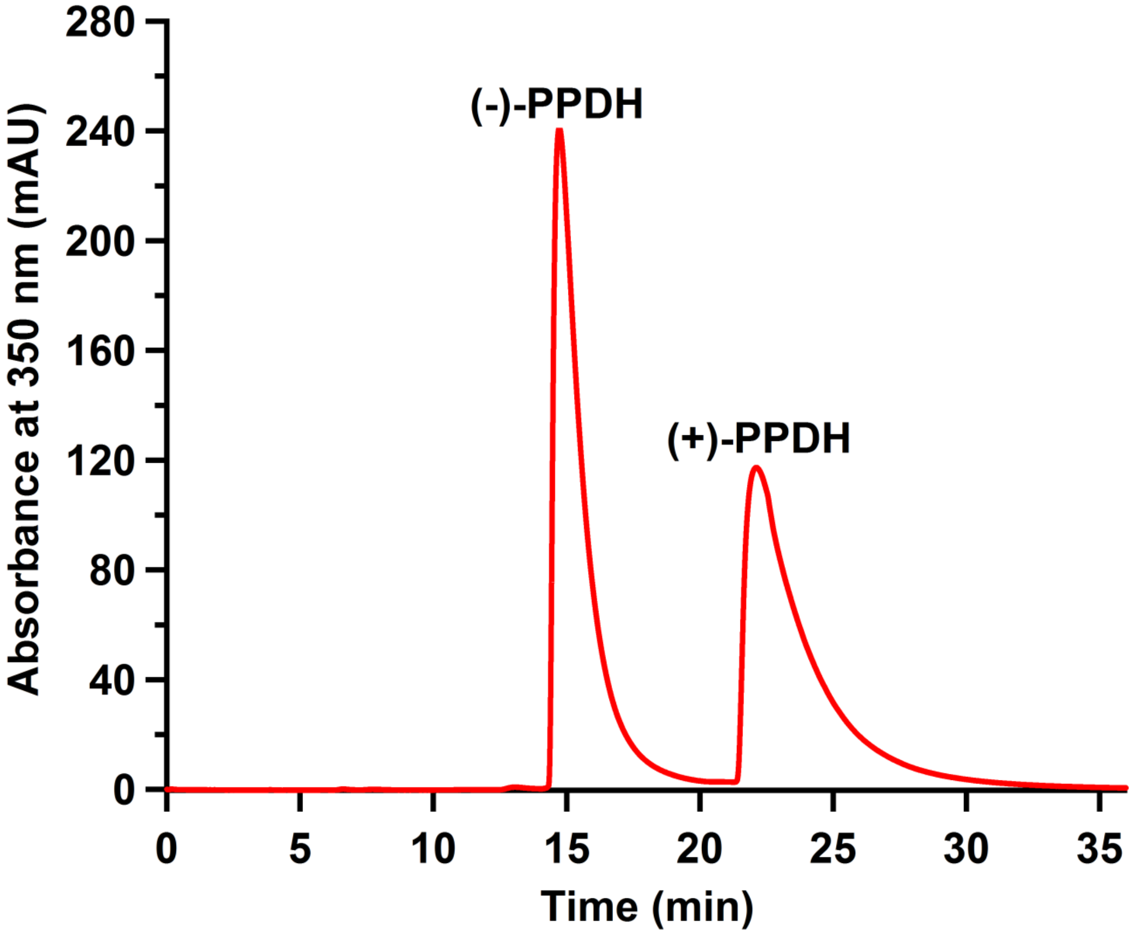

Figure S1. Resolution of the enantiomers of **PPDH** by chiral preparative HPLC. The enantiomers were resolved from 8 mg of racemate dissolved in 6 mL of 1:2 (v/v) dichloromethane/hexanes. This solution was injected in 1 mL aliquots onto a CHIRALPAK® IB-3 column (30 mm I.D. × 250 mm, 5 µm), with 20% dichloromethane/hexanes flowing at 28.5 mL/min at room temperature. The (-) and (+) correspond to the sign of the longest-wavelength Cotton effect observed for these enantiomers ($\Delta\varepsilon_{540\ nm}$ = -80 and +82 M^{-1} cm^{-1}).

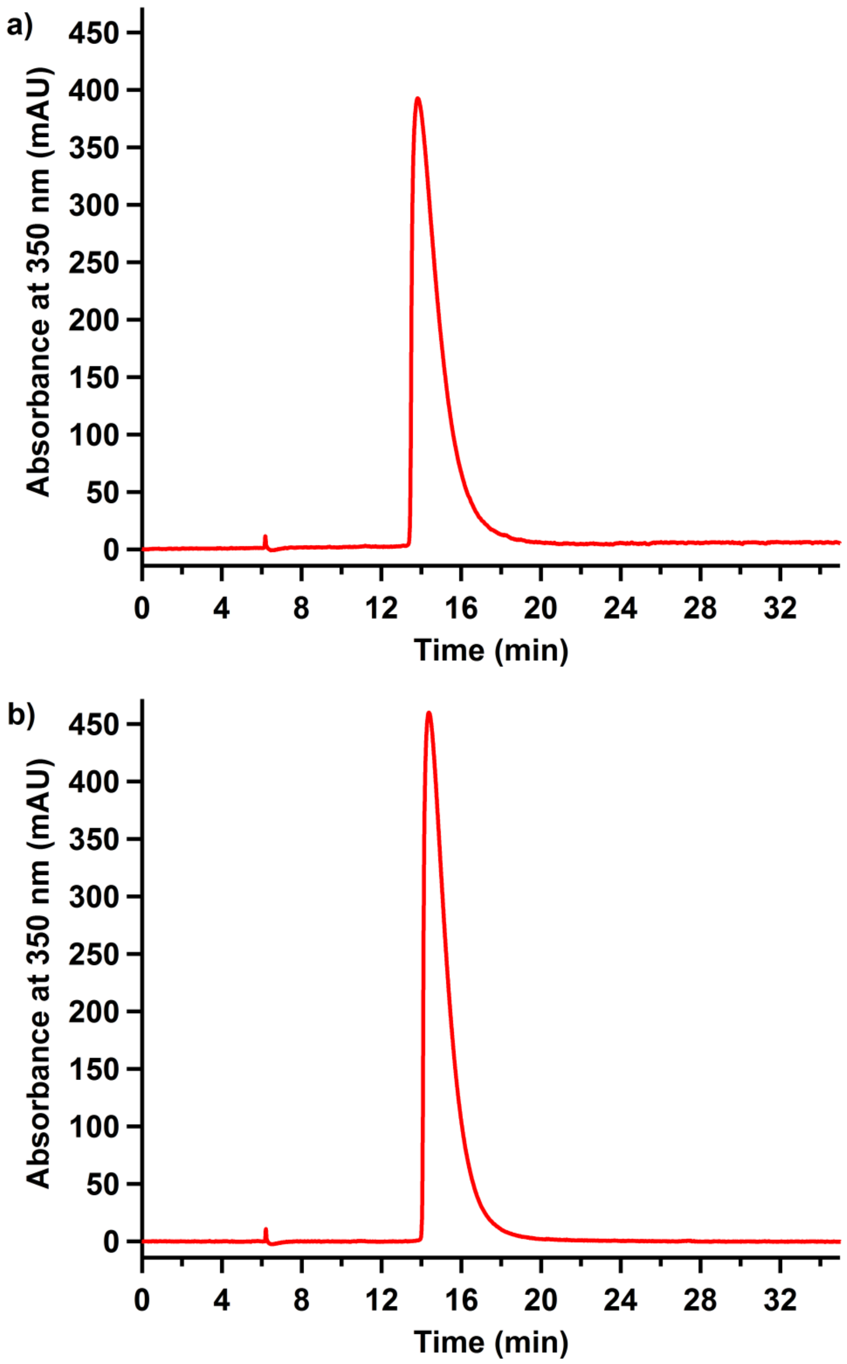

Figure S2. To show that the large extent of aryl surface overlap in **PPDH** inhibits racemization, we heated **(-)-PPDH** (1.7 mg) in diphenyl ether (0.75 mL) at 250 °C for 1 h. These are the HPLC traces of the solution before (a) and after (b) 1 h at 250 °C. This solution was injected in 10 µL aliquots onto a CHIRALPAK® IB-3 column (4.6 mm I.D. × 250 mm, 3 µm), with 20% dichloromethane/hexanes flowing at 1 mL/min at room temperature. The small peak at ~6 min corresponds to the complete elution of diphenyl ether. There is no trace of **(+)-PPDH**, confirming that **PPDH** does not racemize under these conditions.

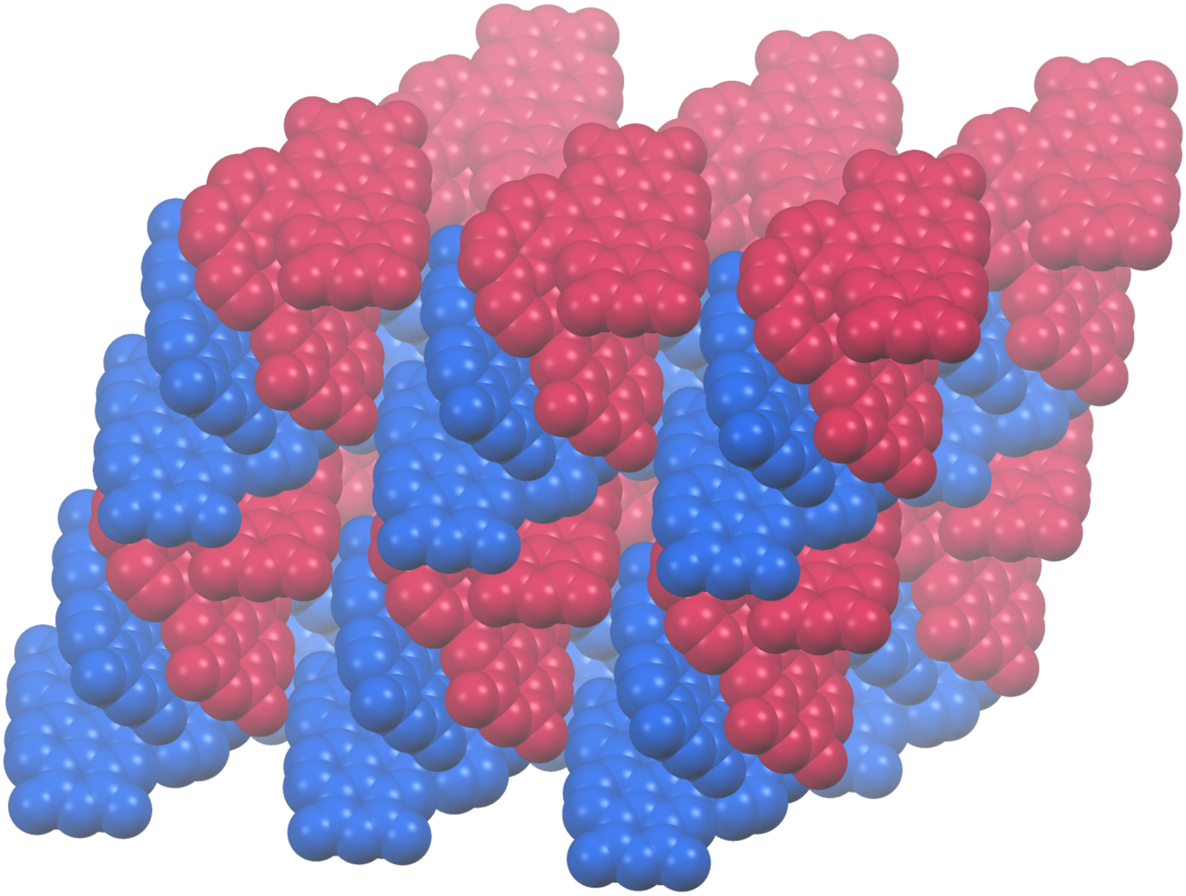

Figure S3. From SCXRD, racemic **PPDH** stacks into heterochiral columns (red, *M*-**PPDH**; blue, *P*-**PPDH**). Solvent, the $CH(C_5H_{11})_2$ chains, and hydrogen atoms have been hidden to provide a clear view of the aryl surface.

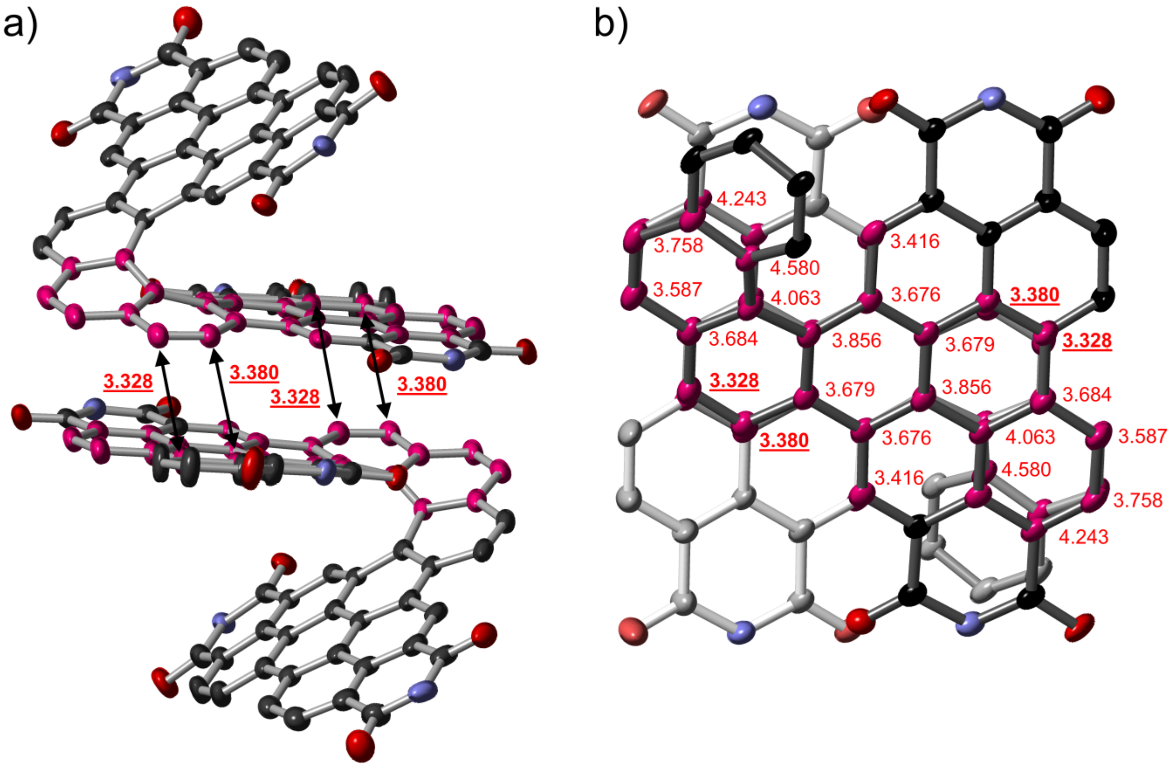

Figure S4. From SCXRD, the intermolecular junction between two molecules of **PPDH** consists of 24 pairs of overlapping π-bonded carbon atoms (shown in pink). (a) The four closest pairs, which approach to within twice the van der Waals radius of the carbon atom (*i.e.*, 3.4 Å), are designated with black arrows. (b) Top view of the same **PPDH** molecules as in (a), only the uppermost and bottommost PDI subunits have been removed for clarity. The distances between the overlapping atoms (in Å) are indicated to the right of each pair, and the four nearest neighbors are underlined in bold. Free solvent, the $CH(C_5H_{11})_2$ chains, and hydrogen atoms have been hidden to provide a clear view of the aryl surface. Thermal ellipsoids are set at 30% probability.

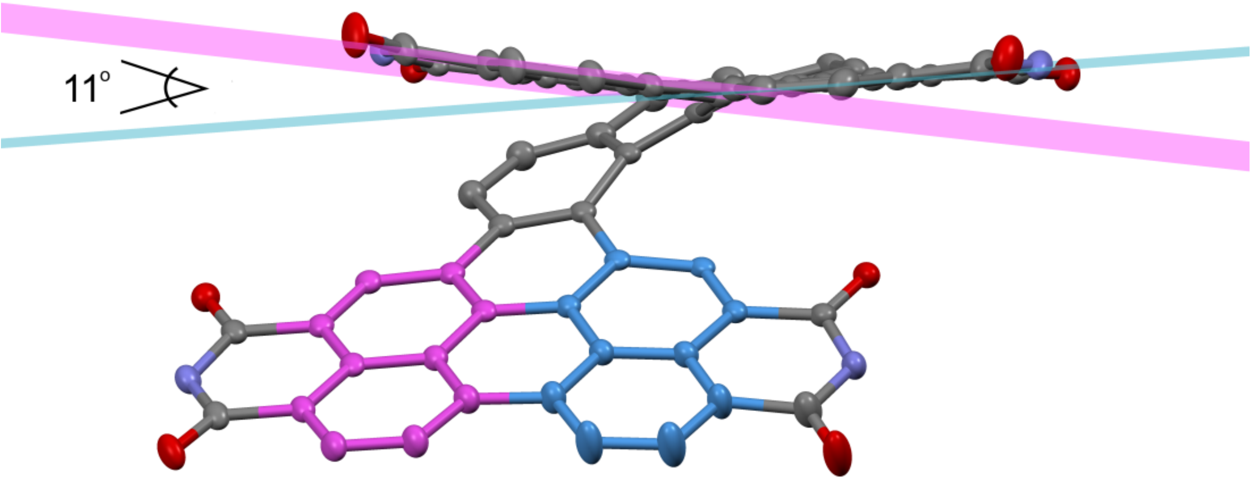

Figure S5. We define the bend angle of each PDI subunit in **PPDH** as the dihedral of the least-squares-fit planes defined by the pink and blue naphthalene fragments. From SCXRD, the bend angle of one PDI subunit in **PPDH** measures 11°, whereas the bend angle of the other PDI subunit (planes not shown) measures 9°. Free solvent, the $CH(C_5H_{11})_2$ chains, and hydrogen atoms have been hidden to provide a clear view of the aryl surface. Thermal ellipsoids are set at 30% probability.

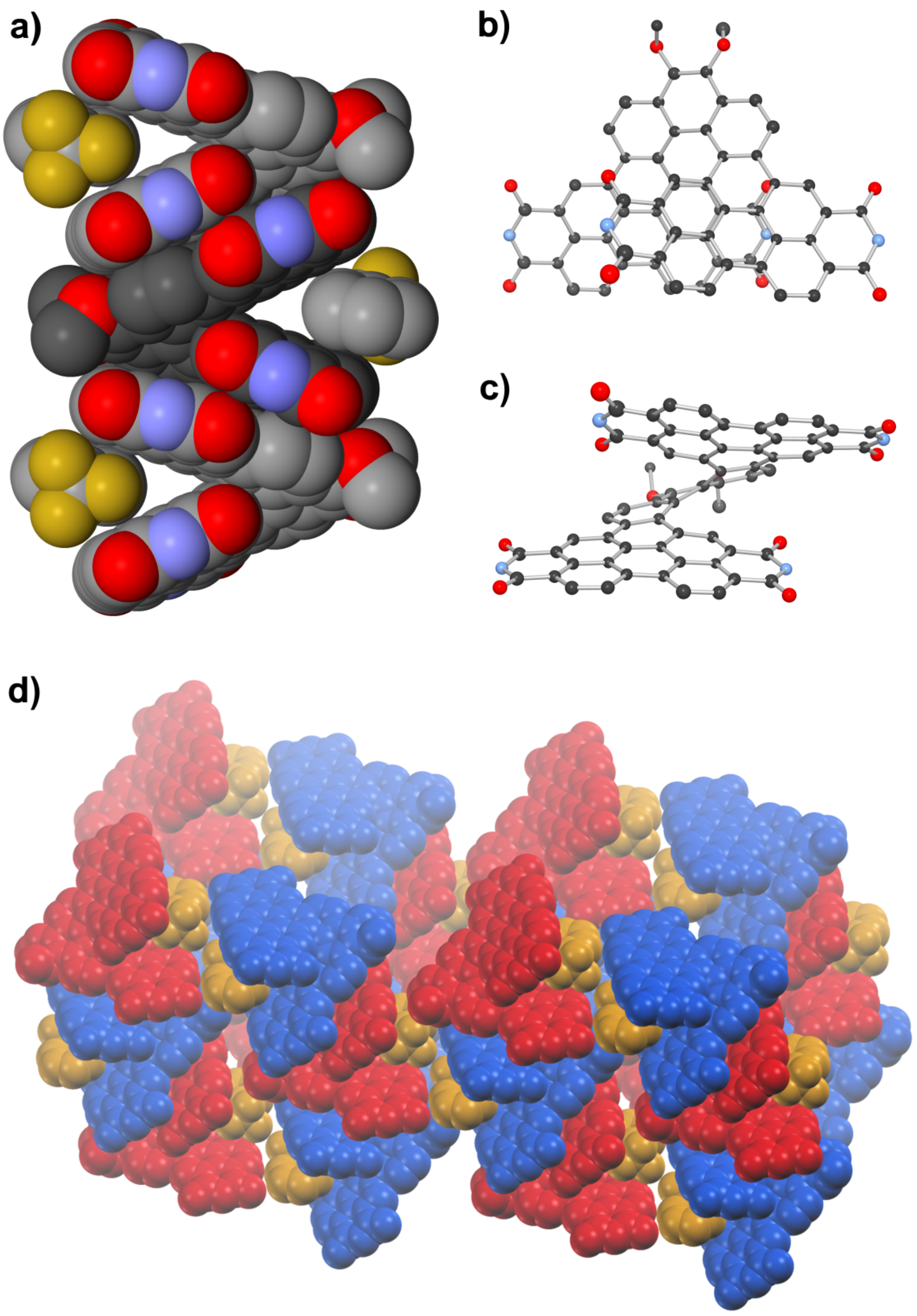

Figure S6. Structure of **PPDH-OPe** from SCXRD. (a) α,α,α-Trifluorotoluene – the solvent used in this crystallization – occupies the cavity between the PDI faces. (b and c) Different views of *M*-**PPDH-OPe**, with the solvent hidden. (d) **PPDH-OPe** packs into heterochiral columns (red, *M*-**PPDH-OPe**; blue, *P*-**PPDH-OPe**). The $CH(C_5H_{11})_2$ imide chains, C_4H_9 alkyl fragments of the pentoxy groups, and hydrogen atoms have been hidden to provide a clear view of the aryl surface.

II. General Experimental Details

Synthesis and Materials: All reactions were conducted in oven-dried glassware with magnetic stirring. Schlenk flasks were evacuated and backfilled with argon or nitrogen three times prior to use. Anhydrous tetrahydrofuran was obtained from a Glass Contour solvent system consisting of a Schlenk manifold with purification columns packed with activated alumina and supported copper catalyst. These solvents were dispensed from Pure-Pac™ containers purchased from Sigma-Aldrich. Anhydrous, Sure/Seal™ 1,4-dioxane was used as purchased from Sigma-Aldrich. Bis(pinacolato)diboron was used as purchased from Matrix Scientific. 1-Bromoperylene-3,4,9,10-tetracarboxylicdiimide (PDIBr) was prepared using a procedure developed by Rajasingh *et al.*[1] 3,6-Dibromophenanthrene was synthesized using a procedure by Scott *et al.*[2] 3,6-Dibromophenanthrene-9,10-quinone was synthesized using a procedure by Francke *et al.*[3] Potassium acetate was stored in a 200 °C oven for at least 24 h prior to use. Phenanthrene-9,10-quinone, 1-bromopentane, 2-isopropoxy-4,4,5,5-tetramethyl-1,3,2-dioxaborolane, and [1,1'-bis(diphenylphosphino)ferrocene]dichloropalladium were purchased from Sigma-Aldrich. All remaining reagents and solvents were purchased from commercial sources and used without additional purification. SATCO 55 W Bright White (3700 lumens) compact fluorescent lamps (CFLs) were used during the oxidative photocyclizations.

Purification: Automated flash chromatography was performed using a Teledyne Isco Combiflash Rf200 and Redisep Rf Silica columns. Silica plugs consisted of Silicycle SiliaFlash® P60 40-63 µm silica gel. Preparative thin-layer chromatography (TLC) employed Silicycle SiliaPlate™ Glass Backed TLC silica gel plates, 60 Å, 20 × 20 cm, 2000 µm thickness, F-254 indicator. Analytical TLC plates were cut from Silicycle SiliaPlate™ Glass Backed TLC Extra Hard Layer silica gel plates, 60 Å, 20 × 20 cm, 250 µm thickness, F-254 indicator.

NMR Spectroscopy: ^{1}H-NMR spectra were recorded on Bruker 500 MHz or 400 MHz spectrometers. ^{13}C-NMR spectra were recorded on Bruker 126 MHz or 100 MHz spectrometers with complete proton decoupling. Chemical shifts for protons are reported in parts per million (ppm) downfield from tetramethylsilane and are referenced to residual protium in the NMR solvent ($CHCl_3$: δ 7.26; $C_2H_2Cl_4$: δ 6.00). Chemical shifts for carbon are reported in ppm downfield from tetramethylsilane and are referenced to the carbon resonances of the solvent ($CDCl_3$: δ 77.16, $C_2D_2Cl_4$: δ 73.78). Data are represented as follows: chemical shift, multiplicity (s = singlet, d = doublet, dd = doublet of doublets, t = triplet, m = multiplet, bm = broad multiplet), coupling constants in hertz, and integration.

High-Resolution Mass Spectrometry (HRMS): HRMS data were obtained at the Columbia University Mass Spectrometry facility using a Waters XEVO G2XS instrument equipped with a

UPC^2 SFC inlet, electrospray (ESI) and atmospheric pressure chemical (APCI) ionization, and a QToF mass spectrometer.

UV-Visible-Near-Infrared Absorbance Spectroscopy: The absorbance spectra in Figures 2b and S11 were obtained on a Jasco V-750 spectrophotometer.

Fluorescence: The fluorescence spectra in Figures 2b and S11 were recorded using a Jasco FP-8300 spectrofluorometer. Fluorescence quantum yields were measured with a Jobin Yvon FluoroMax4 spectrofluorometer equipped with a Horiba Scientific integrating sphere. Very dilute solutions of **PPDH** and **PPDH-OPe** in cyclohexane (absorbance of ~0.08 at the long wavelength peaks of 489 and 494 nm, respectively) were used in these quantum yield experiments. At room temperature, the solutions were excited from 395-405 nm, and their emissions were measured from 475-675 nm.

Voltammetry: Cyclic voltammograms in Figure 4 were recorded on a CHI600C electrochemical workstation using Ag/AgCl as the reference electrode, glassy carbon (3 mm diameter) as the working electrode, and a platinum wire as the counter electrode. Experiments were performed under argon in dichloromethane with $[Bu_4N][PF_6]$ as the supporting electrolyte at a scan rate of 0.05 V/s.

Chiral Resolution: Racemic samples were analyzed by an Agilent 1200 Series analytical HPLC equipped with a diode array detector. Racemic samples were separated into their enantiomers by preparative HPLC using a Waters Prep150 LC System equipped with a UV-vis detector and an automated fraction collector. Further details are provided in the captions of Figures S1, S2, S9, and S10.

Electronic Circular Dichroism: The ECD spectra were recorded using a Jasco J-810 spectropolarimeter. A 10 mm path length high precision cell made of Quartz SUPRASIL® from Hellma Analytics was used in the collection of the spectra in Figure 2a.

Single-crystal X-ray Diffraction: Data for all compounds were collected on an Agilent SuperNova diffractometer using mirror monochromated Cu Kα radiation. Data collection, integration, scaling (ABSPACK), and absorption correction (face-indexed Gaussian integration[4] or numeric analytical methods[5]) were performed in CrysAlisPro (CrysAlisPro 1.171.38.41. Oxford Diffraction/Agilent Technologies UK Ltd, Yarnton, England). The structure was solved by intrinsic phasing using SHELXT[6] and refined with full-matrix least-squares on F^2 in SHELXL[7] using the OLEX2[8] interface. Successive cycles of least-square refinement followed by difference Fourier syntheses revealed the positions of the remaining non-hydrogen atoms. Hydrogen atoms were added in idealized positions.

Crystallographic data for **PPDH** and **PPDH-OPe** are given in Section VIII. Slow vapor diffusion of acetonitrile into a solution of **PPDH** in anisole afforded bright red prisms. Slow vapor diffusion of acetonitrile into a solution of **PPDH-OPe** in α,α,α-trifluorotoluene afforded small orange rods. The crystals were mounted on MiTeGen Kapton loops (polyimide) using paratone oil. Data were collected at 100 K.

Quantum Mechanical Calculations: All quantum chemical calculations were performed using Jaguar, version 8.3, Schrodinger, Inc., New York, NY, 2014.[9] The geometries were optimized in the gas phase using the B3LYP functional and the 6-31G** basis set. For the optimized geometries of **PPDH** and **PPDH-OPe**, the associated absorption spectra were calculated using the TD-DFT method that is included in the Jaguar package. The B3LYP functional and the 6-31G** basis set were used in these calculations. All alkyl chains ($CH[C_5H_{11}]_2$ and C_5H_{11}) were modeled as methyl groups.

The effects of dispersion were also assessed in the geometric optimizations of **PPDH** and **PPDH-OPe**. In their energy benchmark study of 47 density functionals, Goerigk and Grimme emphasized the efficacy of DFT-D3 in modeling noncovalent interactions.[10] They regard Zhao and Truhlar's PW6B95, coupled with the DFT-D3 correction, as "the most robust and very accurate general purpose hybrid-functional." Therefore, the PW6B95-D3/6-31G** level of theory was also used to optimize the gas-phase geometries of **PPDH** and **PPDH-OPe**. These optimized geometries diverge substantially from the SCXRD structures of **PPDH** and **PPDH-OPe**, which closely resemble the geometries predicted by B3LYP/6-31G** (Figure S23). Steric repulsion between the PDI subunits evidently predominates in these systems, which makes the B3LYP functional an appropriate choice for the calculation of strain (see Section VII).

III. Synthesis and Characterization

Scheme S1. Synthesis of **PPDH** and **5PPD**

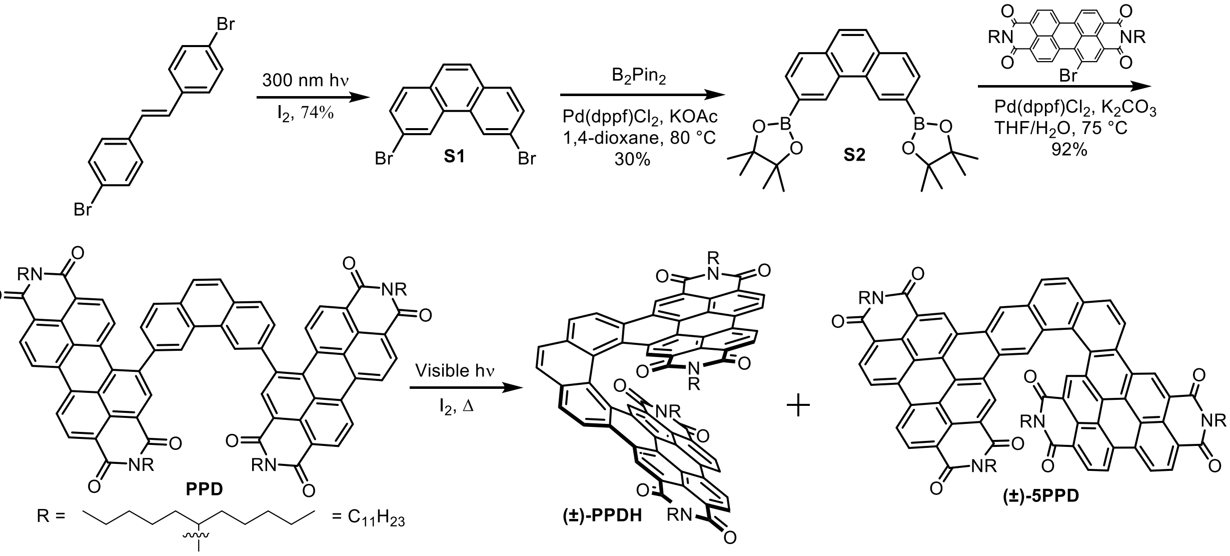

Scheme S2. Synthesis of **PPDH-OPe**

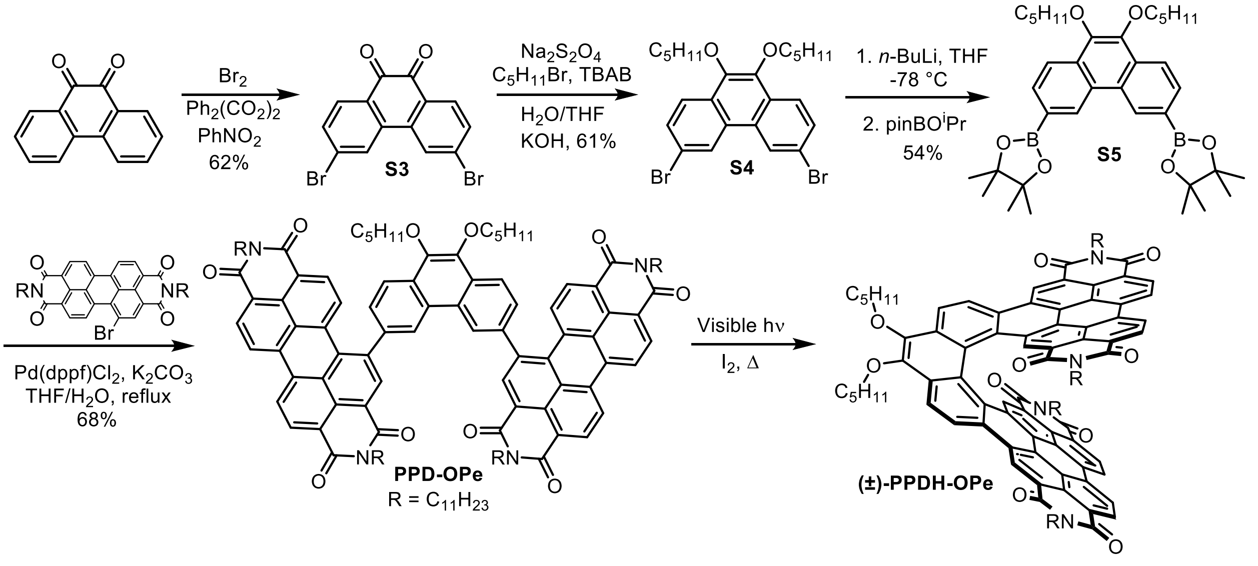

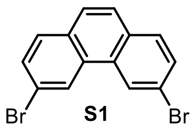

S1

3,6-Dibromophenanthrene (S1): *trans*-4,4'-Dibromostilbene (0.362 g, 1.07 mmol, 1 eq), iodine (0.603 g, 2.38 mmol, 2.22 eq), and propylene oxide (2.0 ml, 29 mmol, 27 eq) were combined with 310 mL of benzene in a 320-mL quartz round-bottom flask and sparged with nitrogen for 10 minutes. The flask was placed in a Rayonet photoreactor (The Southern New England Ultraviolet Company) with sixteen 300 nm lamps and stirred under UV light for 8 h. This reaction mixture was combined with another batch that started with 0.314 g of dibromostilbene. The solvent was removed under reduced pressure. The solid was purified by hot recrystallization from hexanes to yield 0.499 g of white needles (1.48 mmol, 74% over two batches). All spectra matched those reported in the literature.[2]

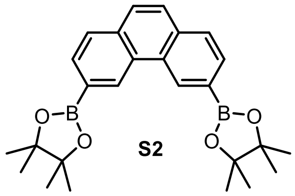

S2

S2: **S1** (0.107 g, 0.318 mmol, 1 eq), bis(pinacolato)diboron (0.176 g, 0.692 mmol, 2.18 eq), potassium acetate that had been dried in a 200 °C oven (0.243 g, 2.48 mmol, 7.79 eq), and [1,1'-bis(diphenylphosphino)ferrocene]dichloropalladium (0.0163 g, 0.0223 mmol, 7.00 mol%) were placed in an oven-dried 10-mL Schlenk flask, then evacuated and back-filled with nitrogen three times. In a separate oven-dried 10-mL round-bottom flask, 2 mL of anhydrous 1,4-dioxane were sparged for 8 min, then transferred to the reaction mixture and sparged for 3 min. The Schlenk flask was sealed with a glass stopcock and heated to 80 °C overnight, at which point it was added to 50 mL of deionized water. The aqueous layer was extracted with 3 x 50 mL of ethyl acetate. The organic layer was dried with $MgSO_4$, filtered, and the solvent removed with a rotary evaporator. Purification by column chromatography (SiO_2, gradient from 100% hexanes to 100% dichloromethane) afforded the white solid **S2** (0.0406 g, 0.0944 mmol, 30%). ^{1}H NMR (400 MHz, $CDCl_3$, 300 K) δ 9.30 (s, 2H), 8.02 (dd, J = 10.8, 0.9 Hz, 2H), 7.88 (d, J = 9.9 Hz, 2H), 7.77 (s, 2H), 1.45 (s, 24H). ^{13}C NMR (100 MHz, $CDCl_3$, 300 K) δ 134.24, 132.17, 130.37, 130.05, 128.20, 127.88, 84.16, 25.10. HRMS (APCI+) calculated m/z for $[C_{26}H_{32}B_2O_4+H]^+$ is 431.2560; found 431.2575.

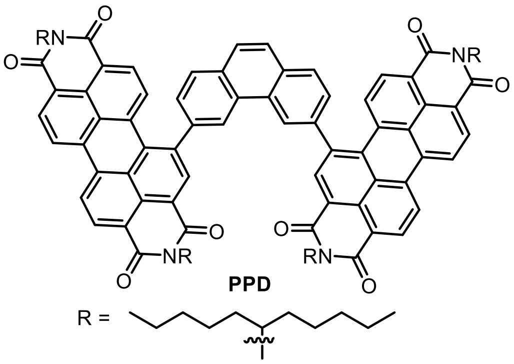

S3: PDIBr (0.152 g, 0.196 mmol, 2.12 eq), **S2** (0.0398 g, 0.0925 mmol, 1 eq), K_2CO_3 (0.300 g, 2.17 mmol, 24.4 eq), and [1,1'-bis(diphenylphosphino)ferrocene]dichloropalladium (0.0088 g, 0.012 mmol, 13 mol%) were placed in a 10-mL Schlenk flask, then evacuated and back-filled with nitrogen three times. In a separate 10-mL round-bottom flask, 3 mL of tetrahydrofuran and 1 mL of deionized H_2O were sparged with nitrogen for 10 min, then transferred to the reaction mixture and sparged for 4 min. The Schlenk flask was sealed with a glass stopcock and heated at 75 °C overnight, at which point it was added to 35 mL of deionized water. It was extracted with dichloromethane until the aqueous layer turned colorless. The organic layer was dried with Na_2SO_4, filtered, and the solvent removed with a rotary evaporator. Purification by column chromatography (SiO_2, gradient from 100% hexanes to 100% dichloromethane) afforded the dark red solid **S3** (0.133 g, 0.0847 mmol, 92%). ^{1}H NMR (500 MHz, $C_2D_2Cl_4$, 403 K) δ 9.12 (s, 2H), 8.77-8.65 (many overlapping signals, 10H), 8.13 (two overlapping doublets, 4H), 8.06 (s, 2H), 7.97 (d, *J* = 8.3 Hz, 2H), 7.69 (d, *J* = 8.2 Hz, 2H), 5.17 (bm, 4H), 2.25 (bm, 8H), 1.94 (bm, 8H), 1.34 (broad, overlapping signals, 55H), 0.88 (broad, overlapping signals, 25H). ^{13}C NMR (126 MHz, $C_2D_2Cl_4$, 403 K) δ 163.73, 163.70, 163.45, 141.39, 141.32, 135.96, 134.77, 134.31, 134.19, 132.54, 132.28, 131.79, 130.66, 130.51, 130.00, 129.94, 129.07, 128.58, 128.08, 127.56, 127.44, 123.91, 123.57, 123.11, 122.98, 122.79, 122.52, 122.32, 74.03, 54.89, 54.70, 32.34, 31.39, 26.36, 22.06, 13.42. HRMS (APCI+) calculated m/z for $[C_{106}H_{114}N_4O_8+Na]^+$ is 1594.8562; found 1594.8595.

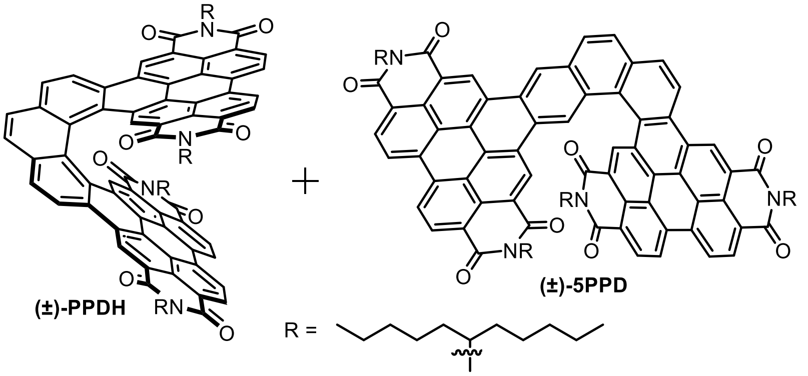

PPDH and **5PPD**: **PPD** (0.0308 g, 0.0196 mmol, 1 eq), iodine (0.0322 g, 0.127 mmol, 6.48 eq), and K_2CO_3 (0.545 g, 3.94 mmol, 201 eq) were dissolved in 98 mL of benzene in a 150-mL round-bottom flask. The solution was sparged with nitrogen for 30 min and left under positive pressure of nitrogen while being irradiated by two 55 W CFLs for 24 h at 30 °C (the temperature

to which the light bulbs heated the solution) in a pristine oil bath. The solvent was removed by rotary evaporation and the material was loaded onto a small silica plug and dried with air. The plug was flushed with acetonitrile (40 mL) to remove iodine and benzene (but not the products, which are insoluble in acetonitrile and stay on the baseline). The mixture of products was brought down with 9:1 (v/v) dichloromethane/ethyl acetate and the solvent was removed by rotary evaporation. ^{1}H NMR was taken of this mixture in $C_2D_2Cl_4$ at 393 K (Figure S7). To isolate the products, the mixture was loaded onto a plug again and flushed with 9:1 (v/v) dichloromethane/hexanes, then dichloromethane. These dichloromethane washes contained only **5PPD** (0.0108 g, 0.0689 mmol, 35% isolated yield). **PPDH** was brought down with 9:1 (v/v) dichloromethane/ethyl acetate and the solvent removed by rotary evaporation to give a red solid (0.0193 g, 0.0123 mmol, 63% isolated yield). **PPDH**: ^{1}H NMR (500 MHz, $C_2D_2Cl_4$, 393 K) δ 10.27 (s, 2H), 9.48 (d, J = 8.7 Hz, 2H), 9.08 (d, J = 8.1 Hz, 2H), 8.97 (d, J = 8.2 Hz, 2H), 8.75 (two overlapping doublets, 4H), 8.61 (s, 2H), 8.39 (d, J = 8.1 Hz, 2H), 8.33 (s, 2H), 5.35 (bm, 2H), 4.50 (bm, 2H), 2.35-2.20 (several overlapped signals, 8H), 1.69-0.79 (many overlapped signals, 80H). ^{13}C NMR (126 MHz, $C_2D_2Cl_4$, 393 K) δ 164.16, 163.97, 162.72, 162.25, 132.84, 131.82, 129.91, 129.68, 129.66, 129.01, 128.49, 127.84, 127.72, 127.17, 126.25, 125.44, 125.31, 125.09, 123.80, 123.76, 123.43, 123.01, 122.68, 122.54, 121.87, 121.48, 119.47, 74.03, 55.35, 53.55, 32.73, 32.57, 31.61, 31.55, 31.26, 30.91, 30.78, 30.65, 26.85, 26.73, 25.95, 25.88, 22.22, 22.06, 13.56, 13.45, 13.41. HRMS (APCI+) calculated m/z for $[C_{106}H_{110}N_4O_8+H]^+$ is 1568.8430; found 1568.8474. **5PPD**: ^{1}H NMR (500 MHz, $C_2D_2Cl_4$, 393 K) δ 10.58 (s, 1H), 10.43 (s, 1H), 10.32 (s, 1H), 10.17 (s, 1H), 10.00 (s, 1H), 9.44-9.41 (three overlapping doublets, 3H), 9.19 (d, J = 8.2 Hz, 1H), 9.14-9.09 (three overlapping doublets, 3H), 9.04 (s, 1H), 9.02 (d, J = 8.0 Hz, 1H), 8.92 (d, J = 8.0 Hz, 1H), 8.67 (d, J = 8.7 Hz, 1H), 8.54 (d, J = 8.6 Hz, 1H), 8.38 (d, J = 8.7 Hz, 1H), 5.43 (bm, 2H), 4.98 (bm, 1H), 4.66 (bm, 1H), 2.46 (bm, 4H), 2.11-1.90 (overlapping peaks, 8H), 1.58-1.36 (overlapping peaks, 44 H), 0.98-0.48 (overlapping peaks, 32H). ^{13}C NMR (126 MHz, $C_2D_2Cl_4$, 393 K) δ 164.42, 164.35, 164.29, 164.10, 163.80, 163.76, 163.67, 162.96, 134.13, 133.77, 133.64, 133.59, 133.32, 133.23, 132.29, 130.14, 129.54, 129.48, 129.39, 128.86, 128.86, 128.57, 128.10, 127.95, 127.85, 127.73, 127.62, 127.40, 127.29, 126.58, 126.52, 125.02, 125.01, 124.94, 124.88, 124.83, 124.70, 124.50, 123.55, 123.52, 123.24, 123.01, 122.88, 122.81, 74.03, 55.14, 53.79, 32.52, 32.47, 32.07, 31.75, 31.53, 31.41, 30.83, 26.53, 26.42, 25.68, 25.62, 22.31, 22.23, 22.08, 21.63, 21.60, 13.65, 13.59, 13.24, 13.19. HRMS (APCI+) calculated m/z for $[C_{106}H_{110}N_4O_8+H]^+$ is 1568.8430; found 1568.8464.

This reaction was repeated with **PPD** (0.0331 g, 0.0211 mmol, 1 eq), iodine (0.0371 g, 0.146 mmol, 6.94 eq), and K_2CO_3 (0.634 g, 4.59 mmol, 218 eq) in 106 mL of benzene at 70 °C for 24 h in a pristine oil bath. Isolation and purification followed the same procedure as above to give 0.0274 g (0.0175 mmol, 83%) of **PPDH**.

This reaction was repeated with **PPD** (0.0268 g, 0.0171 mmol, 1 eq), iodine (0.0291 g, 0.115 mmol, 6.72 eq), and K_2CO_3 (0.476 g, 3.44 mmol, 202 eq) in 90 mL of chlorobenzene at 110 °C for 24 h in a pristine oil bath. Isolation and purification followed the same procedure as above to give 0.0242 g (0.0154 mmol, 91%) of **PPDH**.

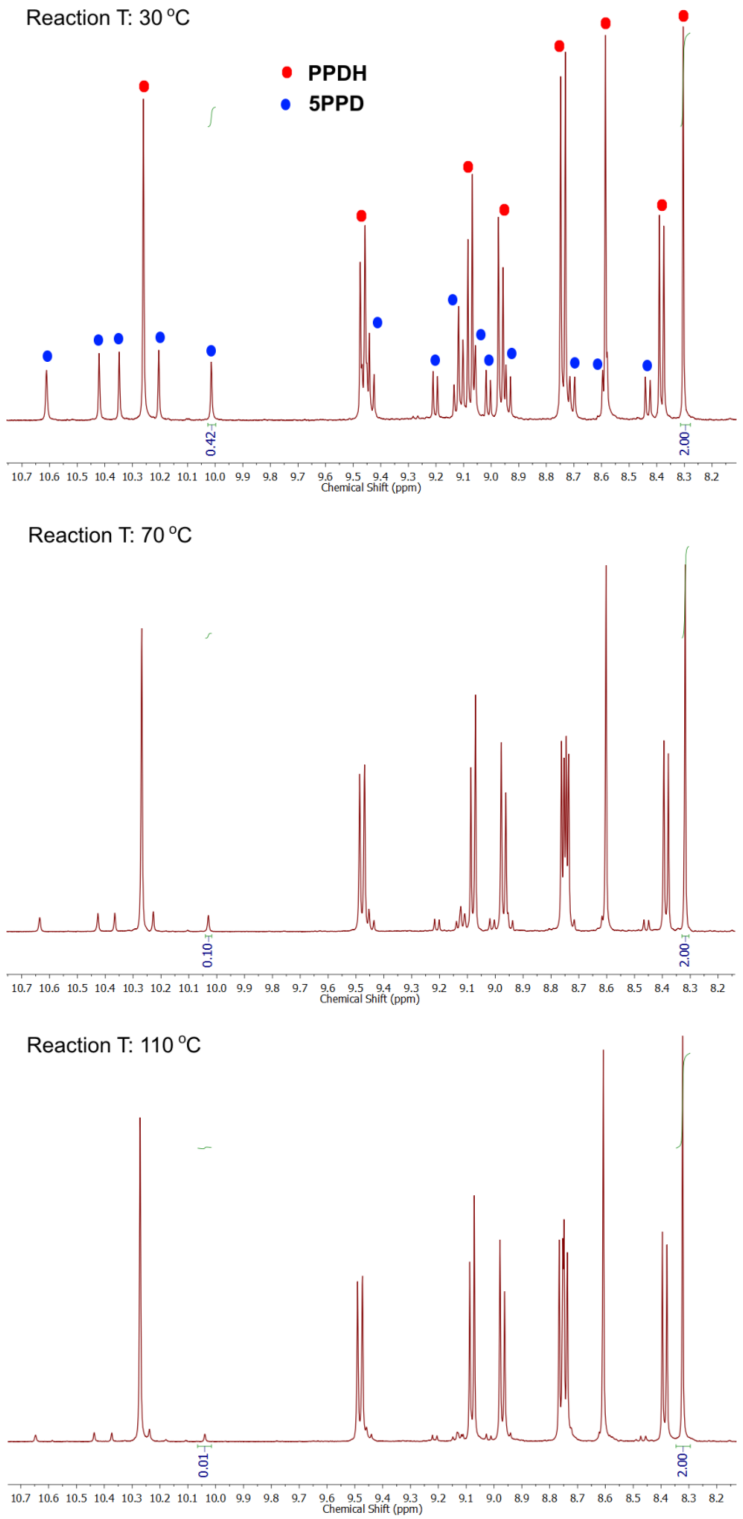

Figure S7. ^{1}H-NMR spectra (500 MHz, $C_2D_2Cl_4$, 393 K) of the product mixtures (after an acetonitrile plug to remove iodine and benzene) resulting from the oxidative photocyclization of **PPD** at different temperatures. The integral shown for **PPDH** corresponds to two protons and the integral shown for **5PPD** corresponds to one proton.

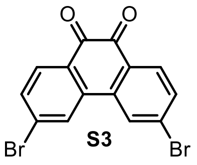

3,6-Dibromophenanthrene-9,10-quinone (S3): This molecule was synthesized according to a published procedure.[3] All spectra matched those previously reported.

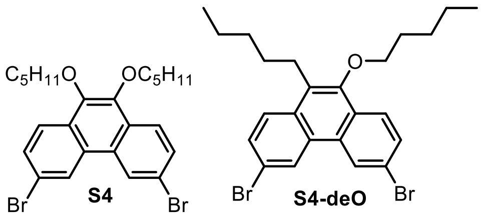

S4: 3,6-Dibromophenanthrene-9,10-quinone **S3** (5.45 g, 14.9 mmol, 1 eq), $Na_2S_2O_4$ (25.9 g, 149 mmol, 10.0 eq), and tetrabutylammonium bromide (4.82 g, 15.0 mmol, 1.00 eq) were placed in a 500-mL round-bottom flask. Tetrahydrofuran (110 mL) and deionized water (110 mL) were added. The flask was capped and shaken for 6 min. 1-Bromopentane (8.1 mL, 65 mmol, 4.4 eq) was added, followed by KOH (22.0 g, 392 mmol, 26.3 eq) in 110 mL of water. The mixture became dark. It was allowed to stir for 48 h, after which it was judged complete by TLC (95:5 [v/v] hexanes/ethyl acetate). The aqueous layer was extracted with 3 x 200 mL of ethyl acetate. The organic layers were combined and washed with water (2 x 200 mL), brine (1 x 100 mL), dried with Na_2SO_4, decanted, and the solvent removed with a rotary evaporator to yield a brown oil. Ethanol was added to precipitate the product, which was further washed with ethanol, leaving 4.59 g (9.03 mmol, 61%) of a pale yellow solid. This product contained trace (<5% by NMR) **S4-deO**, a molecule that lacks one oxygen. **S4-deO** could not be removed from **S4** by silica gel column chromatography or recrystallization, so the product was carried forward. The monodeoxygenated impurity was removed by preparative HPLC after the oxidative photocyclization of **PPD-OPe**. An analytically pure sample of **S4** and (and **S4-deO**, whose ^{1}H-NMR spectrum is included in Section VI) were obtained by preparative TLC (cyclohexane). ^{1}H NMR (500 MHz, $CDCl_3$, 300 K) δ 8.64 (s, 2H), 8.09 (d, J = 8.7 Hz, 2H), 7.70 (d, J = 8.8 Hz, 2H), 4.18 (t, J = 6.7 Hz, 4H), 1.91 (m, 4H), 1.54 (m, 4H), 1.44 (m, 4H), 0.97 (t, J = 7.3, 6H). ^{13}C NMR (125 MHz, $CDCl_3$, 300 K) δ 143.28, 130.58, 129.00, 128.88, 125.52, 124.33, 120.44, 73.87, 30.26, 28.51, 22.72, 14.22. HRMS (APCI+) calculated m/z for $[C_{24}H_{28}Br_2O_2]^+$ is 508.0436; found 508.0456.

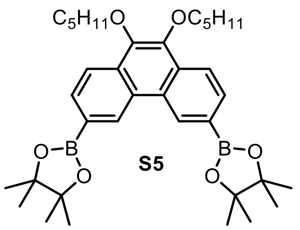

S5: A 50-ml Schlenk flask was charged with **S4** (0.982 g, 1.93 mmol, 1 eq), tetrahydrofuran (36 mL), and *N*,*N*,*N'*,*N'*-tetramethylethylenediamine (0.64 mL, 4.3 mmol, 2.2 eq). The flask was immersed in an acetone/dry ice bath. After being cooled for 15 min, 1.59 M *n*-butyllithium in hexanes (2.7 mL, 4.3 mmol, 2.2 eq) was added dropwise over 9 min and the solution was allowed to stir for an hour. 2-Isopropoxy-4,4,5,5-tetramethyl-1,3,2-dioxaborolane (0.91 mL, 4.4 mmol, 2.3 eq) was added and the solution was stirred cold for 20 min, then warmed up to room temperature. The reaction was monitored by TLC (95:5 [v/v] hexanes:ethyl acetate) and judged complete after 1 h. The reaction mixture was poured into saturated aqueous NH_4Cl (100 mL) and extracted with ethyl acetate (3 x 100 mL). The organic layer was dried with $MgSO_4$, filtered, and the solvent removed with a rotary evaporator to yield a slowly solidifying brown solid. The solid was recrystallized twice from ethanol to give 0.62 g (1.04 mmol, 54%) of white crystals. ^1H NMR (500 MHz, $CDCl_3$, 323 K) δ 9.22 (s, 2H), 8.23 (d, *J* = 8.2 Hz), 8.02 (d, *J* = 8.2 Hz), 4.21 (t, *J* = 6.7 Hz, 4H), 1.91, (m, 4H), 1.55 (m, 4H), 1.44-1.40 (two overlapped peaks, 28H), 0.96 (t, *J* = 7.3 Hz, 6H). ^{13}C NMR (126 MHz, $CDCl_3$, 323 K) δ 144.27, 132.33, 131.77, 130.25, 128.40, 121.46, 84.12, 73.79, 30.30, 28.54, 25.09, 22.73, 14.24. HRMS (APCI+) calculated m/z for $[C_{36}H_{52}B_2O_6+H]^+$ is 603.4035; found 603.4047.

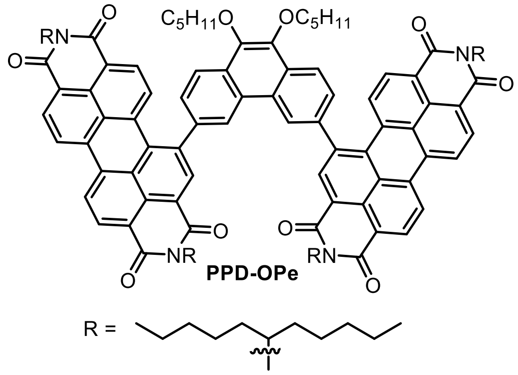

PPD-OPe: PDIBr (0.498 g, 0.640 mmol, 2.17 eq), **S5** (0.178 g, 0.295 mmol, 1 eq), K_2CO_3 (1.32 g, 9.53 mmol, 32.3 eq) and [1,1'-bis(diphenylphosphino)ferrocene]dichloropalladium (48.1 mg, 0.0657 mmol, 22.3 mol%) were placed in a two-neck 50-mL round-bottom flask fitted with a reflux condenser. The flask was evacuated and backfilled with nitrogen three times. In a separate flask, tetrahydrofuran (18 mL) and H_2O (2 mL) were sparged for 30 min with nitrogen. The solvents were transferred into the flask with the solids by syringe and sparged for 15 min. The reaction mixture was heated to reflux for 16 h, at the end of which it was added to 50 mL of deionized water. The aqueous layer was extracted with dichloromethane until it became clear

(~100 mL). The organic layer was dried with $MgSO_4$, filtered, and the solvent removed with a rotary evaporator. Purification by column chromatography (SiO_2, gradient from 100% hexanes to 90% dichloromethane) afforded a red solid (0.348 g, 0.200 mmol, 68%). ^{1}H NMR (500 MHz, $C_2D_2Cl_4$, 403 K) δ 9.06 (s, 2H), 8.76-8.65 (several overlapped peaks, 10H), 8.51 (d, *J* = 8.3 Hz, 2H), 8.16 (d, *J* = 8.1 Hz, 2H), 8.01 (d, *J* = 8.2 Hz, 2H), 7.70 (d, *J* = 8.2 Hz, 2H), 5.18 (bm, 4H), 4.51 (bm, 4H), 2.25 (bm, 8H), 2.11 (m, 4H), 1.94 (bm, 8H), 1.73 (m, 4H), 1.61 (m, 4H), 1.35 (bm, 48H), 1.09 (t, *J* = 7.1 Hz, 6H), 0.89 (bm, 24H). ^{13}C NMR (126 MHz, $C_2D_2Cl_4$, 403 K) δ 163.72, 163.47, 143.90, 141.48, 140.25, 136.06, 134.75, 134.42, 134.22, 132.49, 130.65, 130.61, 130.16, 130.03, 129.94, 129.88, 129.08, 128.53, 128.08, 127.45, 127.41, 124.62, 123.87, 123.56, 123.10, 122.91, 122.71, 122.36, 122.29, 73.95, 54.84, 54.67, 32.34, 31.40, 29.99, 28.26, 26.35, 22.24, 22.08, 13.54, 13.44. HRMS (APCI$^+$) calculated m/z for $[C_{116}H_{134}N_4O_{10}+H]^+$ is 1745.0206; found 1745.0222.

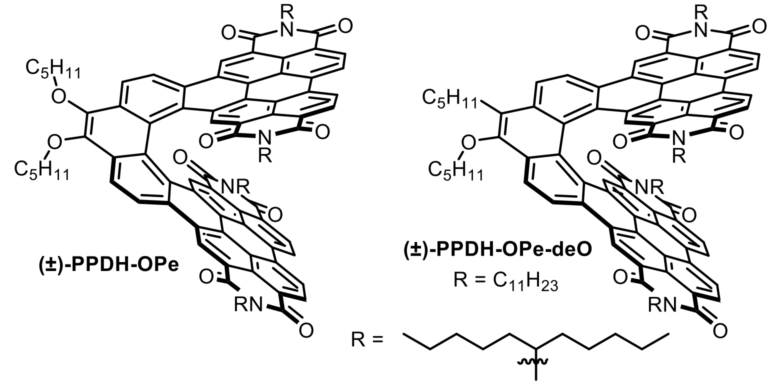

PPDH-OPe: PPD-OPe (0.0291 g, 0.0167 mmol, 1 eq), iodine (0.0285 g, 0.112 mmol, 6.73 eq), and K_2CO_3 (0.460 g, 3.33 mmol, 200 eq) were dissolved in 78 mL of benzene in a 150-mL round-bottom flask. The solution was sparged with nitrogen for 30 min and left under positive pressure of nitrogen while being irradiated by two 55 W CFL light bulbs for 76 h in a pristine oil bath. The amount of mono-cyclized intermediate decreased over this time, as observed by TLC (4:1 [v/v] dichloromethane/hexanes). However, the amount of decomposition also increased, so the reaction was halted. The solvent was removed by rotary evaporation and the material was loaded onto a small silica plug and dried with air. The plug was flushed with acetonitrile (40 mL), followed by 9:1 (v/v) dichloromethane/hexanes, then dichloromethane. These dichloromethane washes contained the mono-cyclized intermediate and decomposition (which have a combined mass of 4 mg. The decomposition product has broad, unidentifiable peaks in its ^{1}H-NMR spectrum and a mass that corresponds to loss of the pentyl groups). **PPDH-OPe** was brought down with 9:1 (v/v) dichloromethane/ethyl acetate and the solvent removed by rotary evaporation to give a red solid (0.0256 g, 88%). Due to the difficulty of separating **S4** from **S4-deO** on a large scale, **PPDH-OPe** contains a small amount of the [7]helicene **PPDH-OPe-deO** (<5% of the product by NMR [Figure S8]). The separation between such similar molecules by HPLC is poor and the recovery is 76%, with the rest remaining in mixed fractions (Figure S9). ^{1}H NMR (500 MHz, $C_2D_2Cl_4$, 393 K) δ 10.26 (s, 2H), 9.47 (d, *J* = 9.0 Hz, 2H), 9.14 (d, *J* = 8.9

Hz, 2H), 9.08 (d, J = 8.1 Hz, 2H), 8.97 (d, J = 8.2 Hz, 2H), 8.74 (d, J = 8.2 Hz, 2H), 8.37 (d, J = 8.1 Hz, 2H), 8.29 (s, 2H), 5.35 (m, 2H), 4.84 (m, 2H), 4.69 (m, 2H), 4.49 (m, 2H), ~2.35-2.20 (bm, 12H), 1.89 (m, 4H), ~1.76-0.79 (several overlapping signals, 90H). ^{13}C NMR (126 MHz, $C_2D_2Cl_4$, 363 K) δ 164.17, 164.01, 162.71, 162.35, 145.67, 132.79, 131.72, 130.19, 129.56, 129.28, 128.42, 127.88, 127.61, 127.10, 126.36, 125.44, 125.01, 124.15, 123.70, 123.50, 123.40, 123.00, 122.98, 122.59, 122.50, 122.43, 121.78, 121.37, 119.33, 74.76, 55.31, 53.53, 32.74, 32.55, 31.62, 31.55, 31.28, 30.81, 30.64, 30.15, 28.38, 26.87, 26.73, 25.98, 25.91, 22.39, 22.23, 22.10, 22.06, 13.70, 13.57, 13.48, 13.42. HRMS (ESI+) calculated m/z for $[C_{116}H_{130}N_4O_{10}+Na]^+$ is 1762.9712; found 1762.9644. For **PPDH-OPe-deO**, HRMS (APCI+) calculated m/z for $[C_{116}H_{130}N_4O_9+H]^+$ is 1724.9944; found 1724.9944.

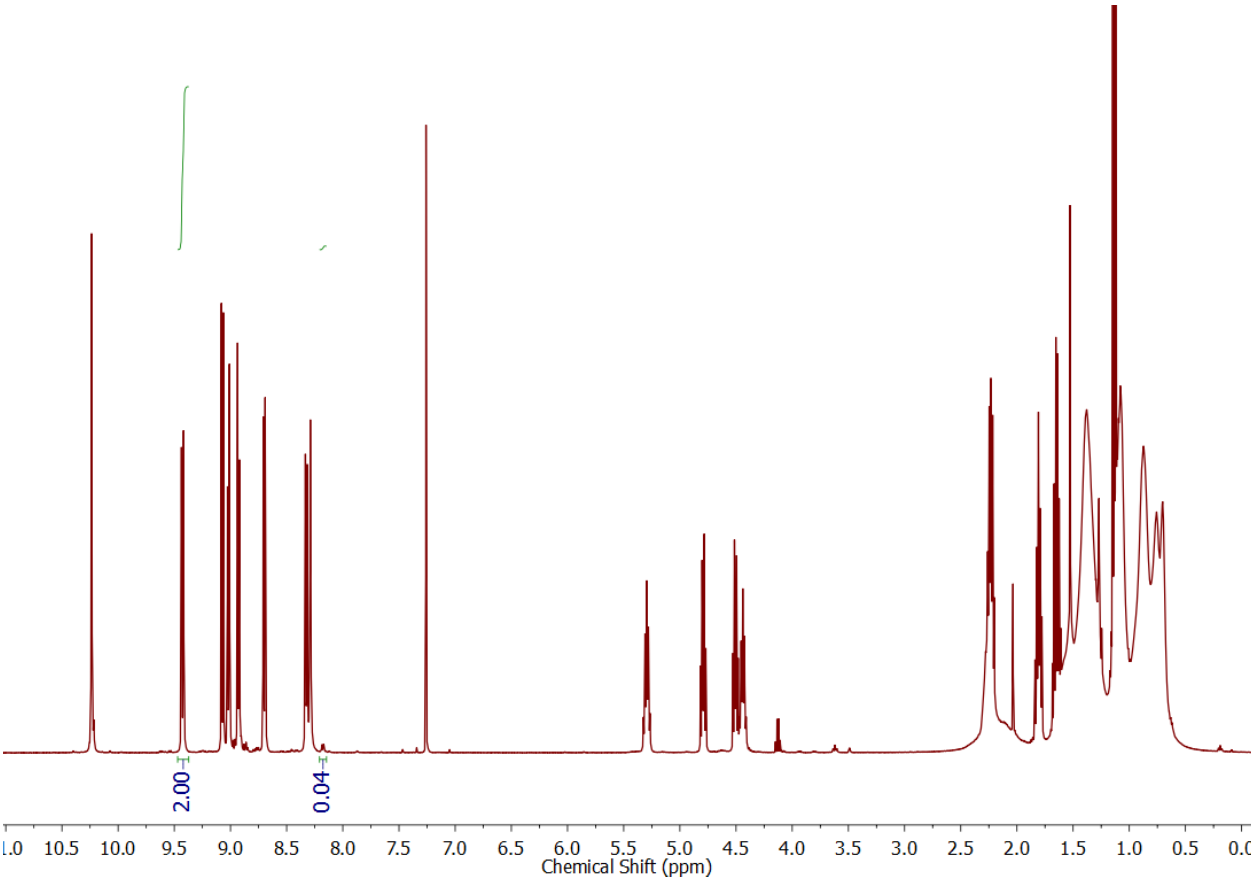

Figure S8. ^{1}H-NMR spectrum ($CDCl_3$, 323 K) of the mixture of **PPDH-OPe** and **PPDH-OPe-DeO** after a dichloromethane wash to remove the mono-cyclized intermediate and decomposition byproducts.

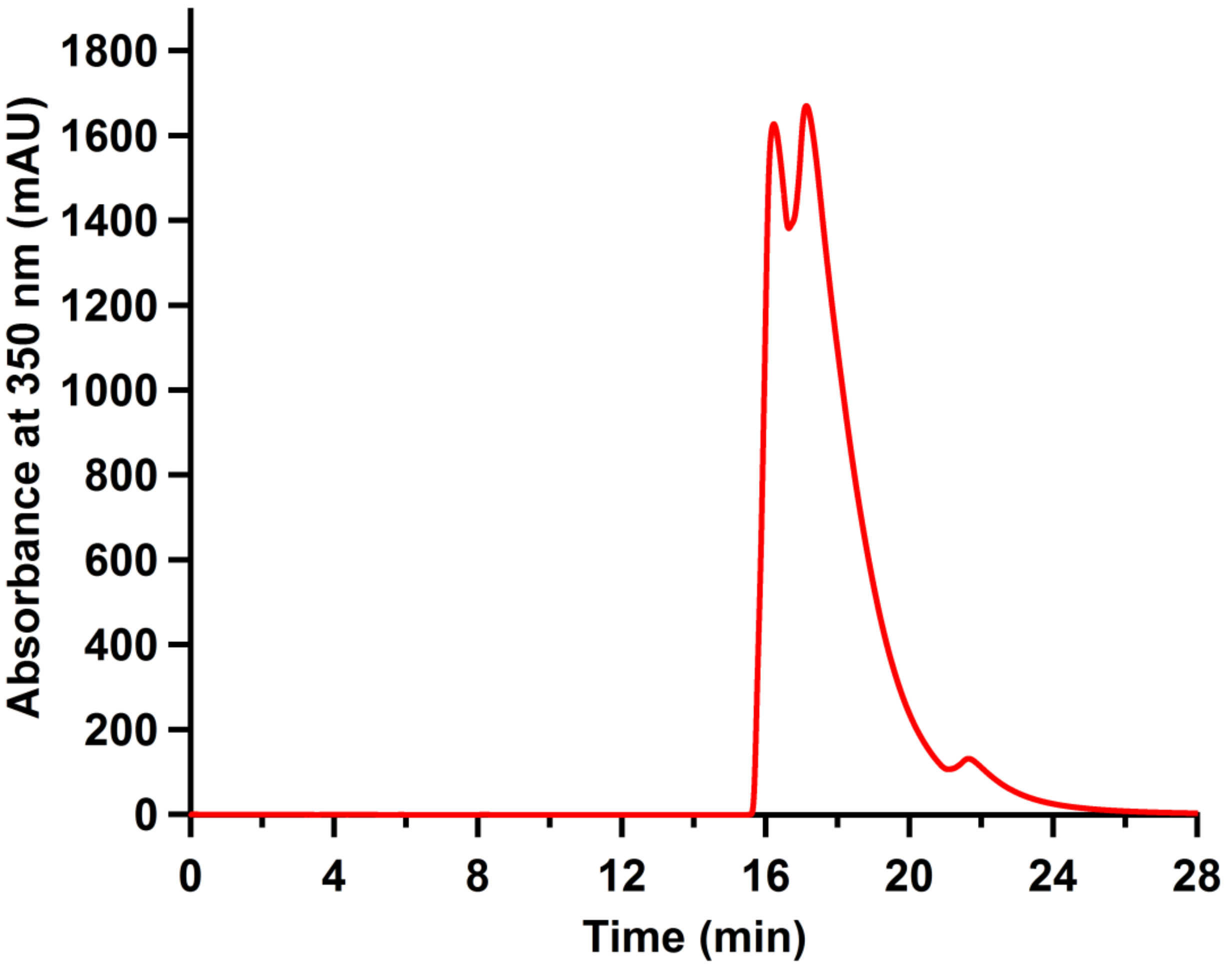

Figure S9. Separation of **PPDH-OPe** from the mono-deoxygenated impurity, **PPDH-OPe-deO**, by preparative HPLC. **PPDH-OPe** (~80 mg) was dissolved in 8 mL of 1:3 (v/v) dichloromethane/hexanes and injected in 1000 μL aliquots onto a CHIRALPAK® IA-3 column (21 mm I.D. × 250 mm, 5 μm), with 18% dichloromethane/hexanes flowing at 18 mL/min at room temperature. **PPDH-OPe-deO** is the small peak at 22 min. The splitting of the major peak is due to minimal separation of the enantiomers of **PPDH-OPe** on this chiral column.

IV. Resolution of the Enantiomers of PPDH-OPe

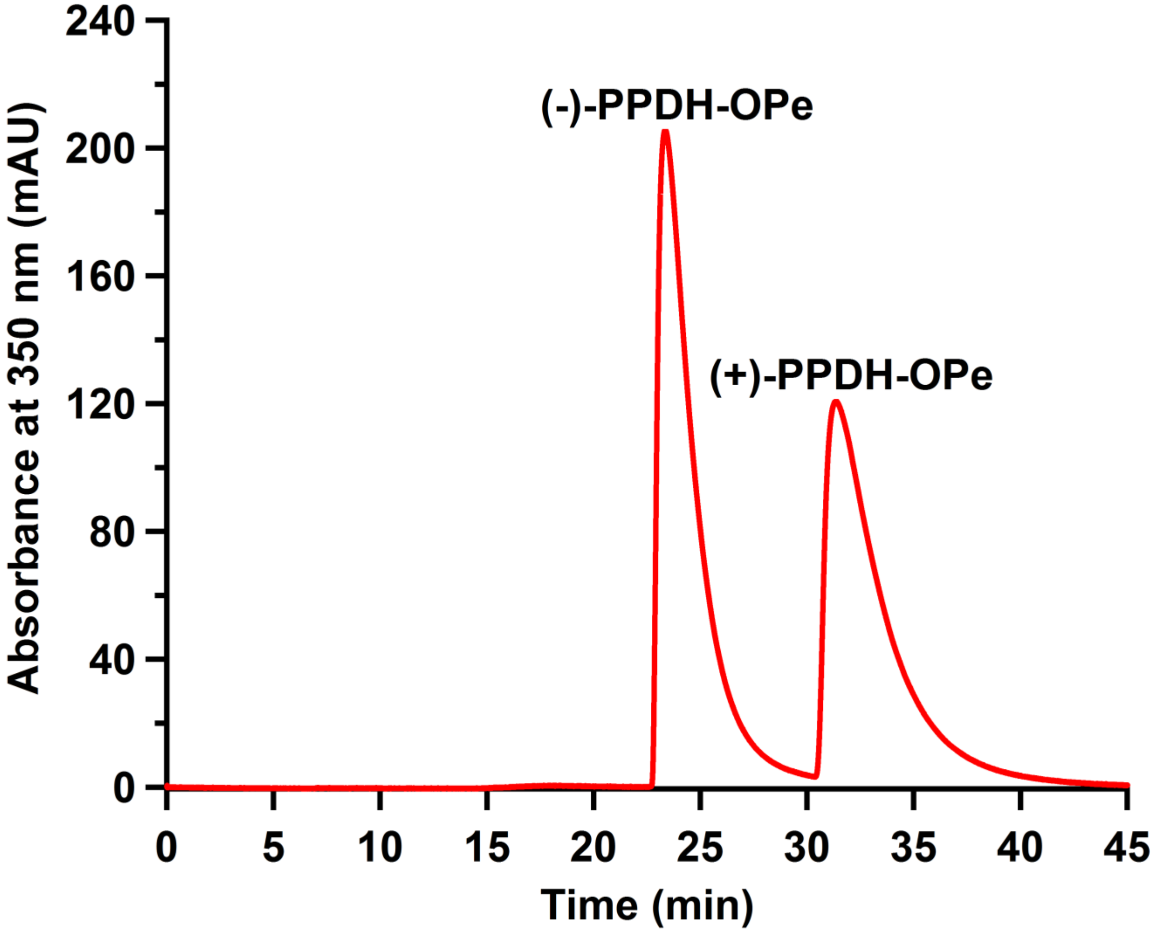

Figure S10. Resolution of the enantiomers of **PPDH-OPe** by chiral preparative HPLC. The enantiomers of **PPDH-OPe** were resolved from 13 mg of racemic material dissolved in 9 mL of 1:8 [v/v] dichloromethane/hexanes. This solution was injected in 1000 µL aliquots onto a CHIRALPAK® IB-3 column (30 mm I.D. × 250 mm, 5 µm), with 16% polar eluent/hexanes (where the polar eluent was a mixture of 99:1 [v/v] dichloromethane/ethyl acetate) flowing at 19 mL/min at room temperature. The (-) and (+) correspond to the sign of the longest-wavelength Cotton effect observed for these enantiomers ($\Delta\varepsilon_{555\ nm}$ = -68 and +65 M^{-1} cm^{-1}).

V. Absorbance and Fluorescence of PPDH, PPDH-OPe, and NPDH in Cyclohexane

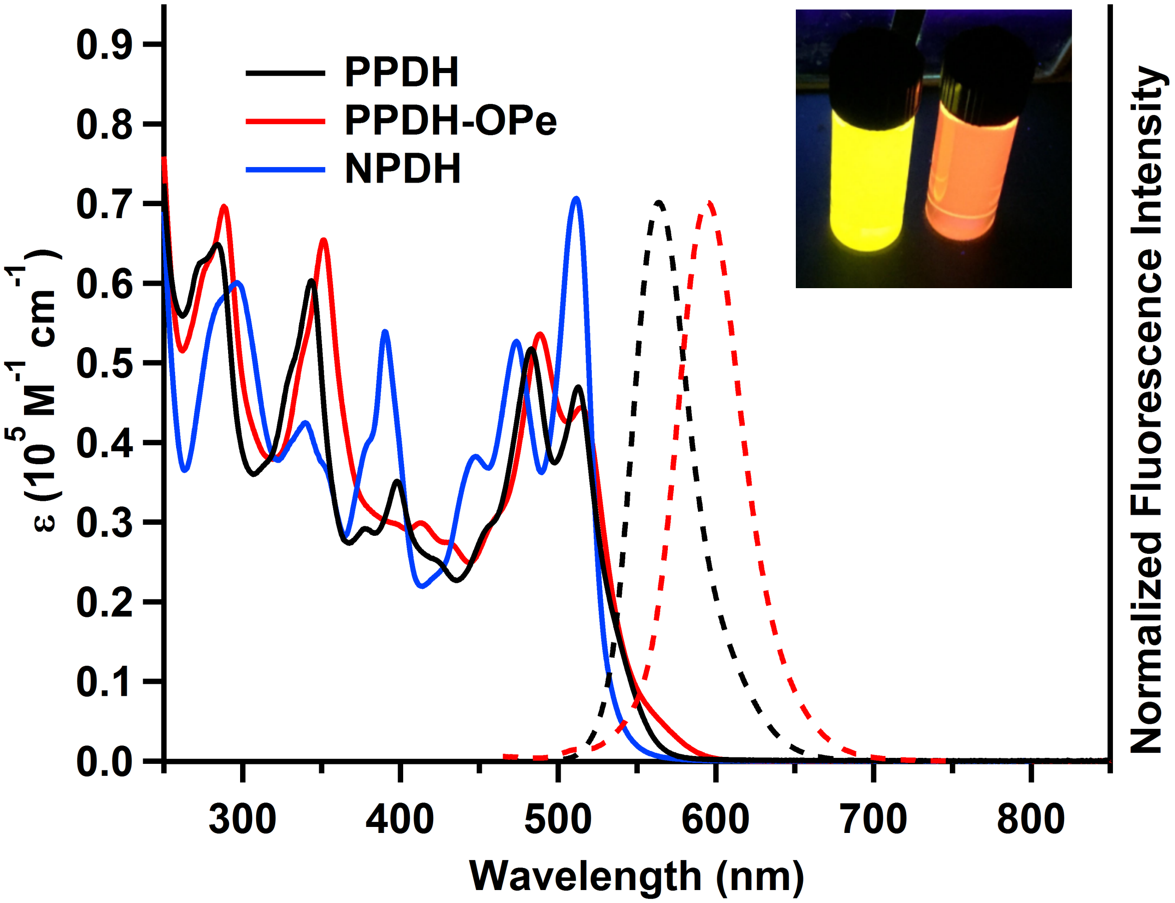

Figure S11. UV-visible absorbance spectra of **PPDH**, **PPDH-OPe**, and **NPDH** in cyclohexane (10 μM, 1 cm path length) and fluorescence spectra of **PPDH** and **PPDH-OPe** in cyclohexane (3 μM, λ_{ex} = 410 nm). Inset shows **PPDH** (left vial) and **PPDH-OPe** (right vial) in cyclohexane under a UV lamp emitting ~254 and ~365 nm light.

VI. ^{1}H-NMR and ^{13}C-NMR Spectra

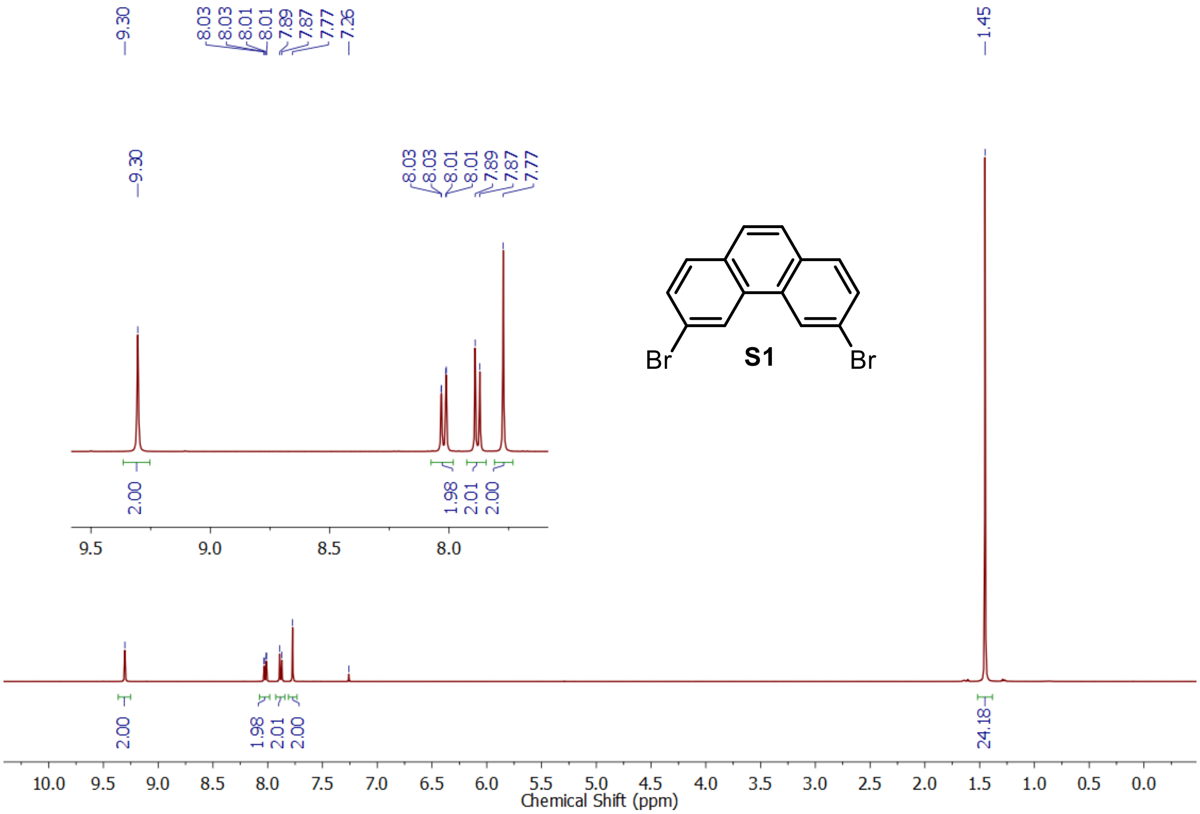

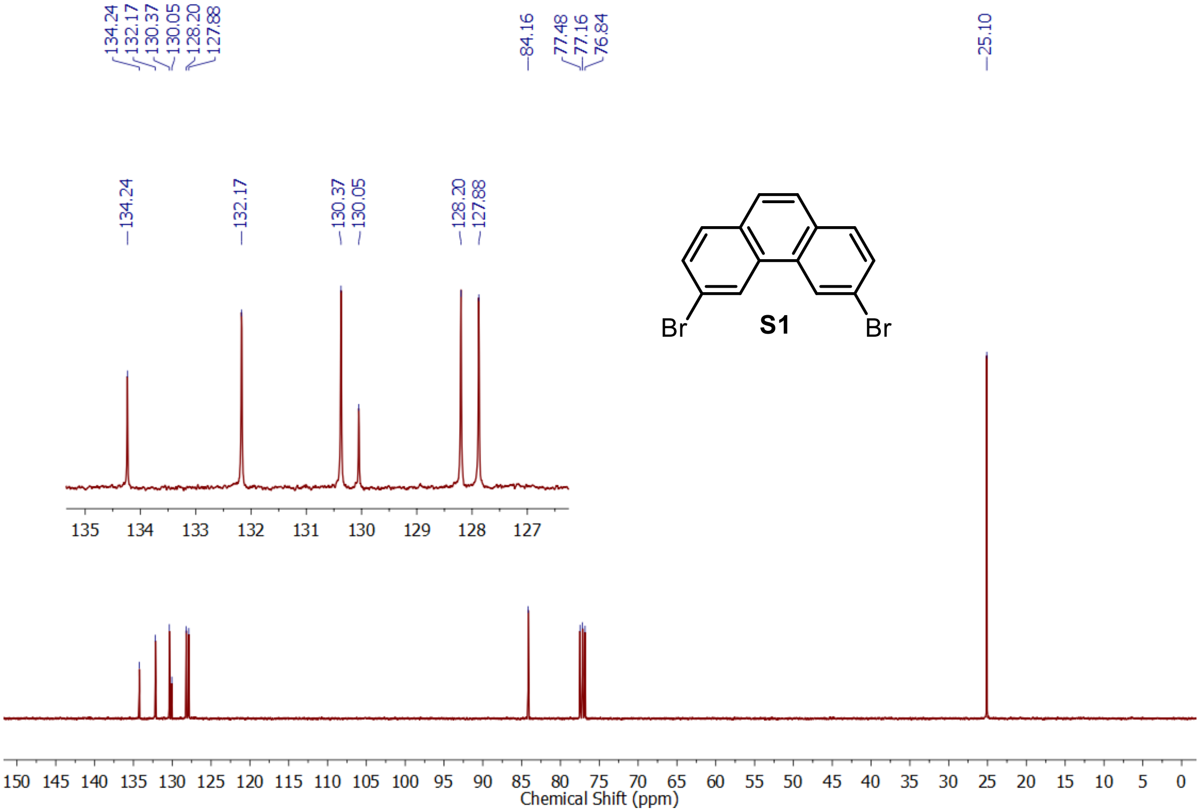

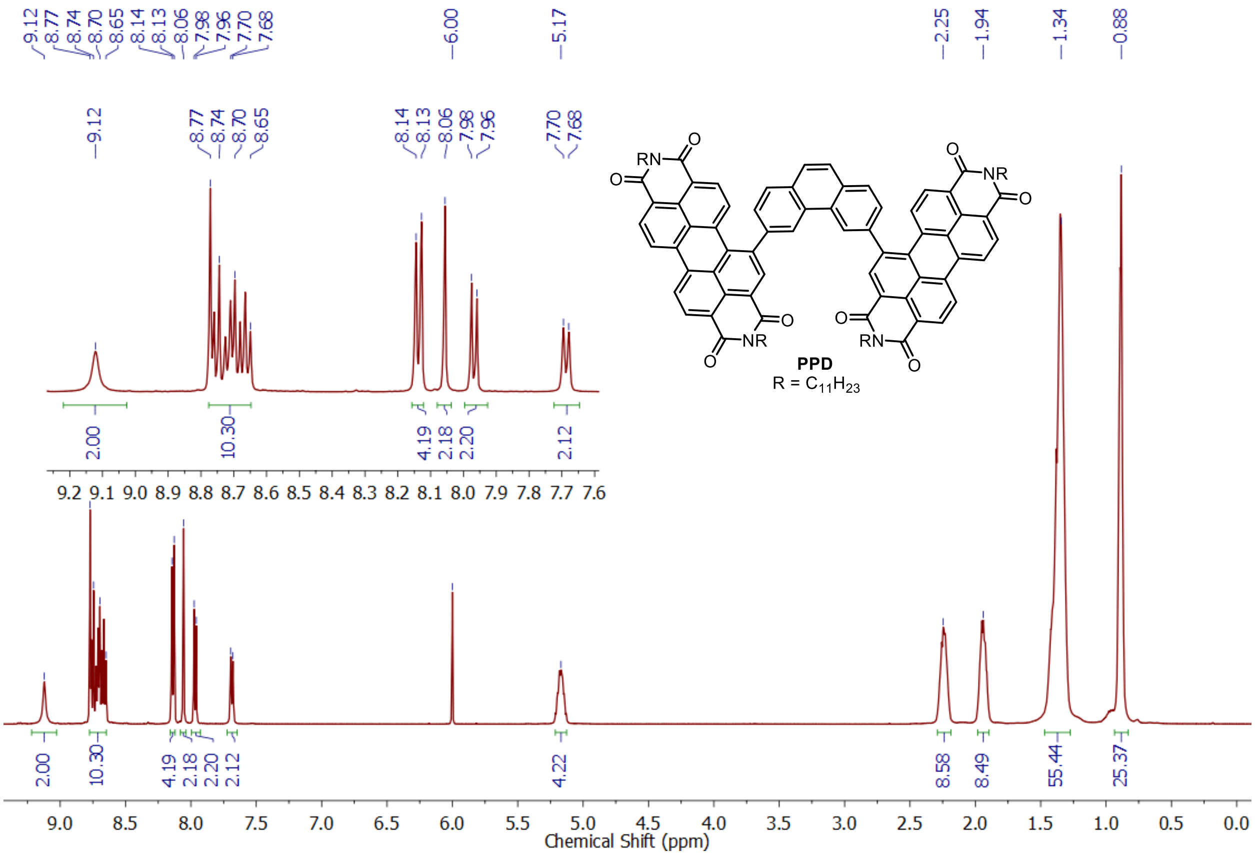
9.12 8.77 8.74 8.70 8.65 8.14 8.13 8.06 7.98 7.96 7.70 7.68
6.00
5.17
2.25
1.94
1.34
0.88
2.00 10.30 4.19 2.18 2.20 2.12
4.22
8.58 8.49 55.44 25.37
PPD
R = C11H23
Chemical Shift (ppm)

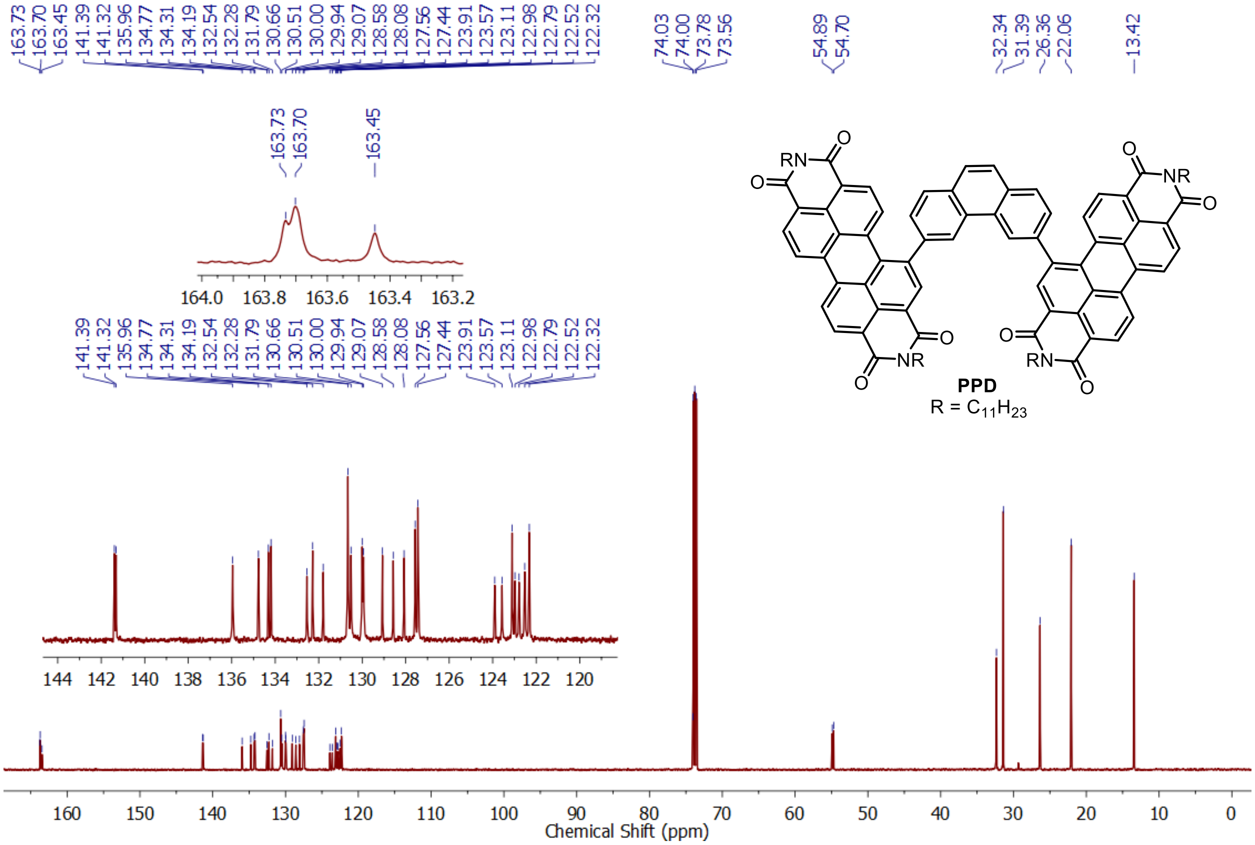
163.73 163.70 163.45 141.39 141.32 135.96 134.77 134.31 134.19 132.54 132.28 131.79 130.66 130.51 130.00 129.94 129.07 128.58 128.08 127.56 127.44 123.91 123.57 123.11 122.98 122.79 122.52 122.32
74.03 74.00 73.78 73.56
54.89 54.70
32.34 31.39 26.36 22.06
13.42
PPD
R = C11H23
Chemical Shift (ppm)

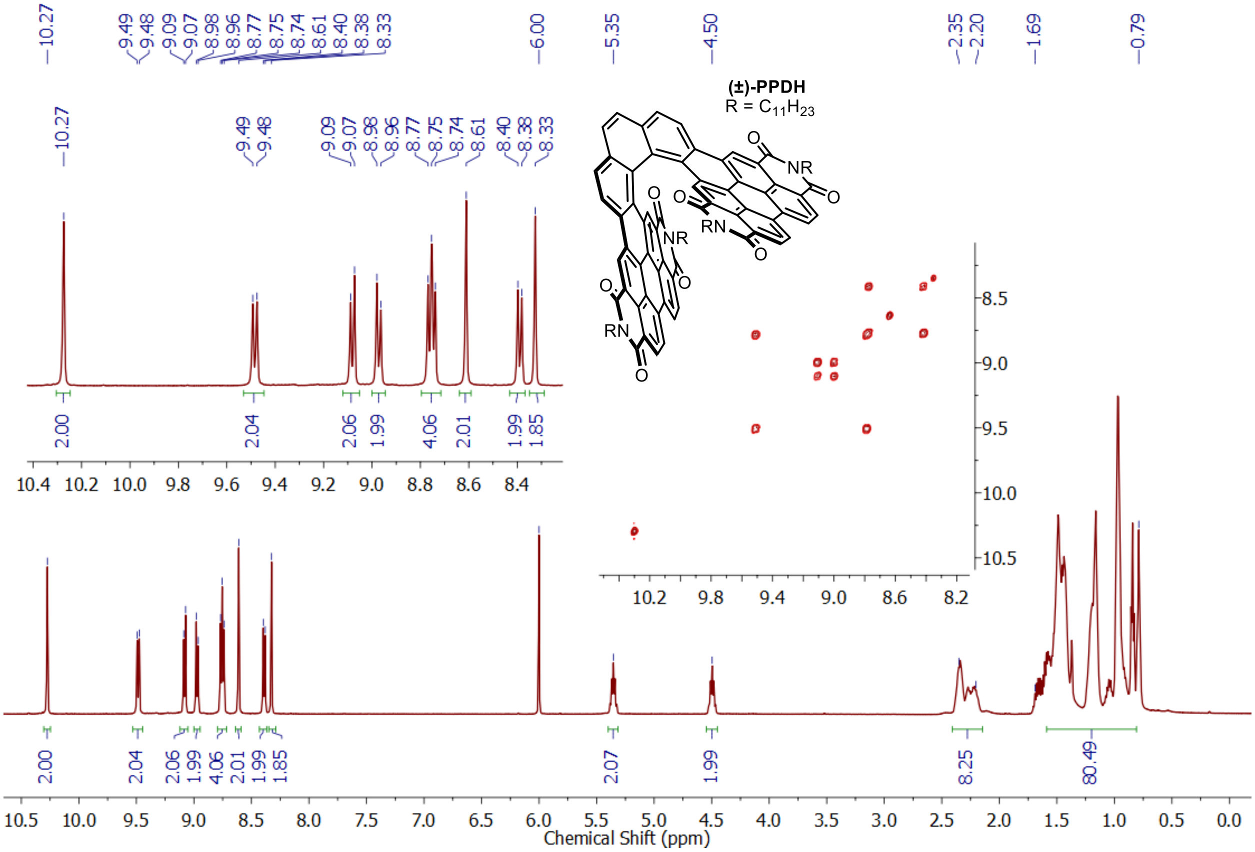
(±)-PPDH
R = C11H23
Chemical Shift (ppm)

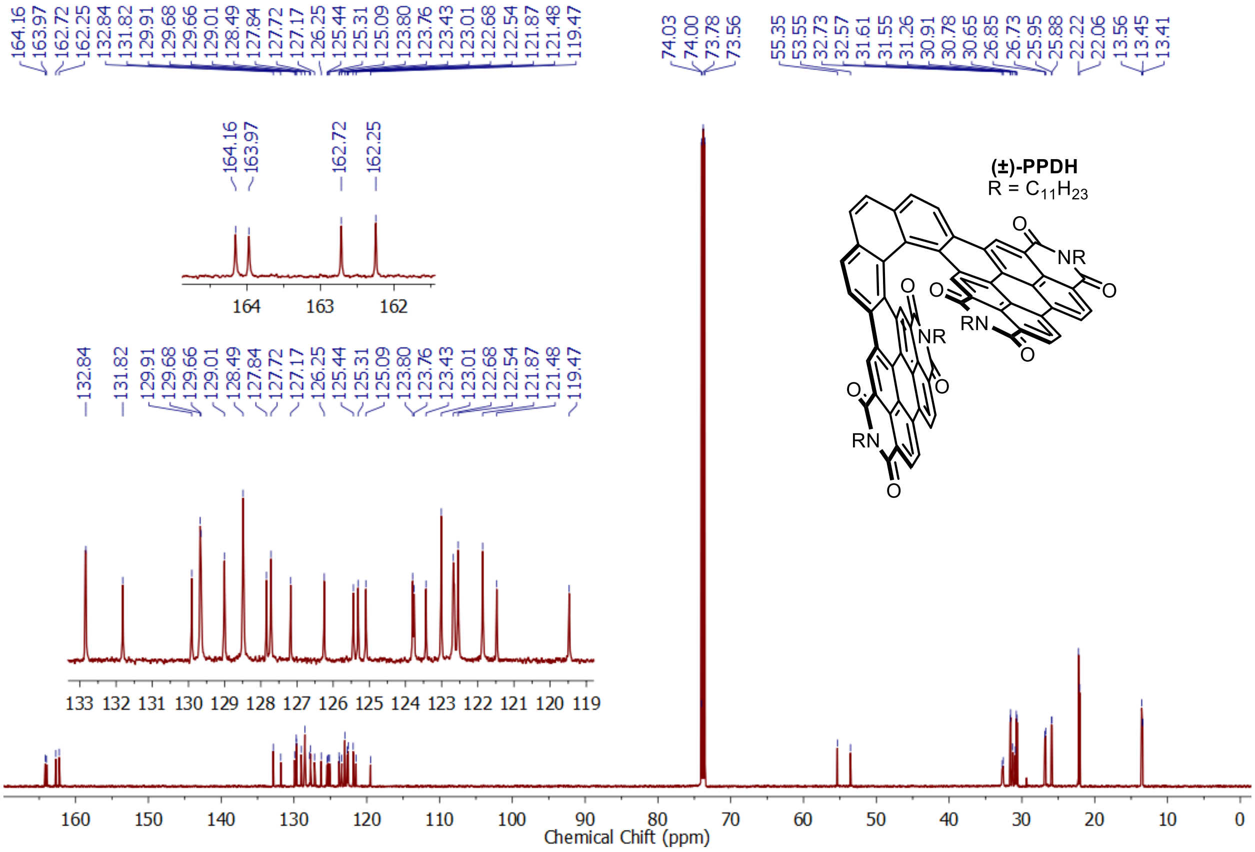
(±)-PPDH
R = C11H23
Chemical Chift (ppm)

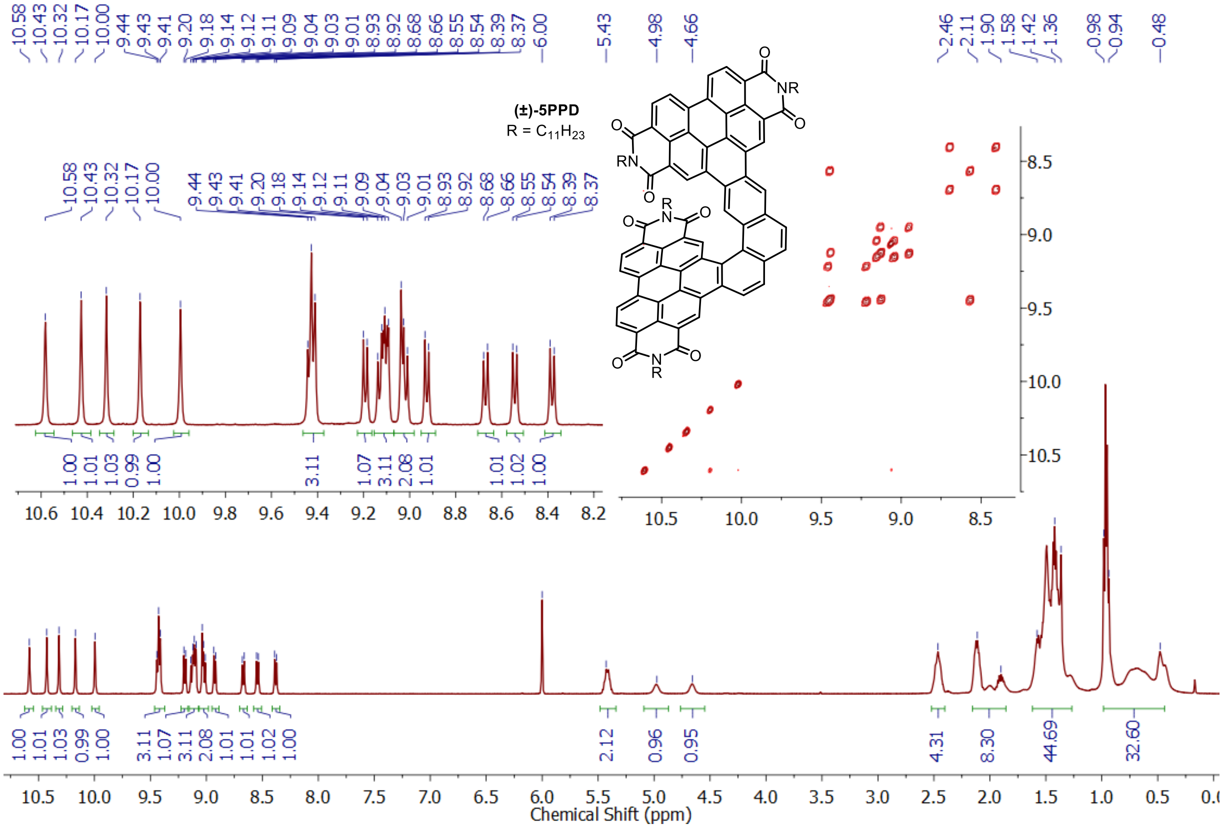
(±)-5PPD
R = C11H23
Chemical Shift (ppm)

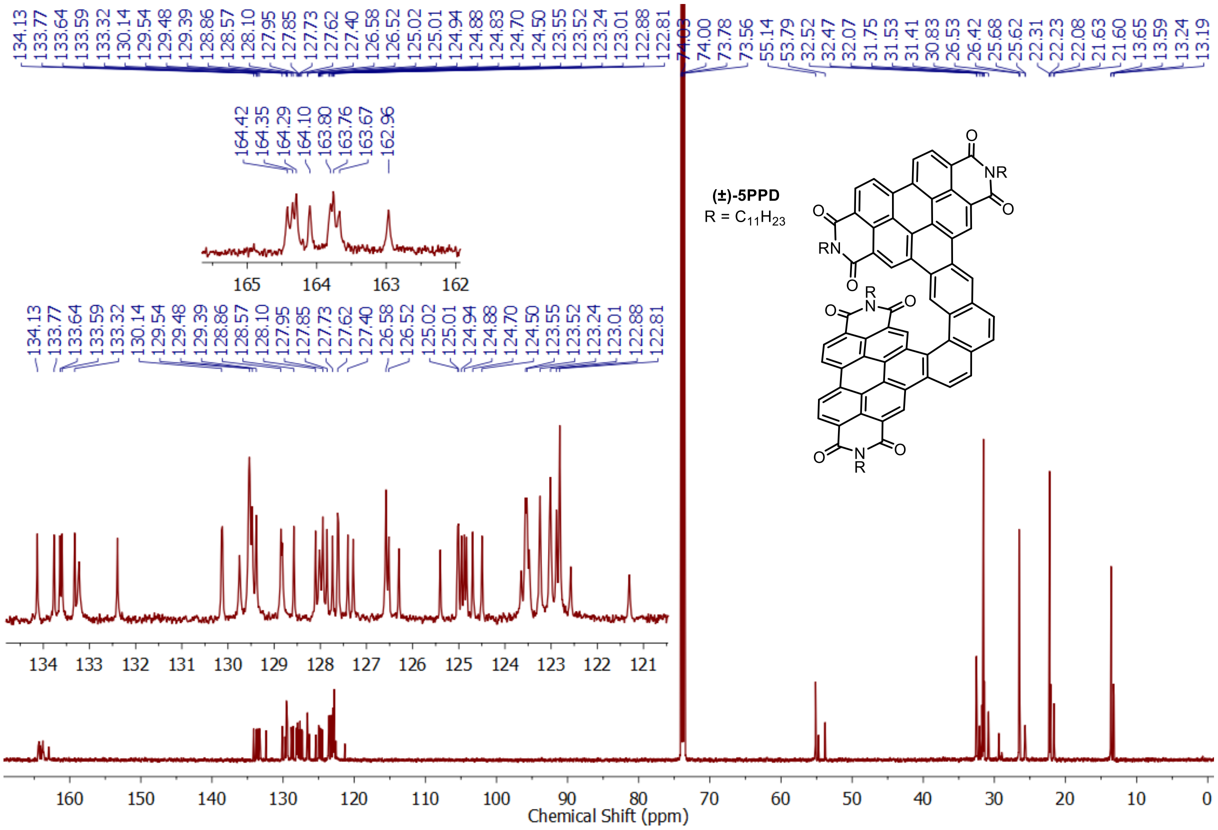
(±)-5PPD
R = C11H23
Chemical Shift (ppm)

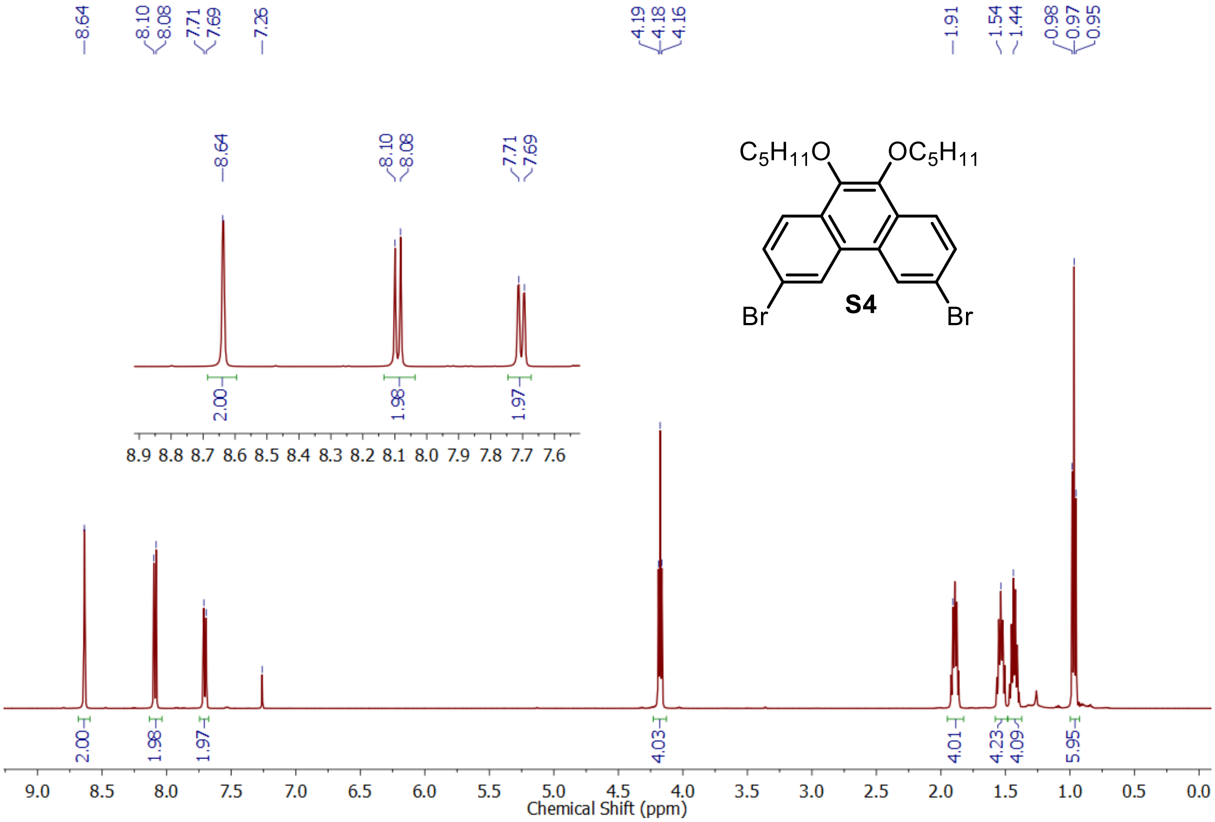

8.64
8.10
8.08
7.71
7.69
7.26
4.19
4.18
4.16
1.91
1.54
1.44
0.98
0.97
0.95
C5H11O
OC5H11
Br
S4
Br
2.00
1.98
1.97
4.03
4.01
4.23
4.09
5.95
Chemical Shift (ppm)

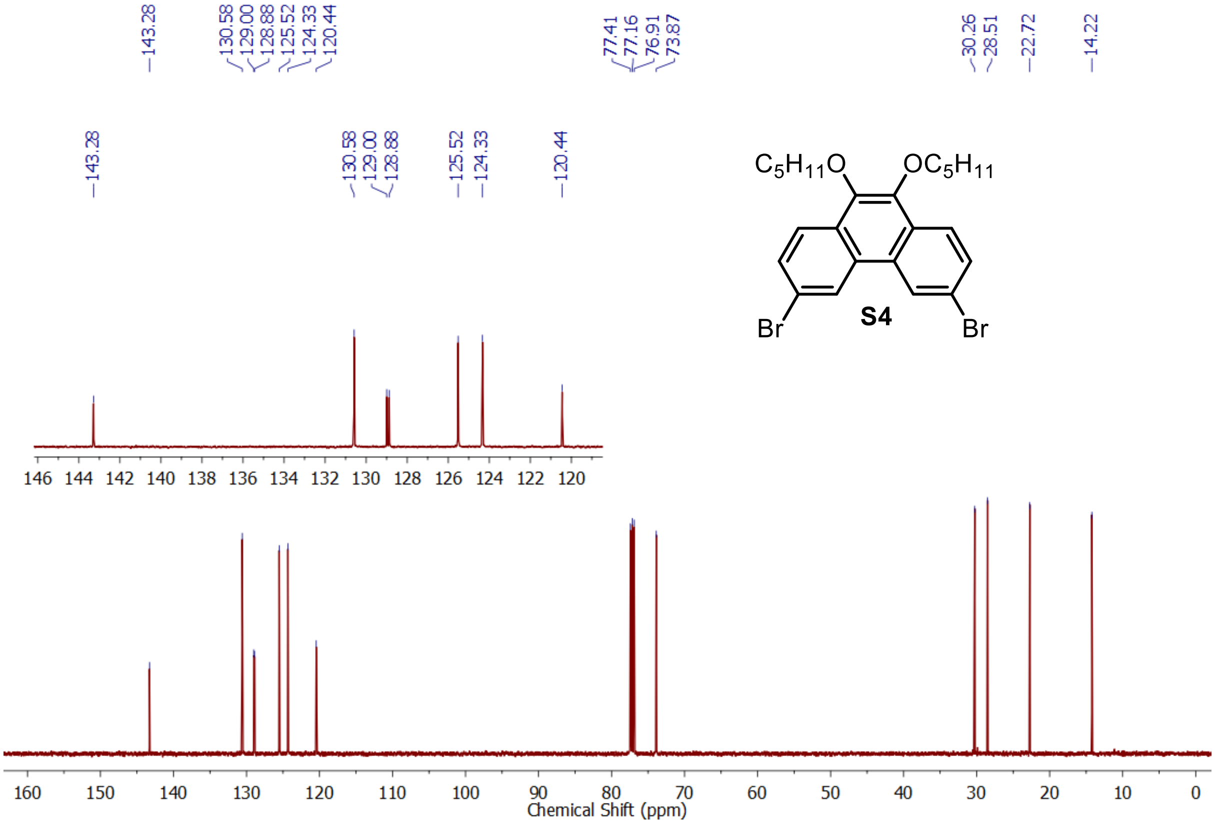

143.28
130.58
129.00
128.88
125.52
124.33
120.44
77.41
77.16
76.91
73.87
30.26
28.51
22.72
14.22
C5H11O
OC5H11
Br
S4
Br
Chemical Shift (ppm)

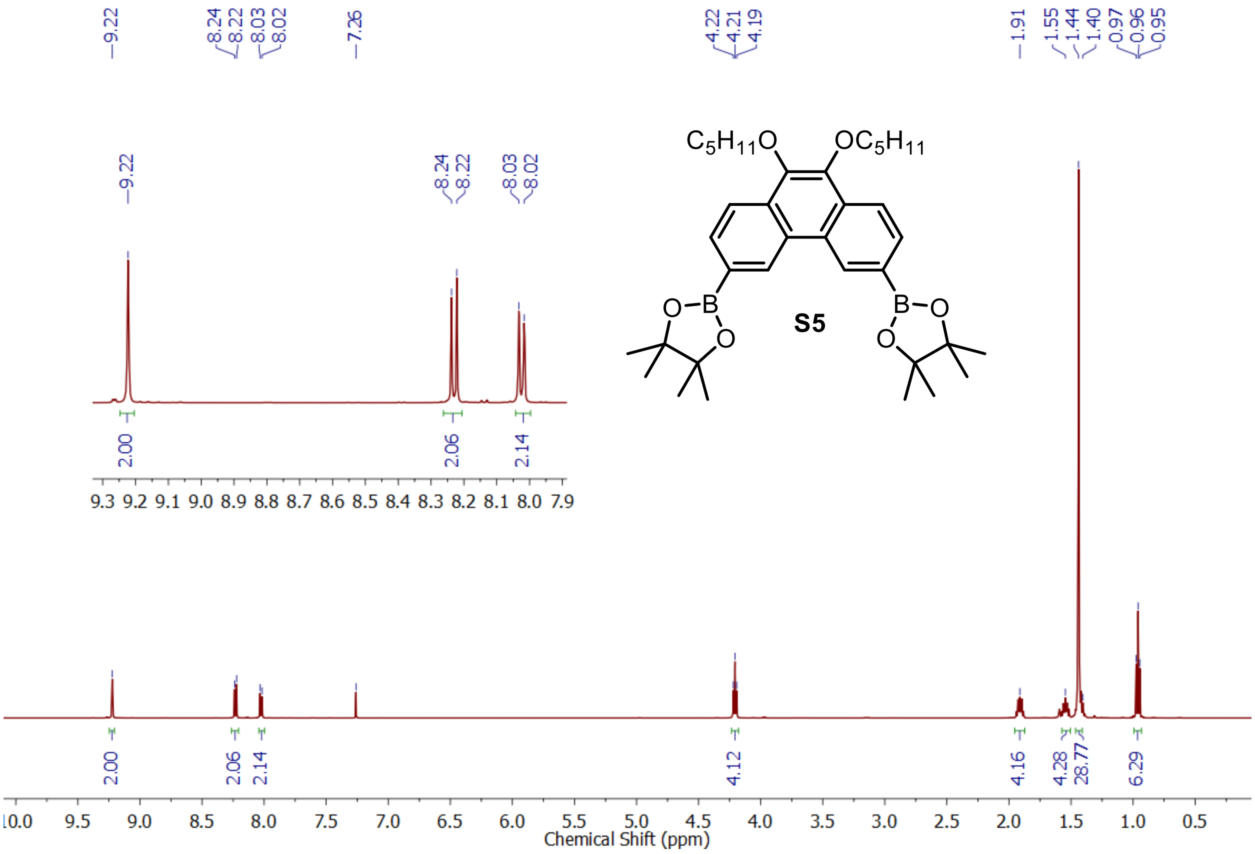

9.22
8.24
8.22
8.03
8.02
7.26
4.22
4.21
4.19
1.91
1.55
1.44
1.40
0.97
0.96
0.95
$C_5H_{11}O$
OC_5H_{11}
S5
2.00
2.06
2.14
4.12
4.16
4.28
28.77
6.29
Chemical Shift (ppm)

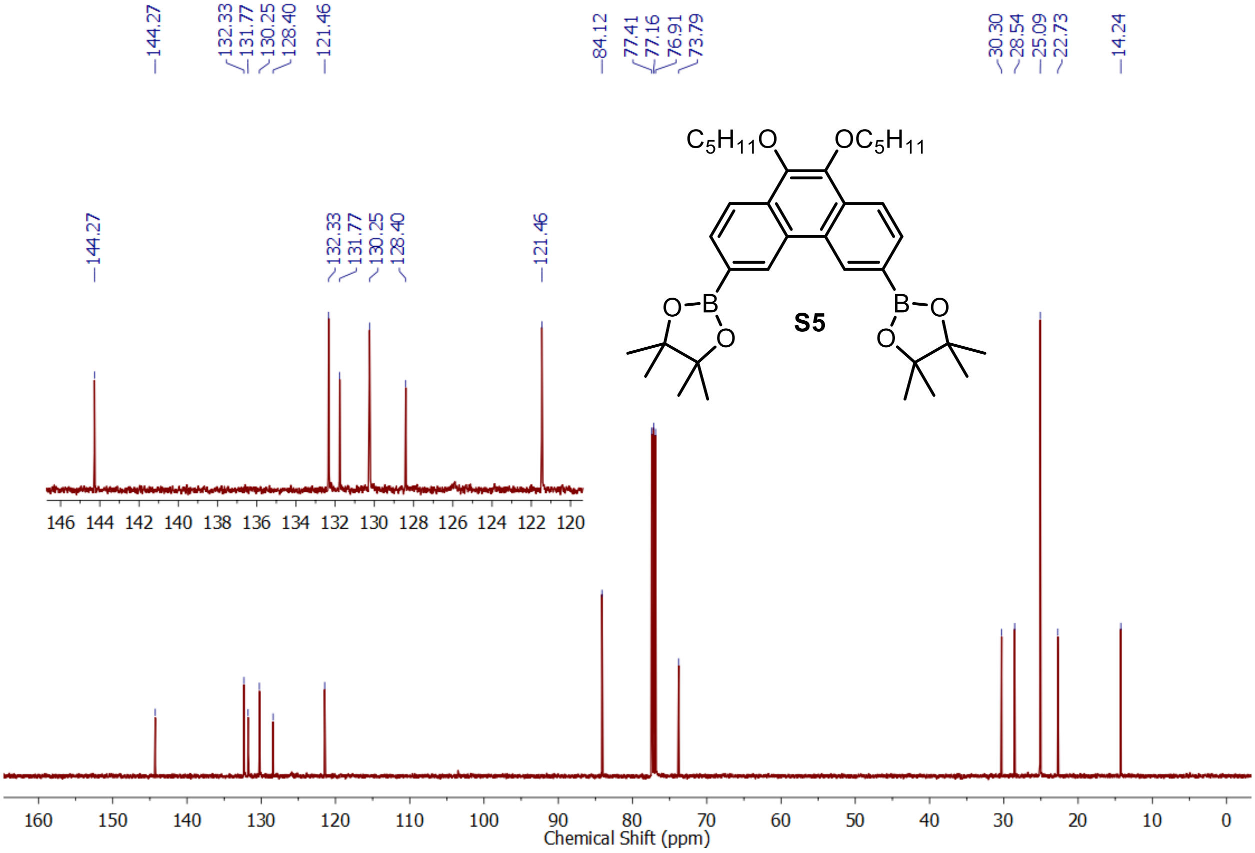

144.27
132.33
131.77
130.25
128.40
121.46
84.12
77.41
77.16
76.91
73.79
30.30
28.54
25.09
22.73
14.24
$C_5H_{11}O$
OC_5H_{11}
S5
Chemical Shift (ppm)

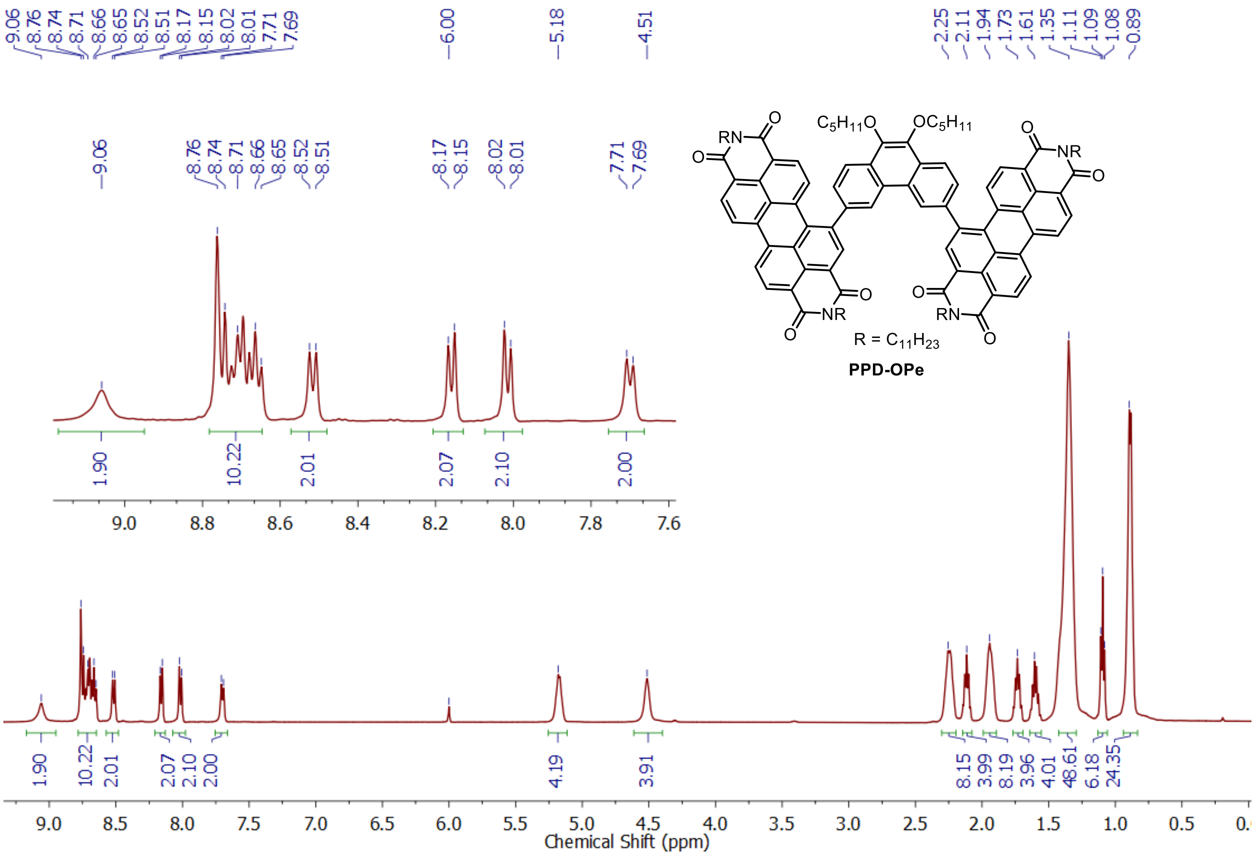

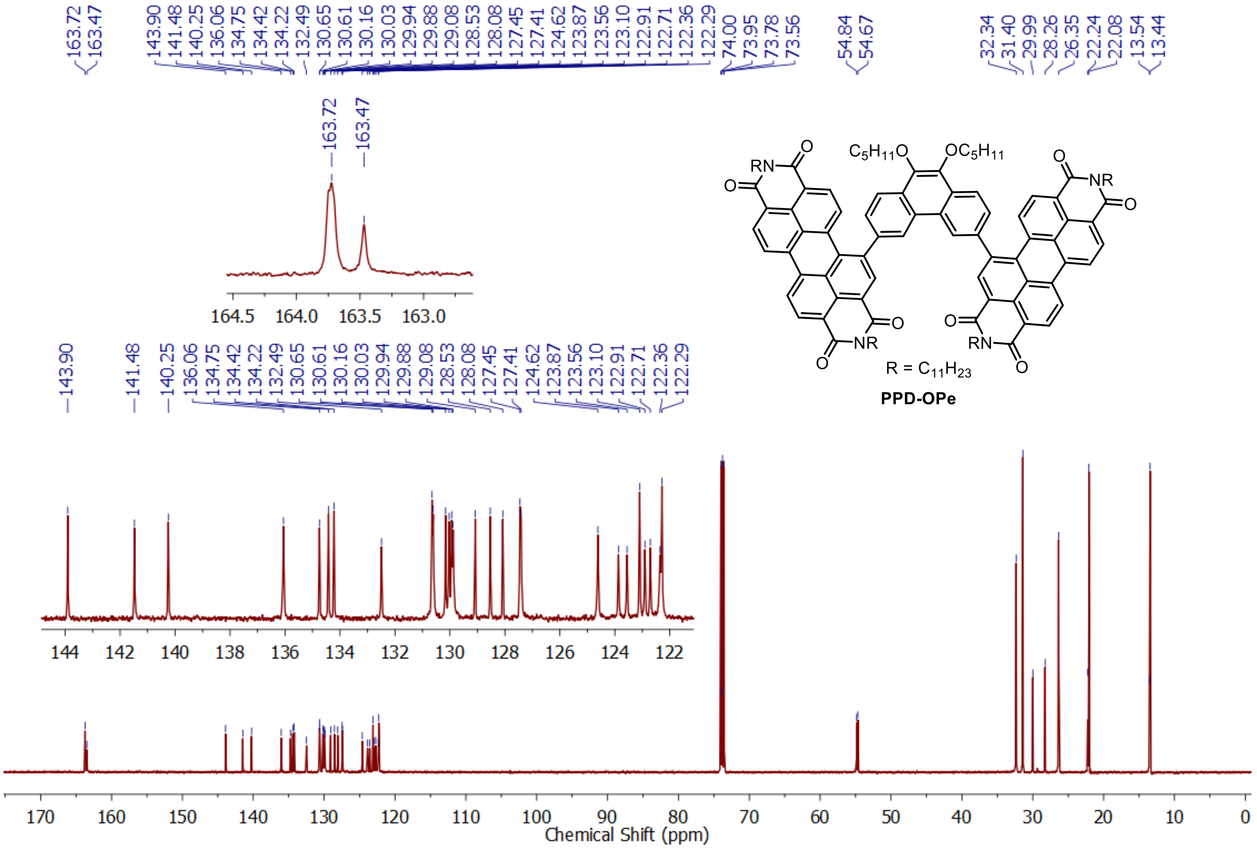

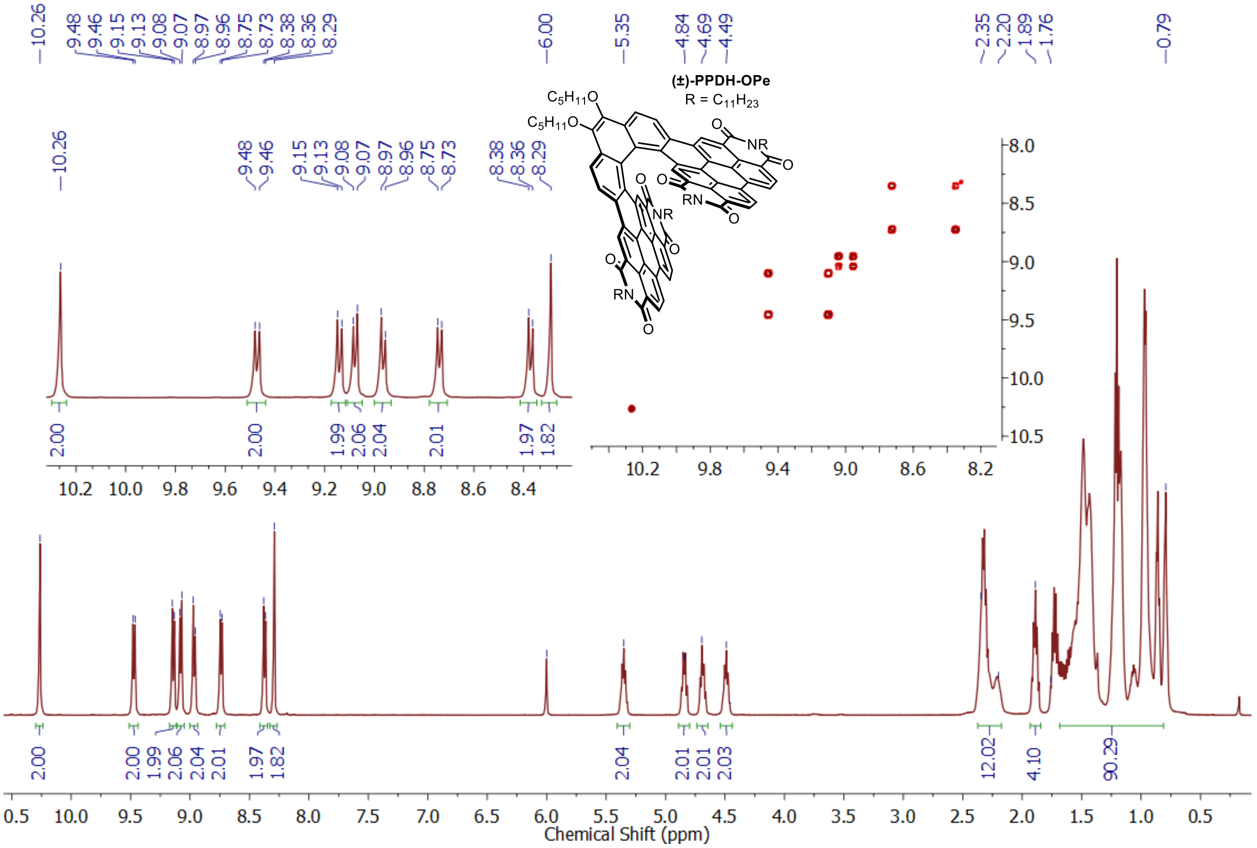
(±)-PPDH-OPe
R = C11H23
Chemical Shift (ppm)

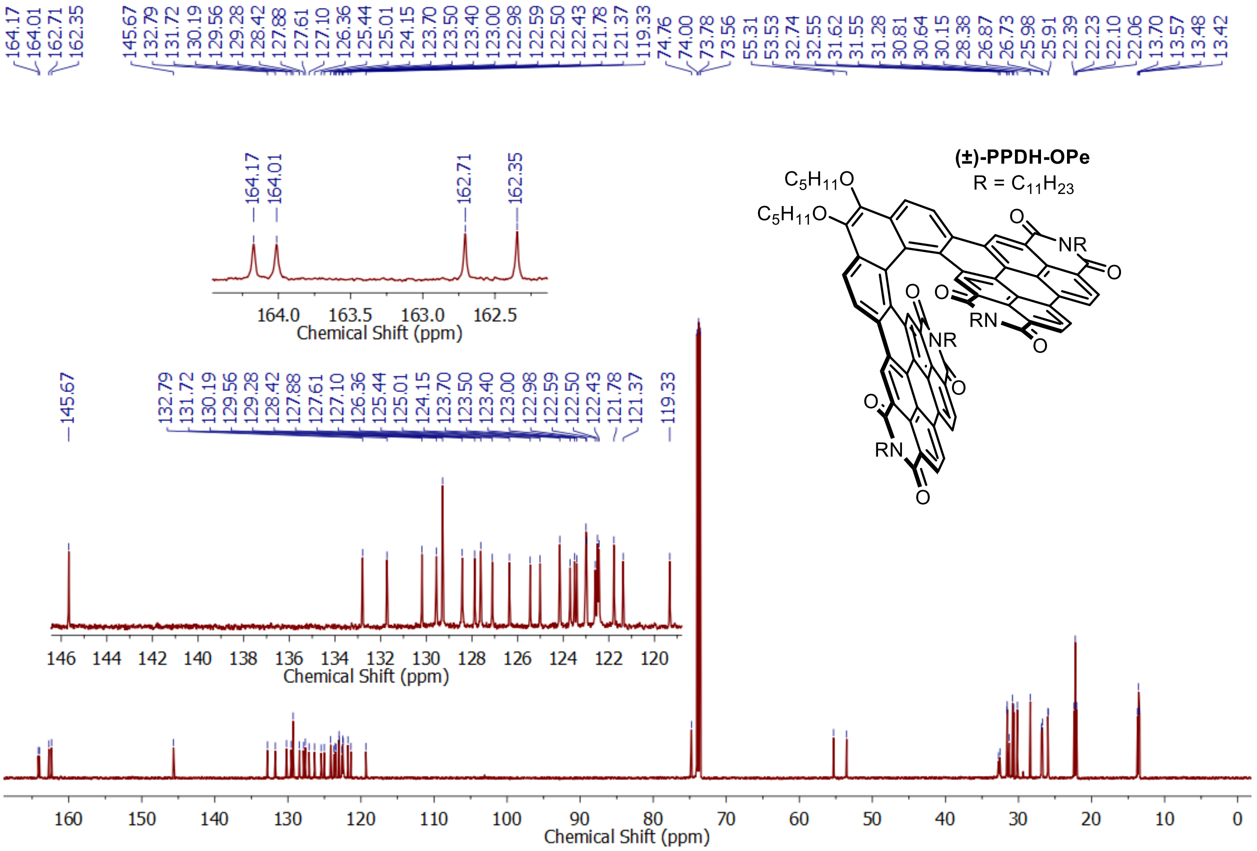
(±)-PPDH-OPe
R = C11H23
Chemical Shift (ppm)

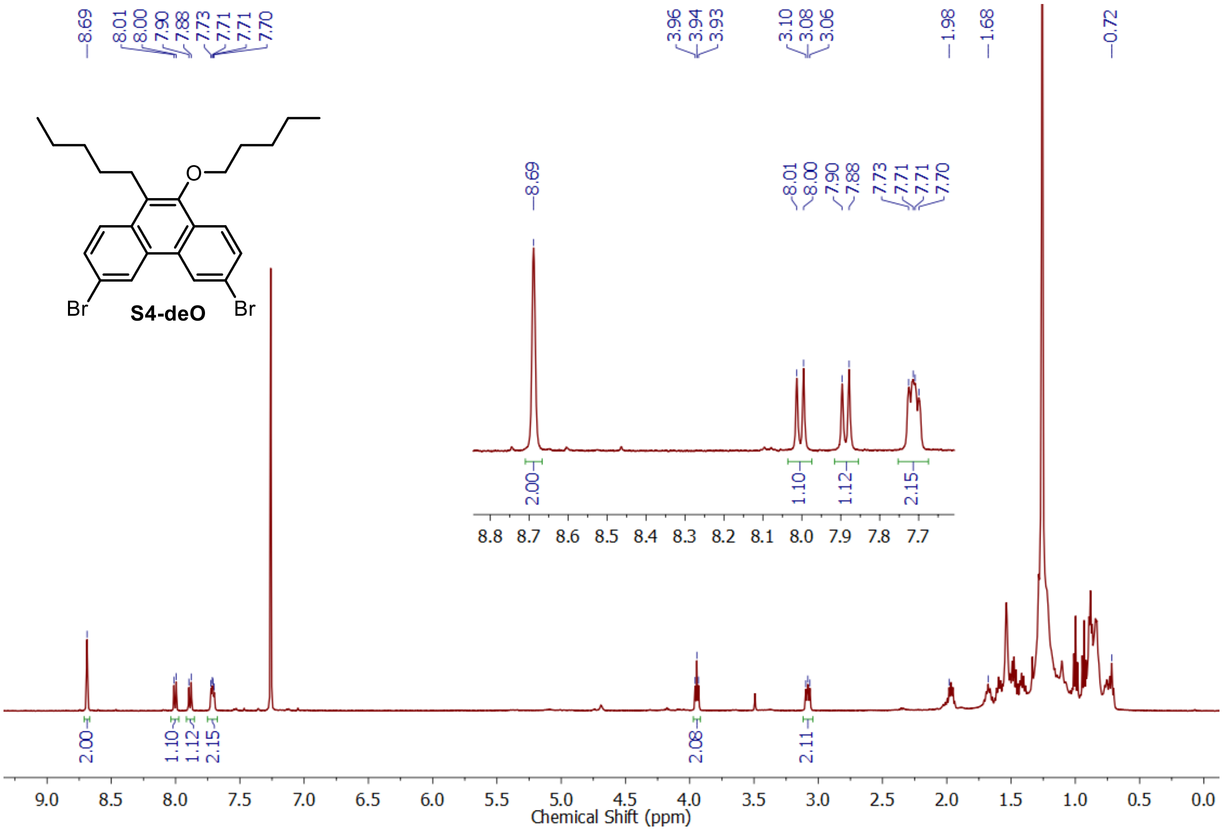
S4-deO
Br
Br
Chemical Shift (ppm)

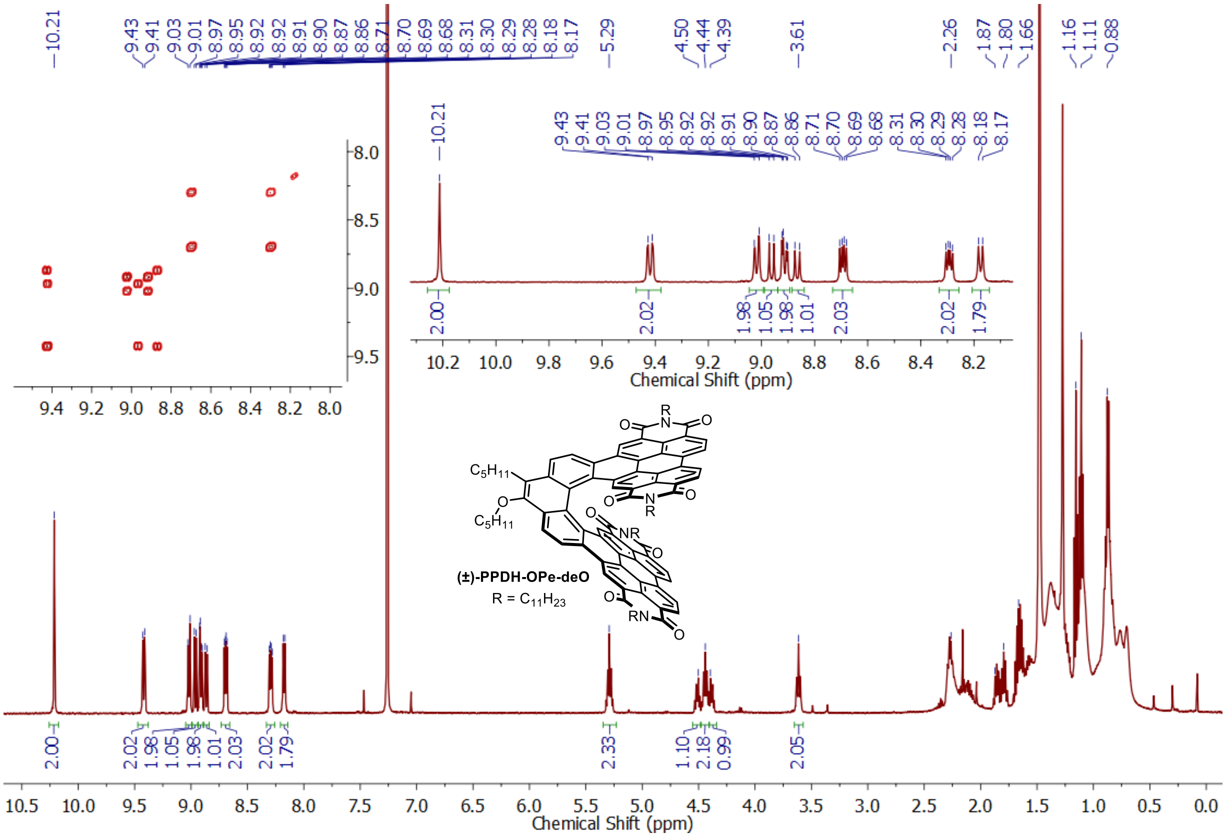
(±)-PPDH-OPe-deO
R = $C_{11}H_{23}$
Chemical Shift (ppm)

VII. DFT-Optimized Molecular Structures and TD-DFT Excited State Calculations

Part A: Calculating Strain Energy

We minimized the geometries of **PPDH**, **5PPD**, and **PPPD** by DFT at the B3LYP/6-31G** level of theory. We calculated the strain energy of the helicenes by the formula:

$$E_{\boldsymbol{PPDH}\ or\ \boldsymbol{5PPD}} - E_{\boldsymbol{PPPD}} = Strain\ Energy \quad (1)$$

The total energies (in hartrees) of the six phenanthrene-bridged PDI-dimers are provided on pages 34, 43, 46, 50, 58, and 61. As an isomer of **PPDH** and **5PPD**, **PPPD** is a good reference because it is nearly planar and, therefore, virtually unstrained. The $CH(C_5H_{11})_2$ and C_5H_{11} chains were modeled as methyl groups to simplify the calculations.

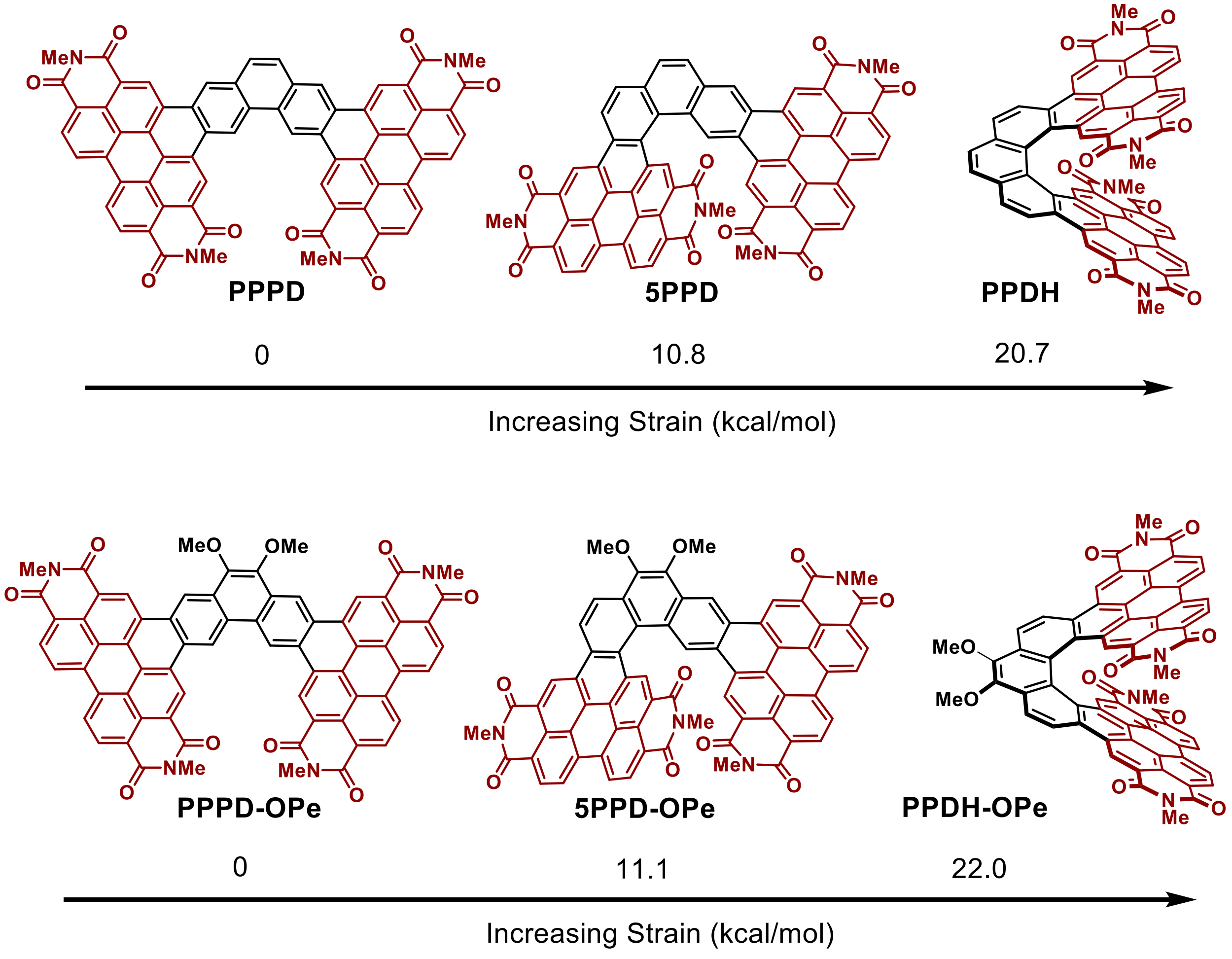

Figure S12. The strain energies of **PPDH**, **PPDH-OPe**, and their isomers calculated using Equation 1.

Part B: Molecular Orbitals, DFT-Optimized Molecular Structure, and TD-DFT Excited State Calculations of PPDH

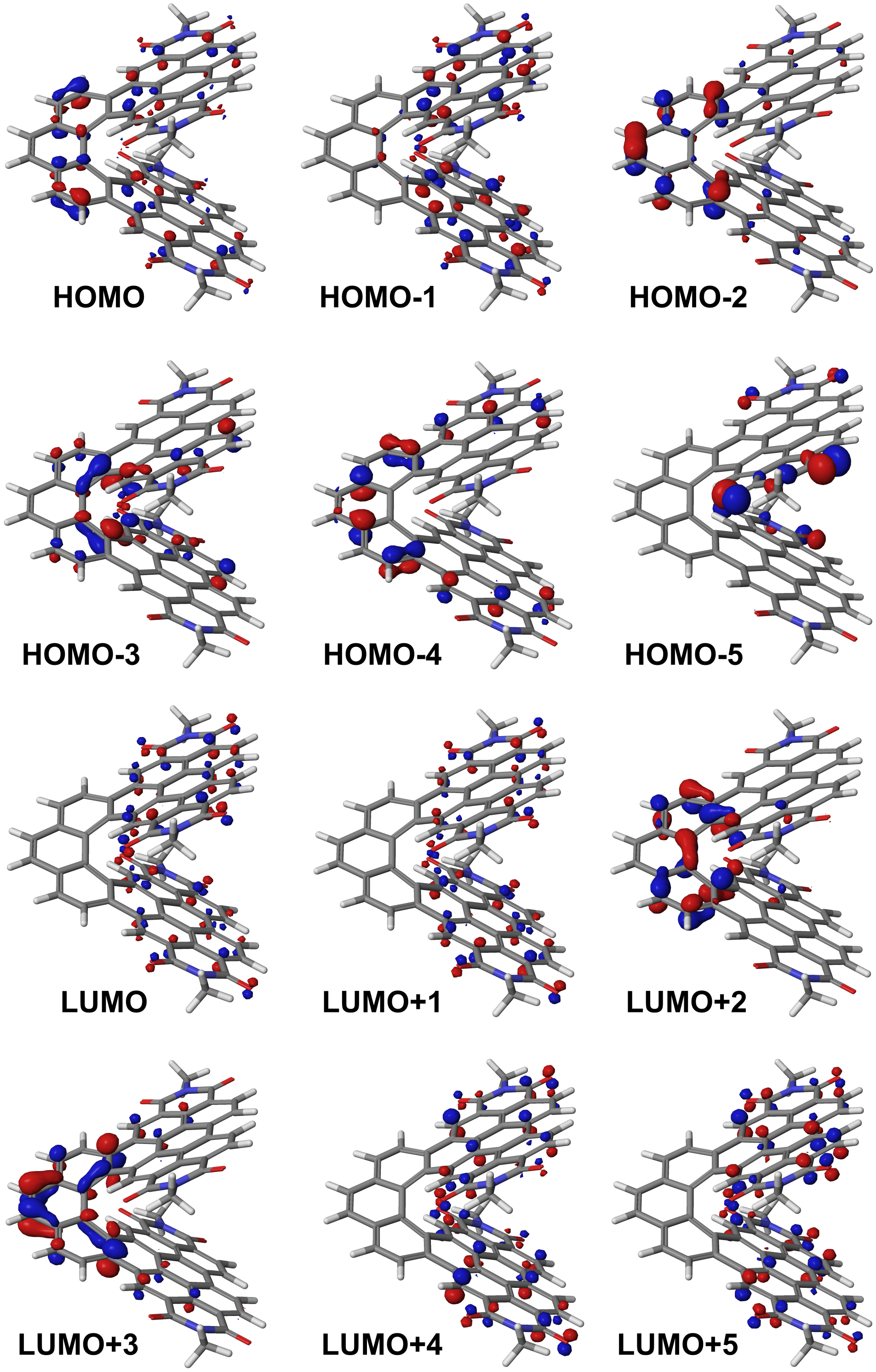

Figure S13. Highest- and lowest-unoccupied molecular orbitals of **PPDH** from DFT (B3LYP/6-31G**). Orbital isosurfaces are illustrated at 0.05 electrons Bohr^{-3}. Methyl groups are used in place of $CH(C_5H_{11})_2$ chains to simplify the calculations.

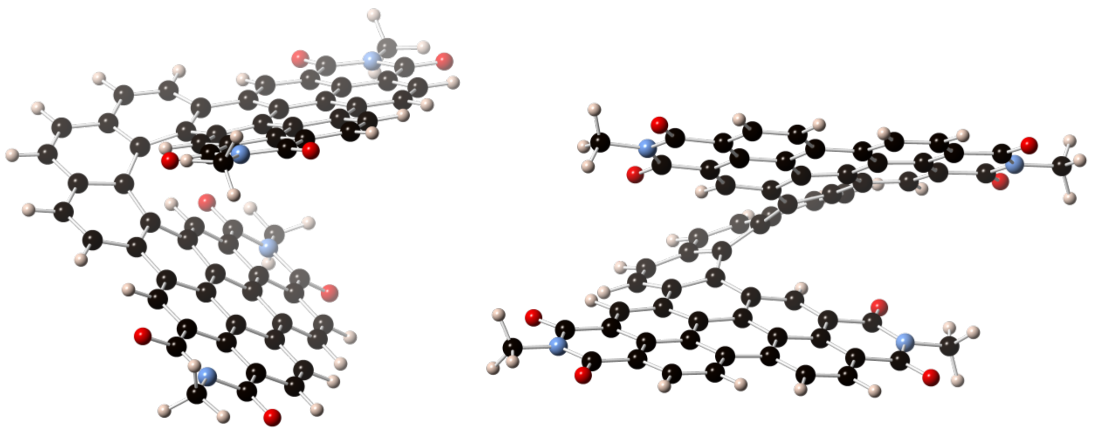

Figure S14. DFT-optimized model of *M*-**PPDH** (B3LYP/6-31G**) from different perspectives. Methyl groups substitute for the $CH(C_5H_{11})_2$ chains to simplify the calculation.

Total energy = -3354.306915 hartrees

atom	x	y	z
C1	-2.1825497627	2.3646560901	0.5642590288
C2	-2.3311990342	0.9473396360	0.6142325285
C3	-3.6326258133	0.3828062069	0.7173625847
C4	-4.7698597722	1.2114987737	0.7198552691
C5	-4.6130520846	2.5914442904	0.6393076515
C6	-3.3411544063	3.1562672986	0.5694617036
C7	-1.1944491725	0.0789899441	0.5685160729
C8	-1.3572951154	-1.3182444480	0.7087202744
C9	-2.6726642468	-1.8443531697	0.8362093940
C10	-3.7762523313	-1.0299459981	0.8200673835
C11	-5.1262558308	-1.6429916531	0.9040079896
N12	-6.2185285434	-0.7690334209	0.8696219768
C13	-6.1345020186	0.6325351371	0.7985908192
C14	-0.8309694905	2.9298789953	0.5413201136
C15	0.2913216198	2.0491575481	0.4755506099
C16	0.1196271254	0.6280881949	0.4300483123
C17	-0.5870339442	4.3106135527	0.6027554415
C18	0.7053809046	4.8298245361	0.6163868064
C19	1.8053602630	3.9804088846	0.5592726976
C20	1.6064187463	2.5907367724	0.4750537014
C21	2.7243552835	1.7098411410	0.4306898059
C22	2.5379788605	0.3551211655	0.3357614853
C23	1.2390650937	-0.2300358563	0.2842089163
C24	3.1756387790	4.5436994595	0.6178125216
N25	4.2405298814	3.6298581940	0.6137277191
C26	4.1124669790	2.2378878000	0.4991329208
C27	-7.5446775066	-1.3922604035	0.9305253111
C28	5.5875267511	4.2022707797	0.7071227562
O29	3.3978486391	5.7456100477	0.6814394228

O30	5.0967158642	1.5102796891	0.4568807883
O31	-5.3061262985	-2.8507213894	0.9944672970
O32	-7.1408417845	1.3283745218	0.7962132003
C33	1.0504631032	-1.6688510699	0.2542451371
C34	-0.2016192432	-2.1870125774	0.6688503614
C35	2.1147882900	-2.6084352431	-0.0459346356
C36	2.0605833275	-3.8837590586	0.5795990285
C37	0.8290756377	-4.3322150309	1.1359253988
C38	-0.2874640679	-3.5508115651	1.0824896308
C39	3.2370478836	-2.3395369834	-0.9190595117
C40	4.4367481583	-3.0658890156	-0.6834382360
C41	4.4095939755	-4.2427410480	0.1181839858
C42	3.2316062280	-4.6925557819	0.6436855706
C43	3.2216699067	-1.4149314003	-2.0394681101
C44	4.4562454166	-0.8842839981	-2.4888289404
C45	5.6730919082	-1.4911766621	-2.0512490310
C46	5.6527944926	-2.6044301366	-1.2629863170
H47	-5.5012172675	3.2134590926	0.6378786769
H48	-3.2612557279	4.2353351397	0.5187355932
H49	-2.8461623198	-2.9105468656	0.9007977274
H50	-1.4172533864	5.0042253146	0.6591381071
H51	0.8782319326	5.8983715752	0.6822286368
H52	3.4218322258	-0.2646038930	0.3497466782
H53	-8.2869805505	-0.6033075839	0.8410510333
H54	-7.6464833186	-2.1150326982	0.1186354263
H55	-7.6639547653	-1.9245922737	1.8769948257
H56	5.6233361272	4.9050438804	1.5404170353
H57	6.2877954596	3.3838877751	0.8534219337
H58	5.8332390211	4.7448190481	-0.2092152517
H59	0.7845201090	-5.3279303726	1.5675264899
H60	-1.2285099785	-3.9451460266	1.4441862417
H61	5.3309123218	-4.8019782664	0.2534809292
H62	3.1794213551	-5.6452349990	1.1625791361
H63	6.6212020488	-1.1229893970	-2.4209816134
H64	6.5723791109	-3.1355276428	-1.0352954500
C65	3.2604164895	0.6383396320	-4.0354250978
C66	4.4637141670	0.2266630758	-3.4169170019
C67	5.6556159657	0.9518928244	-3.6939510218
C68	5.6588053411	2.0381135448	-4.5311633214
H69	6.5928043919	0.6952183933	-3.2183904005
C70	4.4630536306	2.4552435848	-5.1813350566
C71	3.2566033430	1.7375126800	-4.9524736186
C72	2.0699493214	2.1201195096	-5.6458725487
C73	2.1264883846	3.2380560477	-6.4924974214
C74	3.3058578959	3.9542506567	-6.6845119758
C75	4.4784016110	3.5668686351	-6.0445753467

H76	1.2390505982	3.5649090161	-7.0193472247
H77	3.3348599879	4.8179725819	-7.3395115298
C78	2.0509074959	-0.0831934301	-3.7825561976
C79	2.0285352735	-1.1150846980	-2.8099935989
C80	0.8610205602	-1.9295666437	-2.7320309875
C81	-0.2535818755	-1.6804271253	-3.4917943635
H82	0.8405889980	-2.8051399604	-2.1012456209
C83	-0.2834048605	-0.5796254125	-4.3950834220
C84	0.8785826176	0.2259118190	-4.5473816644
C85	0.8599097606	1.3143768338	-5.4708942969
C86	-0.3184468351	1.5515863735	-6.1963723828
C87	-1.4452481133	0.7466147772	-6.0502693403
C88	-1.4355802785	-0.3215442761	-5.1594534320
H89	-0.3610942013	2.3668230175	-6.9072202725
H90	-2.3419002007	0.9283945762	-6.6323847345
C91	6.9293448876	2.7765155213	-4.7506367231
N92	6.8769933132	3.8812449870	-5.6089106918
C93	5.7266086537	4.3357428133	-6.2772756058
C94	8.1371532357	4.6053593265	-5.8068486642
O95	5.7638582965	5.3146652177	-7.0106267156
O96	7.9860424153	2.4579116491	-4.2211394241
C97	-2.6259317065	-1.1974838740	-5.0466079035
N98	-2.5408127169	-2.2750477317	-4.1523704107
C99	-1.4243602201	-2.5860440902	-3.3623605423
C100	-3.7227296647	-3.1386736675	-4.0647913075
O101	-3.6470358155	-1.0187218090	-5.6971455369
O102	-1.4292476141	-3.5473689031	-2.6041389457
H103	7.9449957691	5.4235811799	-6.4961955111
H104	8.4994534768	4.9861273318	-4.8494268788
H105	8.8923837103	3.9284903235	-6.2116497072
H106	-3.5517541522	-3.8561008361	-3.2662802860
H107	-4.6048469060	-2.5304416711	-3.8596050394
H108	-3.8779070215	-3.6573552991	-5.0140533031

PPDH Frontier Molecular Orbital Energies (eV):

LUMO+9	LUMO+8	LUMO+7	LUMO+6	LUMO+5
-1.27349	-1.38669	-1.63296	-1.63813	-1.76983
LUMO+4	LUMO+3	LUMO+2	LUMO+1	LUMO
-1.81963	-2.16793	-2.27134	-3.2602	-3.28605
HOMO	HOMO-1	HOMO-2	HOMO-3	HOMO-4
-5.88855	-6.00528	-6.24093	-6.39033	-6.86163
HOMO-5	HOMO-6	HOMO-7	HOMO-8	HOMO-9
-7.28585	-7.28912	-7.29266	-7.29783	-7.3166

Restricted Singlet Excited State 1:
2.2136 eV 560.11 nm

excitation	X coeff.
HOMO-3 => LUMO	0.13047
HOMO-1 => LUMO+1	-0.36785
HOMO => LUMO	-0.91680

Transition dipole moment (debye):
X= -2.5697 Y= -0.8561 Z= 1.4840
Tot= 3.0884

Oscillator strength, f= 0.0801

Restricted Singlet Excited State 2:
2.2312 eV 555.69 nm

excitation	X coeff.
HOMO-1 => LUMO	-0.37572
HOMO => LUMO+1	-0.91712

Transition dipole moment (debye):
X= -0.5835 Y= 0.8871 Z= -0.3855
Tot= 1.1296

Oscillator strength, f= 0.0108

Restricted Singlet Excited State 3:
2.3857 eV 519.70 nm

excitation	X coeff.
HOMO-3 => LUMO+1	-0.17611
HOMO-2 => LUMO	0.31161
HOMO-1 => LUMO	-0.86374
HOMO => LUMO+1	0.32718

Transition dipole moment (debye):
X= -0.3208 Y= 0.0556 Z= 0.0341
Tot= 0.3274

Oscillator strength, f= 0.0010

Restricted Singlet Excited State 4:
2.4107 eV 514.30 nm

excitation	X coeff.
HOMO-3 => LUMO	0.25271
HOMO-2 => LUMO+1	-0.40311
HOMO-1 => LUMO+1	0.81908
HOMO => LUMO	-0.28766

Transition dipole moment (debye):
X= -4.2084 Y= -2.3244 Z= 0.1421

Tot= 4.8097

Oscillator strength, f= 0.2115

Restricted Singlet Excited State 5:
2.5447 eV 487.23 nm

excitation X coeff.
------------ ---------
HOMO-3 => LUMO -0.36853
HOMO-2 => LUMO+1 -0.85960
HOMO-1 => LUMO+1 -0.29278
HOMO-1 => LUMO+3 0.10909

Transition dipole moment (debye):
X= 3.0354 Y= 0.4303 Z= -2.2792
Tot= 3.8201

Oscillator strength, f= 0.1408

Restricted Singlet Excited State 6:
2.5508 eV 486.06 nm

excitation X coeff.
------------ ---------
HOMO-3 => LUMO+1 -0.14627
HOMO-2 => LUMO -0.90971
HOMO-1 => LUMO -0.26449
HOMO-1 => LUMO+2 0.13558
HOMO => LUMO+1 0.11117
HOMO => LUMO+3 -0.16955

Transition dipole moment (debye):
X= 0.8624 Y= -2.2528 Z= 1.1692
Tot= 2.6806

Oscillator strength, f= 0.0695

Restricted Singlet Excited State 7:
2.7162 eV 456.47 nm

excitation X coeff.
------------ ---------
HOMO-3 => LUMO 0.11614
HOMO-3 => LUMO+1 -0.92730
HOMO-2 => LUMO 0.13272
HOMO-1 => LUMO 0.16736
HOMO-1 => LUMO+2 0.16659
HOMO => LUMO+1 -0.15063
HOMO => LUMO+3 -0.11268

Transition dipole moment (debye):
X= 0.8625 Y= -0.2002 Z= 0.2345
Tot= 0.9160

Oscillator strength, f= 0.0086

Restricted Singlet Excited State 8:
2.7302 eV 454.13 nm

excitation X coeff.
------------ ---------
HOMO-3 => LUMO 0.85153
HOMO-3 => LUMO+1 0.12604
HOMO-2 => LUMO+1 -0.26610
HOMO-1 => LUMO+1 -0.29203
HOMO => LUMO 0.23787
HOMO => LUMO+2 0.13932

Transition dipole moment (debye):
X= 5.2292 Y= 3.2302 Z= 0.5136
Tot= 6.1679

Oscillator strength, f= 0.3939

Restricted Singlet Excited State 9:
3.0560 eV 405.71 nm

excitation X coeff.
------------ ---------
HOMO-4 => LUMO -0.21411
HOMO-3 => LUMO -0.16221
HOMO-3 => LUMO+2 -0.11509
HOMO => LUMO+2 0.93945

Transition dipole moment (debye):
X= 0.1461 Y= -1.2442 Z= -3.0535

Tot= 3.3005

Oscillator strength, f= 0.1262

Restricted Singlet Excited State 10:

3.0980 eV 400.21 nm

excitation	X coeff.
HOMO-4 => LUMO+1	0.75396
HOMO-3 => LUMO+1	0.14918
HOMO-2 => LUMO	0.11780
HOMO-2 => LUMO+2	-0.21848
HOMO-2 => LUMO+2	0.26659
HOMO => LUMO+3	-0.49329

Transition dipole moment (debye):
X= -0.3509 Y= 0.4947 Z= -0.0646
Tot= 0.6099

Oscillator strength, f= 0.0044

Restricted Singlet Excited State 11:

3.1223 eV 397.09 nm

excitation	X coeff.
HOMO-4 => LUMO	0.90768
HOMO-1 => LUMO+3	0.26559
HOMO => LUMO+2	0.24154
HOMO => LUMO+8	0.10068

Transition dipole moment (debye):
X= 1.0936 Y= 0.2235 Z= -1.0977
Tot= 1.5655

Oscillator strength, f= 0.0290

Restricted Singlet Excited State 12:

3.2095 eV 386.30 nm

excitation	X coeff.
HOMO-4 => LUMO+1	0.57124
HOMO-3 => LUMO+3	-0.24053
HOMO-2 => LUMO+2	0.53001
HOMO-1 => LUMO+2	-0.14452
HOMO => LUMO+3	0.49289

Transition dipole moment (debye):
X= 1.1601 Y= -2.0057 Z= 0.9037
Tot= 2.4870

Oscillator strength, f= 0.0753

Restricted Singlet Excited State 13:

3.2819 eV 377.78 nm

excitation	X coeff.
HOMO-9 => LUMO	-0.14136
HOMO-5 => LUMO	0.11636
HOMO-3 => LUMO+1	-0.12191
HOMO-1 => LUMO+2	-0.87206
HOMO => LUMO+3	-0.36694

Transition dipole moment (debye):
X= 0.3332 Y= -0.4694 Z= 0.1413
Tot= 0.5927

Oscillator strength, f= 0.0044

Restricted Singlet Excited State 14:

3.2998 eV 375.74 nm

excitation	X coeff.
HOMO-8 => LUMO	0.57376
HOMO-8 => LUMO+1	-0.52210
HOMO-8 => LUMO+4	-0.14112
HOMO-8 => LUMO+5	-0.14075
HOMO-7 => LUMO	-0.11002
HOMO-6 => LUMO	-0.25775
HOMO-6 => LUMO+1	0.15197
HOMO-5 => LUMO	0.29499
HOMO-5 => LUMO+1	-0.34221

Transition dipole moment (debye):
X= 0.0437 Y= 0.0078 Z= -0.0314
Tot= 0.0543

Oscillator strength, f= 0.0000

Restricted Singlet Excited State 15:
3.3003 eV 375.68 nm

excitation		X coeff.
HOMO-8 =>	LUMO+1	-0.13728
HOMO-7 =>	LUMO	0.61064
HOMO-7 =>	LUMO+1	0.62574
HOMO-7 =>	LUMO+4	-0.14990
HOMO-7 =>	LUMO+5	0.13082
HOMO-6 =>	LUMO	0.14907
HOMO-6 =>	LUMO+1	0.19854
HOMO-5 =>	LUMO	0.23658
HOMO-5 =>	LUMO+1	0.14604

Transition dipole moment (debye):
X= -0.0008 Y= -0.0194 Z= 0.0077
Tot= 0.0209

Oscillator strength, f= 0.0000

Restricted Singlet Excited State 16:
3.3051 eV 375.14 nm

excitation		X coeff.
HOMO-9 =>	LUMO	-0.19346
HOMO-9 =>	LUMO+1	0.24490
HOMO-8 =>	LUMO	0.39086
HOMO-8 =>	LUMO+1	-0.35373
HOMO-7 =>	LUMO+1	-0.10065
HOMO-6 =>	LUMO	0.52238
HOMO-6 =>	LUMO+1	-0.24968
HOMO-6 =>	LUMO+4	0.10091
HOMO-5 =>	LUMO	-0.28908
HOMO-5 =>	LUMO+1	0.34856
HOMO-5 =>	LUMO+5	-0.10747

Transition dipole moment (debye):
X= -0.1122 Y= 0.0691 Z= 0.2005
Tot= 0.2399

Oscillator strength, f= 0.0007

Restricted Singlet Excited State 17:
3.3066 eV 374.96 nm

excitation		X coeff.
HOMO-9 =>	LUMO	0.29690
HOMO-9 =>	LUMO+1	0.17003
HOMO-8 =>	LUMO	-0.12352
HOMO-7 =>	LUMO	-0.27316
HOMO-7 =>	LUMO+1	-0.23739
HOMO-6 =>	LUMO	0.40322
HOMO-6 =>	LUMO+1	0.53161
HOMO-6 =>	LUMO+5	-0.12477
HOMO-5 =>	LUMO	0.41062
HOMO-5 =>	LUMO+1	0.22268

Transition dipole moment (debye):
X= -0.0085 Y= -0.0292 Z= 0.1554
Tot= 0.1583

Oscillator strength, f= 0.0003

Restricted Singlet Excited State 18:
3.3541 eV 369.65 nm

excitation		X coeff.
HOMO-4 =>	LUMO	0.27739
HOMO-2 =>	LUMO+3	0.15924
HOMO-1 =>	LUMO+3	-0.91730

Transition dipole moment (debye):
X= 3.3030 Y= 0.5397 Z= -3.1973
Tot= 4.6286

Oscillator strength, f= 0.2725

Restricted Singlet Excited State 19:
3.5138 eV 352.85 nm

excitation X coeff.
------------ ---------
HOMO-14 => LUMO -0.13882
HOMO-14 => LUMO+1 0.24200
HOMO-12 => LUMO 0.44063
HOMO-12 => LUMO+1 -0.23780
HOMO-11 => LUMO -0.22887
HOMO-10 => LUMO 0.37037
HOMO-10 => LUMO+1 -0.48523
HOMO-9 => LUMO -0.27289
HOMO-5 => LUMO 0.19775
HOMO-2 => LUMO+2 -0.20486
HOMO => LUMO+3 0.16180

Transition dipole moment (debye):
X= 0.7011 Y= -1.0255 Z= 0.3112
Tot= 1.2807

Oscillator strength, f= 0.0219

Restricted Singlet Excited State 20:
3.5194 eV 352.29 nm

excitation X coeff.
------------ ---------
HOMO-14 => LUMO -0.31622
HOMO-14 => LUMO+1 -0.15302
HOMO-12 => LUMO -0.17282
HOMO-12 => LUMO+1 -0.44696
HOMO-11 => LUMO 0.30640
HOMO-11 => LUMO+1 0.41568
HOMO-10 => LUMO 0.44651
HOMO-10 => LUMO+1 0.11264
HOMO-9 => LUMO 0.13533
HOMO-5 => LUMO -0.10344
HOMO-3 => LUMO+2 0.19620
HOMO-2 => LUMO+2 0.13555
HOMO_2 => LUMO+3 0.17698
HOMO => LUMO+3 -0.10381

Transition dipole moment (debye):
X= -0.0603 Y= 0.7066 Z= -0.5898
Tot= 0.9223

Oscillator strength, f= 0.0114

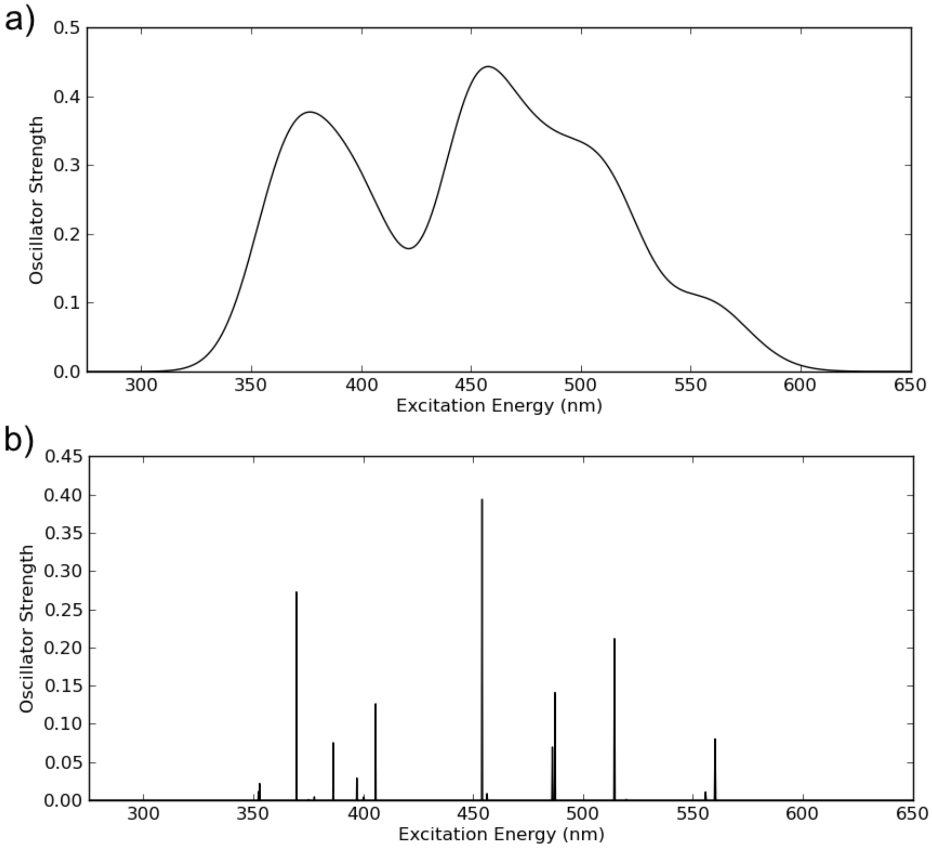

Figure S15. The simulated UV-visible absorbance spectrum of **PPDH** from TD-DFT (B3LYP/6-31G**), with (a) 35-nm-full-width-at-half-maximum electronic transitions and (b) zero-bandwidth. The energies of these transitions have not been scaled to match the experimental spectrum; instead, their wavelengths correspond to the singlet excited states listed above.

Part C: DFT-Optimized Molecular Structure of 5PPD

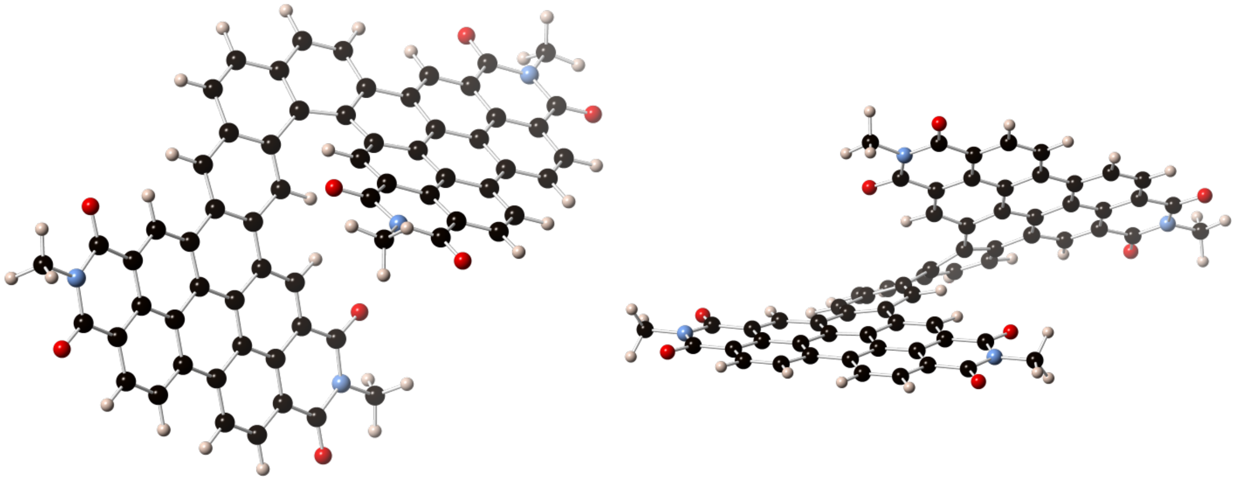

Figure S16. DFT-optimized model of *M*-**5PPD** (B3LYP/6-31G**) from different perspectives. Methyl groups substitute for the $CH(C_5H_{11})_2$ chains to simplify the calculation.

Total energy = -3354.322628 hartrees

	angstroms		
atom	x	y	z
C1	0.1469196515	3.4161950451	3.3037084652
C2	-0.7260532238	2.2957728989	3.4361563417
C3	-1.8305777977	2.3684355978	4.3290863537
C4	-2.0940603206	3.5523880971	5.0430088841
C5	-1.2559904971	4.6500002093	4.8800168198
C6	-0.1528040808	4.5769886424	4.0319355190
C7	-0.5042164416	1.0901466897	2.6998704525
C8	-1.3331067240	-0.0371710657	2.9018172153
C9	-2.4098078035	0.0646332229	3.8248041957
C10	-2.6651439453	1.2291433196	4.5036923367
C11	-3.8270962012	1.2835941700	5.4264904555
N12	-4.0456099466	2.4917869192	6.0965045891
C13	-3.2501733044	3.6440630435	5.9676995517
C14	1.3342293647	3.2912957748	2.4548258065
C15	1.5498722251	2.0747711117	1.7423927041
C16	0.6065402524	0.9980251040	1.8020400751
C17	2.2865700585	4.3136554049	2.3310353959
C18	3.4391483971	4.1553959045	1.5665134796
C19	3.6782190797	2.9596363974	0.8983957447
C20	2.7316690102	1.9212721651	0.9684885797
C21	2.9743251240	0.6847177581	0.3049581123
C22	2.0335561527	-0.3131659269	0.3283938440
C23	0.7922618961	-0.1655626631	1.0157074849
C24	4.9351693092	2.7830882993	0.1349492240

N25	5.1531001419	1.5272099086	-0.4499741530
C26	4.2519301260	0.4522418689	-0.4193639180
C27	-5.2004844984	2.5309367293	6.9996786162
C28	6.4263818598	1.3559947242	-1.1562732091
O29	5.7735336724	3.6674739685	0.0209990556
O30	4.5130814128	-0.6105891902	-0.9662535939
O31	-4.5742752756	0.3314500607	5.6107798095
O32	-3.5075336367	4.6646921715	6.5911032031
C33	-0.1880473155	-1.2413876421	1.0562725297
C34	-1.1088273683	-1.2480685376	2.1390349909
C35	-0.1933512249	-2.3618359634	0.1410693830
C36	-0.7520024225	-3.5825648413	0.5875724427
C37	-1.5214064966	-3.6102008977	1.7804429705
C38	-1.7698925134	-2.4624791841	2.4791192749
C39	0.2605202580	-2.3151254462	-1.2459263552
C40	0.4669962347	-3.5393702589	-1.9610931241
C41	0.0909439189	-4.7832571410	-1.3570536499
C42	-0.5564138216	-4.7903637274	-0.1638107521
C43	0.3805819714	-1.1246592529	-1.9733082485
C44	0.8109573243	-1.0787616838	-3.3041764951
C45	1.1614485575	-2.3016113302	-3.9610962692
C46	0.9466393967	-3.4991859453	-3.2744019370
H47	-1.4741985096	5.5553330356	5.4356932745
H48	0.4846873566	5.4484468284	3.9500549959
H49	-3.0887927586	-0.7601237854	3.9968324970
H50	2.1417239317	5.2534596121	2.8485345585
H51	4.1732675986	4.9498470880	1.4893805390
H52	2.2793397502	-1.2393423095	-0.1701606787
H53	-5.2482005199	3.5258779481	7.4349205490
H54	-6.1127814500	2.3098583507	6.4417168687
H55	-5.0861087748	1.7749900705	7.7796213650
H56	7.2540899441	1.5640855375	-0.4752999594
H57	6.4710945887	0.3304507318	-1.5140695396
H58	6.4869755031	2.0564918021	-1.9920671585
H59	-1.9539352028	-4.5533898268	2.1014942958
H60	-2.4184961164	-2.5019367947	3.3449853680
H61	0.2694204627	-5.7061257359	-1.9012126743
H62	-0.9309247690	-5.7188103898	0.2579914214
H63	1.1138030062	-4.4492795042	-3.7666387802
C64	1.3515116428	0.1869524914	-5.3749306979
C65	0.9113033634	0.1790496990	-4.0312187954
C66	0.5886589887	1.4150052941	-3.4122023605
C67	0.6634117656	2.6043255730	-4.0927876232
H68	0.2789166783	1.4691517700	-2.3770442993
C69	1.0740372771	2.6343265357	-5.4532388990
C70	1.4337537858	1.4194190236	-6.1014398558

C71	1.8743341604	1.4605175367	-7.4593092326
C72	1.9089331672	2.7002890270	-8.1128955005
C73	1.5394868527	3.8816430066	-7.4716126697
C74	1.1273940385	3.8609972484	-6.1450804914
H75	2.2376197446	2.7616435663	-9.1423460658
H76	1.5755066331	4.8336543937	-7.9895770104
C77	1.7449095496	-1.0397687646	-6.0197181267
C78	1.6717038927	-2.2668634569	-5.3233451080
C79	2.1115652666	-3.4481070217	-5.9739908545
C80	2.5851858199	-3.4340704764	-7.2616547710
H81	2.0983393570	-4.4051770417	-5.4699654237
C82	2.6464589763	-2.2152391240	-7.9904248048
C83	2.2237274373	-1.0056480397	-7.3699826897
C84	2.2880630317	0.2138251534	-8.1101964813
C85	2.7604815531	0.1704468805	-9.4296523620
C86	3.1764847723	-1.0199313857	-10.0246685369
C87	3.1281485077	-2.2123785141	-9.3145682200
H88	2.8147554630	1.0782505772	-10.0165378217
H89	3.5475277156	-1.0372775438	-11.0435491441
C90	0.3244451377	3.8614895212	-3.3785198872
N91	0.3935885550	5.0455191167	-4.1194997840
C92	0.7596096428	5.1325457504	-5.4743583420
C93	0.0483997041	6.2779094321	-3.4045844506
O94	0.7792921117	6.2081343790	-6.0575674168
O95	-0.0028185397	3.8890405190	-2.1984891538
C96	3.5863488629	-3.4687465302	-9.9550517265
N97	3.5072763446	-4.6411561029	-9.1893614041
C98	3.0328606257	-4.7136598240	-7.8723358229
C99	3.9713070691	-5.8720591566	-9.8380251968
O100	4.0209063003	-3.5109408870	-11.0983761878
O101	2.9937907436	-5.7765817519	-7.2658616193
H102	0.1763164522	7.1086734991	-4.0938805705
H103	-0.9848626097	6.2268388201	-3.0537721787
H104	0.6992428681	6.3921714851	-2.5356279746
H105	3.8183287050	-6.6919305526	-9.1407435622
H106	3.4091640517	-6.0378931193	-10.7589482514
H107	5.0291060129	-5.7801929520	-10.0943727526
H108	0.1029538329	-0.2069726208	-1.4791147279

Part D: DFT-Optimized Molecular Structure of PPPD

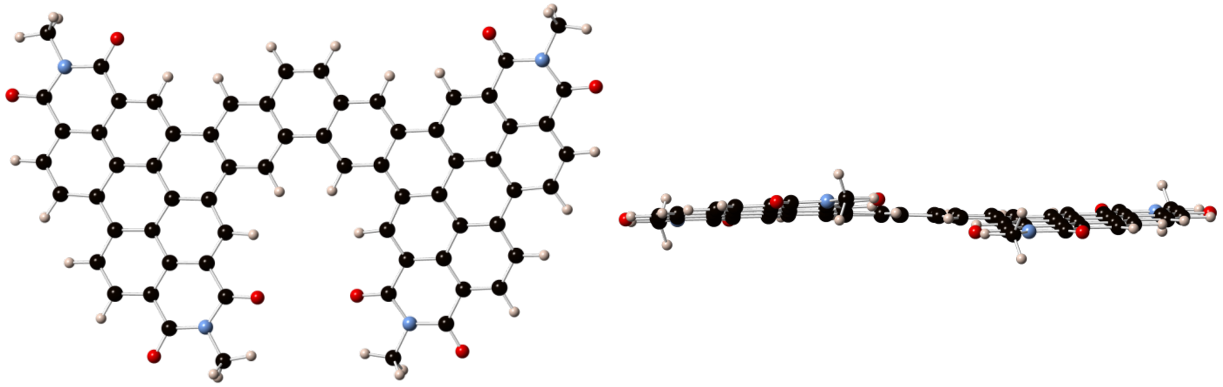

Figure S17. DFT-optimized model of **PPPD** (B3LYP/6-31G**) from different perspectives. Methyl groups substitute for the $CH(C_5H_{11})_2$ chains to simplify the calculation.

Total energy = -3354.339834 hartrees

angstroms

atom	x	y	z
C1	2.2393104037	1.3695645337	0.1298593516
C2	3.3905808018	0.5812658660	0.0858623426
C3	4.6541732192	1.2536402053	0.0800503198
C4	4.6939876124	2.6467550378	0.0764406851
C5	3.5382079681	3.4380577513	0.0985790450
C6	2.2708322392	2.7772207944	0.1504586052
C7	3.3745988638	-0.8879454743	0.0544182241
C8	4.6221162482	-1.5890307264	0.0778193147
C9	5.8625673954	-0.8580623608	0.0941979397
C10	5.8778815086	0.4944709057	0.0833757129
C11	3.6000114661	4.8920927198	0.0707157385
C12	2.4083459975	5.6516311901	0.1070743265
C13	1.1317144036	4.9920060128	0.1921057489
C14	1.0547819105	3.5772121370	0.2262404904
C15	4.8417213631	5.5769160353	-0.0022806059
C16	4.9145354134	6.9458687760	-0.0405448313
C17	3.7306090145	7.7308977932	-0.0074076972
C18	2.4648786449	7.0840263559	0.0688831931
C19	1.2810316908	7.8797894341	0.1066840524
C20	-0.0194403366	7.2109476909	0.1990390723
C21	-0.0624457142	5.7837442363	0.2474785922
C22	-1.3237390941	5.1336558411	0.3547854191
C23	-1.3754759287	3.7146271627	0.4198934615
C24	-0.2235761942	2.9710181397	0.3527780664
C25	-1.2275934136	7.9203987987	0.2467620221
C26	-2.4569354334	7.2714909780	0.3459986730
C27	-2.5144734553	5.8852177412	0.4036351670

C28	3.8190611465	9.1364815682	-0.0495407481
C29	2.6568249437	9.8964445518	-0.0166763016
C30	1.4120151161	9.2749925733	0.0605498303
C31	-3.8302454392	5.2106406661	0.5155750142
N32	-3.8238213616	3.8115314908	0.5876766520
C33	-2.6748172553	3.0061982488	0.5717750038
C34	6.2495143334	7.5935956428	-0.1179844801
N35	6.2713696340	8.9917613880	-0.1553415606
C36	5.1350117896	9.8190680409	-0.1276852775
O37	-2.7465622732	1.7898436671	0.6757344479
O38	-4.8890502624	5.8238322806	0.5497247523
O39	7.2960548096	6.9586530434	-0.1484103414
O40	5.2370933917	11.0378080042	-0.1658499109
C41	-5.1332133668	3.1626800489	0.7086922367
C42	7.5957270829	9.6179500477	-0.2302583791
C43	2.2069167597	-1.6500804130	-0.0067730857
C44	2.2054582421	-3.0578067793	-0.0283948164
C45	3.4569184403	-3.7477665213	0.0363735344
C46	4.6299057540	-2.9827351825	0.0774031833
C47	0.9717352744	-3.8292063621	-0.1190843148
C48	1.0166670964	-5.2453000721	-0.1004216079
C49	2.2769483336	-5.9346858906	-0.0067682123
C50	3.4850499284	-5.2032813564	0.0552979023
C51	-0.2925844627	-3.1939360247	-0.2431488427
C52	-1.4602621355	-3.9090256415	-0.3294399213
C53	-1.4402776126	-5.3294601178	-0.2880853720
C54	-0.1943974600	-6.0084346591	-0.1783814483
C55	-0.1830273401	-7.4361646045	-0.1502447065
C56	1.0993324102	-8.1360762499	-0.0496199820
C57	2.2999210864	-7.3683500363	0.0160172747
C58	3.5487080230	-8.0453392447	0.1047770287
C59	4.7496815756	-7.2884843057	0.1674859594
C60	4.7093229876	-5.9177205596	0.1428742343
C61	1.1964913756	-9.5341900124	-0.0201744748
C62	2.4249039046	-10.1854849399	0.0686665787
C63	3.6035264336	-9.4530414542	0.1306728308
C64	-2.6478648648	-6.0512428888	-0.3612102116
C65	-2.6213124717	-7.4389310620	-0.3261372007
C66	-1.4072659322	-8.1156518831	-0.2237421950
C67	-2.7414431691	-3.1670236295	-0.4757927300
N68	-3.9084591508	-3.9446034657	-0.5182046534
C69	-3.9470997715	-5.3445650327	-0.4739594626
C70	4.9014816055	-10.1673597135	0.2220196743
N71	6.0559725243	-9.3672087918	0.2808037298
C72	6.0675333158	-7.9683898514	0.2615818930
O73	-5.0190008640	-5.9326926430	-0.5304376069

O74	-2.7840228353	-1.9473104728	-0.5543075370
O75	7.1278727002	-7.3588389715	0.3199472360
O76	4.9745218708	-11.3885218640	0.2451556123
C77	7.3633850844	-10.0255855535	0.3713123396
C78	-5.2010746951	-3.2625539069	-0.6378133148
H79	1.2786251550	0.8756614303	0.1507783198
H80	5.6716179869	3.1116977176	0.0593273200
H81	5.7809779642	5.0413838634	-0.0342087246
H82	-0.3390110603	1.8967081183	0.4127253560
H83	-1.2228380636	9.0026403905	0.2082212053
H84	-3.3848010940	7.8314866420	0.3823725507
H85	2.7420066612	10.9769024336	-0.0505311360
H86	0.5307702023	9.9039185779	0.0867436679
H87	-4.9699618428	2.0879901417	0.7185268675
H88	-5.6240109822	3.4840055441	1.6300831929
H89	-5.7645190309	3.4516926896	-0.1336974451
H90	7.4525782710	10.6953276411	-0.2498397397
H91	8.1926719220	9.3243050820	0.6357196024
H92	8.1132304421	9.2819488130	-1.1312675475
H93	1.2581882233	-1.1342089568	-0.0426633088
H94	5.5962675705	-3.4701333295	0.1052416102
H95	-0.3836477607	-2.1162653949	-0.2844352767
H96	5.6598739591	-5.4043121539	0.1974828525
H97	0.3012149851	-10.1414993185	-0.0683772535
H98	2.4847020925	-11.2679237377	0.0896899936
H99	-3.5605613757	-7.9780281008	-0.3815458201
H100	-1.4285106207	-9.1975308261	-0.2024636214
H101	7.1944002715	-11.0993877208	0.3692209489
H102	7.9842446486	-9.7312739070	-0.4773707102
H103	7.8698794323	-9.7175418683	1.2884272234
H104	-5.0123325437	-2.1920492603	-0.6237269799
H105	-5.6902645200	-3.5527577225	-1.5702868278
H106	-5.8472100636	-3.5534553135	0.1925511572
H107	6.7911058357	-1.4217677017	0.1071318725
H108	6.8190427302	1.0370078981	0.0848507800

Part E: Molecular Orbitals, DFT-Optimized Molecular Structure, and TD-DFT Excited State Calculations of PPDH-OPe

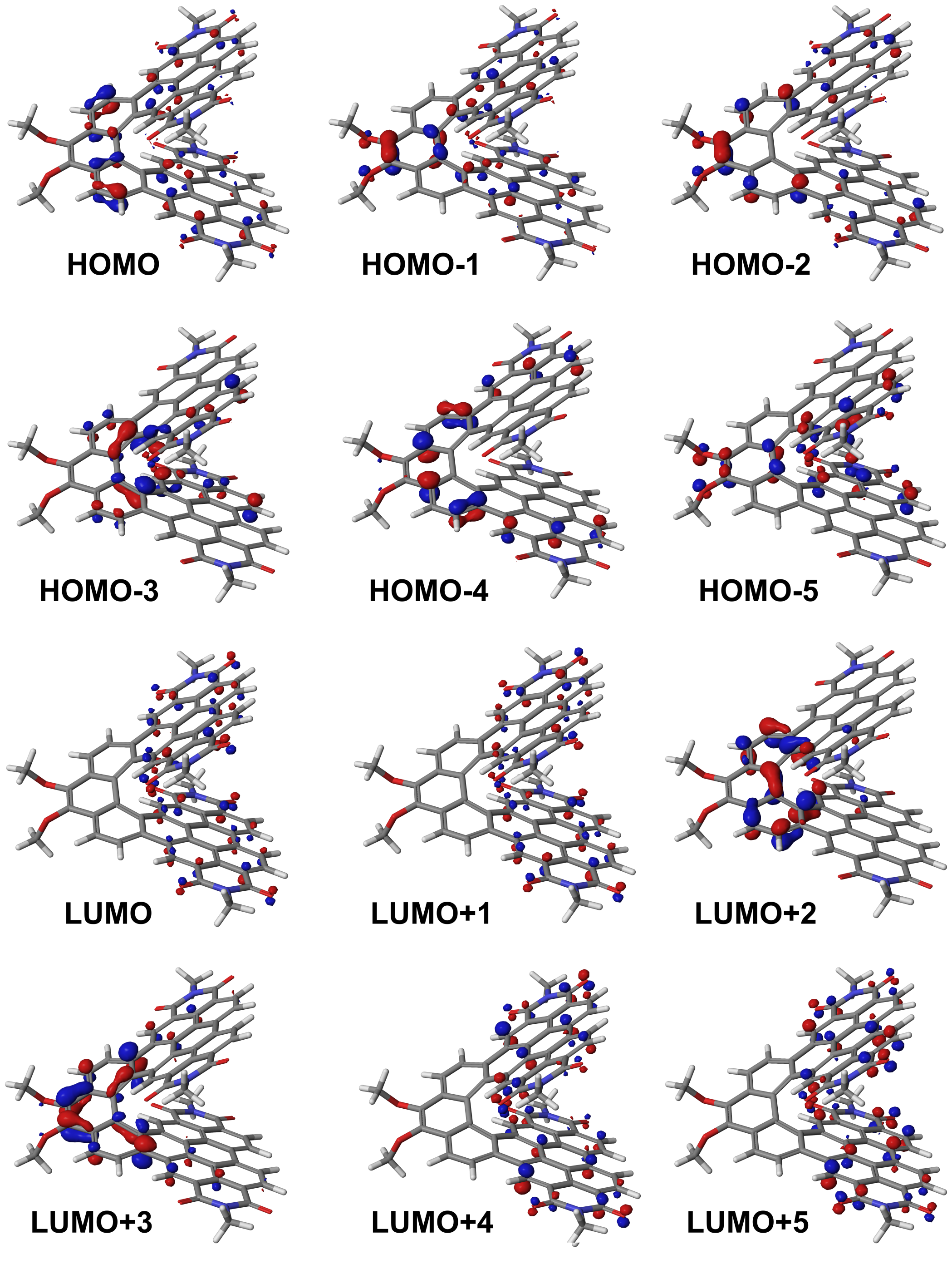

Figure S18. Highest- and lowest-unoccupied molecular orbitals of **PPDH-OPe** by DFT (B3LYP/6-31G**). Orbital isosurfaces are illustrated at 0.05 electrons Bohr^{-3}. We substitute methyl groups for the $CH(C_5H_{11})_2$ and C_5H_{11} chains to simplify the calculations.

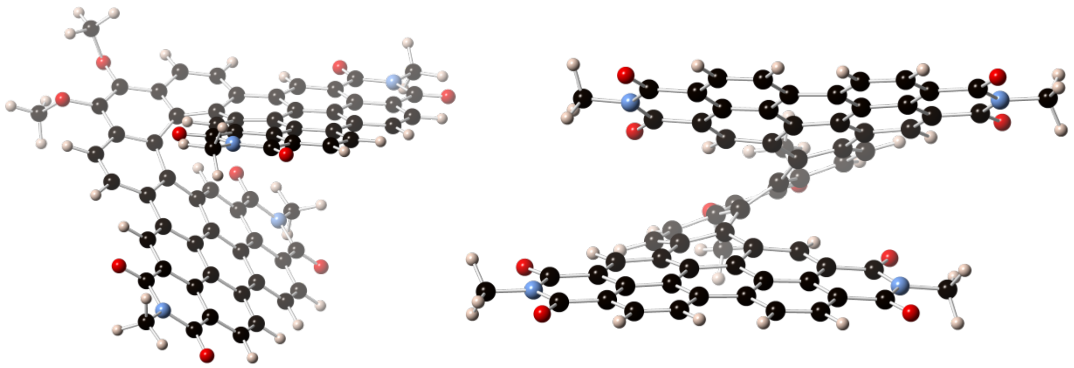

Figure S19. DFT-optimized model of *M*-**PPDH-OPe** (B3LYP/6-31G**) from different perspectives. Methyl groups substitute for the $CH(C_5H_{11})_2$ and C_5H_{11} chains to simplify the calculation.

Total energy = -3583.346743 hartrees

angstroms

atom	x	y	z
C1	-2.2733393585	2.3430114030	0.4296357562
C2	-2.4072544101	0.9255901905	0.5238128084
C3	-3.7016047842	0.3521714363	0.6660156968
C4	-4.8473198783	1.1695465076	0.6655322870
C5	-4.7059673021	2.5475708611	0.5350910721
C6	-3.4412920582	3.1219944642	0.4252128530
C7	-1.2628117011	0.0664563982	0.4914387514
C8	-1.4110565674	-1.3298374598	0.6619526493
C9	-2.7200317874	-1.8616995976	0.8261298560
C10	-3.8303376161	-1.0577030612	0.8148351905
C11	-5.1713995617	-1.6791654167	0.9613868674
N12	-6.2721768412	-0.8154401972	0.9380636229
C13	-6.2039048715	0.5822707877	0.8018175560
C14	-0.9297005362	2.9230068222	0.3812699269
C15	0.2011857869	2.0519437553	0.3545297477
C16	0.0456297687	0.6267900865	0.3481600565
C17	-0.7025884139	4.3089191108	0.3874447860
C18	0.5842348029	4.8418060431	0.3947653642
C19	1.6937160465	4.0023236433	0.3855314787
C20	1.5100593592	2.6081610655	0.3510846585
C21	2.6359724760	1.7361613242	0.3566354988
C22	2.4652162789	0.3765392410	0.3055553316
C23	1.1748457979	-0.2246170570	0.2402794229
C24	3.0577482297	4.5806536072	0.4423830487
N25	4.1317333871	3.6780717161	0.4734999217
C26	4.0181683265	2.2801275244	0.4250797652
C27	-7.5888709605	-1.4459613023	1.0770016017
C28	5.4708688814	4.2705185753	0.5524465120
O29	3.2682777530	5.7859902271	0.4735417993

O30	5.0088373687	1.5602918161	0.4320052348
O31	-5.3369717530	-2.8845680964	1.0979008283
O32	-7.2166207371	1.2691742567	0.7966380082
C33	1.0026470111	-1.6667312890	0.2232594489
C34	-0.2502441153	-2.1923859496	0.6255221960
C35	2.0803839585	-2.5982634721	-0.0620498475
C36	2.0216628332	-3.8749476585	0.5579837889
C37	0.7852005094	-4.3395224417	1.0892312563
C38	-0.3329874941	-3.5608545296	1.0265736002
C39	3.2206802830	-2.3160539596	-0.9055718145
C40	4.4146697766	-3.0460614411	-0.6632764252
C41	4.3822139406	-4.2455525683	0.1156416926
C42	3.1928599186	-4.6930474846	0.6411037298
C43	3.2291619881	-1.3743031230	-2.0115449914
C44	4.4727080551	-0.8378236675	-2.4280490469
C45	5.6790661064	-1.4502668674	-1.9725397566
C46	5.6451040440	-2.5767217187	-1.2039676630
H47	-5.6002145705	3.1608975706	0.5307004891
H48	-3.3744691208	4.1997364034	0.3407548363
H49	-2.8860647187	-2.9256810049	0.9275268445
H50	-1.5406626697	4.9946925188	0.4066366109
H51	0.7460060878	5.9138133036	0.4199845836
H52	3.3548317394	-0.2330029098	0.3568951647
H53	-8.3396625979	-0.6614872630	1.0287111474
H54	-7.7363833518	-2.1716972235	0.2745486086
H55	-7.6467818443	-1.9761654447	2.0300956719
H56	5.5511769926	4.8802886168	1.4548132249
H57	6.1922790909	3.4575849137	0.5729464143
H58	5.6435744891	4.9151742924	-0.3117780204
H59	0.7479251444	-5.3431173295	1.4954439782
H60	-1.2756517279	-3.9680158553	1.3672932829
O61	5.5568913441	-4.9363333176	0.3035326814
O62	3.0934370132	-5.9164534513	1.2603472635
H63	6.6355550831	-1.0732045361	-2.3106399598
H64	6.5530808061	-3.1100183080	-0.9491677665
C65	3.3134216966	0.7057471498	-3.9806318121
C66	4.5018883440	0.2848259841	-3.3402578649
C67	5.7011696742	1.0117573295	-3.5805112936
C68	5.7261767093	2.1021170374	-4.4122633989
H69	6.6256852121	0.7521209461	-3.0814169333
C70	4.5479697991	2.5226757550	-5.0917288832
C71	3.3336614175	1.8100802830	-4.8908389150
C72	2.1649337045	2.2011852319	-5.6081804780
C73	2.2476718879	3.3139519171	-6.4586423041
C74	3.4344789229	4.0226722432	-6.6269072292
C75	4.5886550475	3.6323679244	-5.9561512117

H76	1.3739881078	3.6419082902	-7.0085390246
H77	3.4840807136	4.8825565973	-7.2855637170
C78	2.0969371345	-0.0131721770	-3.7583272819
C79	2.0525330576	-1.0585598134	-2.8006621652
C80	0.8792867422	-1.8661333993	-2.7541277168
C81	-0.2234994558	-1.5925566201	-3.5213754824
H82	0.8432093478	-2.7527523794	-2.1401345487
C83	-0.2318379382	-0.4787794613	-4.4077518690
C84	0.9399403044	0.3144157062	-4.5378978490
C85	0.9446709786	1.4091308487	-5.4532286925
C86	-0.2226796704	1.6685415205	-6.1880130395
C87	-1.3619603104	0.8794032802	-6.0595103752
C88	-1.3730450561	-0.1975808320	-5.1798625672
H89	-0.2477273272	2.4925483625	-6.8905473713
H90	-2.2519151487	1.0792480314	-6.6459546706
C91	7.0011100954	2.8419249441	-4.5936713281
N92	6.9734830053	3.9462061989	-5.4533812043
C93	5.8447101482	4.3945843169	-6.1608709190
C94	8.2377624290	4.6712747837	-5.6146898898
O95	5.9047476989	5.3655378119	-6.9026345988
O96	8.0410106422	2.5248337946	-4.0315958580
C97	-2.5757963036	-1.0562171282	-5.0864456879
N98	-2.5082921432	-2.1553604655	-4.2167745349
C99	-1.4044266541	-2.4886744391	-3.4180837824
C100	-3.7001012099	-3.0075984790	-4.1567799596
O101	-3.5928224373	-0.8485737330	-5.7341077007
O102	-1.4271147002	-3.4608557332	-2.6743170858
H103	8.0592085579	5.5020051283	-6.2926408267
H104	8.5816412198	5.0324390747	-4.6432598343
H105	9.0000660330	4.0017559712	-6.0189399438
H106	-3.5083476596	-3.7949781884	-3.4322494142
H107	-4.5658756433	-2.4127208191	-3.8598400756
H108	-3.9002775394	-3.4339210051	-5.1422607605
C109	3.7097615010	-5.9963918456	2.5570438285
C110	5.6849653380	-6.1354269017	-0.4796815076
H111	4.7825586886	-5.7946790881	2.4953524714
H112	3.2403519228	-5.2903265097	3.2518315047
H113	3.5428918894	-7.0164797563	2.9075475679
H114	4.8906542197	-6.8477502867	-0.2395373699
H115	5.6605471531	-5.9021960915	-1.5506040958
H116	6.6564351525	-6.5615311568	-0.2228117496

PPDH-OPe Frontier Molecular Orbital Energies (eV):

LUMO+9	LUMO+8	LUMO+7	LUMO+6	LUMO+5
-1.22261	-1.35322	-1.60057	-1.6033	-1.73146
LUMO+4	LUMO+3	LUMO+2	LUMO+1	LUMO
-1.78806	-2.08521	-2.25637	-3.23108	-3.24768
HOMO	HOMO-1	HOMO-2	HOMO-3	HOMO-4
-5.84555	-5.92066	-6.07848	-6.34978	-6.79387
HOMO-5	HOMO-6	HOMO-7	HOMO-8	HOMO-9
-7.20884	-7.26136	-7.26191	-7.26953	-7.2698

Restricted Singlet Excited State 1:
2.1994 eV 563.71 nm

excitation	X coeff.
HOMO-3 => LUMO	-0.10911
HOMO-1 => LUMO+1	-0.49546
HOMO => LUMO	-0.85248

Transition dipole moment (debye):
X= 2.1911 Y= 0.4411 Z= -1.4716
Tot= 2.6760

Oscillator strength, f= 0.0597

Restricted Singlet Excited State 2:
2.2144 eV 559.90 nm

excitation	X coeff.
HOMO-2 => LUMO	0.10531
HOMO-1 => LUMO	-0.42892
HOMO => LUMO+1	-0.88307

Transition dipole moment (debye):
X= 0.4612 Y= -1.2410 Z= 0.6630
Tot= 1.4806

Oscillator strength, f= 0.0184

Restricted Singlet Excited State 3:
2.2955 eV 540.12 nm

excitation	X coeff.
HOMO-3 => LUMO	-0.13048
HOMO-2 => LUMO	-0.13009
HOMO-2 => LUMO+1	0.38241
HOMO-1 => LUMO	-0.35829
HOMO-1 => LUMO+1	0.70703
HOMO => LUMO	-0.37567
HOMO => LUMO+1	0.17696

Transition dipole moment (debye):
X= 2.7541 Y= 1.1239 Z= 0.7256
Tot= 3.0618

Oscillator strength, f= 0.0816

Restricted Singlet Excited State 4:
2.2983 eV 539.47 nm

excitation	X coeff.
HOMO-3 => LUMO+1	0.11806
HOMO-2 => LUMO	-0.28694
HOMO-2 => LUMO+1	-0.18368
HOMO-1 => LUMO	-0.77348
HOMO-1 => LUMO+1	-0.33324

HOMO => LUMO 0.17650
HOMO => LUMO+1 0.32817

Transition dipole moment (debye):
X= -0.5787 Y= -1.8561 Z= 0.1862
Tot= 1.9532

Oscillator strength, f= 0.0332

Restricted Singlet Excited State 5:
2.4940 eV 497.14 nm

excitation	X coeff.
HOMO-3 => LUMO+1	0.20489
HOMO-2 => LUMO	0.90594
HOMO-1 => LUMO	-0.23055
HOMO-1 => LUMO+2	0.12595
HOMO => LUMO+1	0.18552
HOMO => LUMO+3	-0.10745

Transition dipole moment (debye):
X= -0.8142 Y= 2.1419 Z= -1.0870
Tot= 2.5361

Oscillator strength, f= 0.0608

Restricted Singlet Excited State 6:
2.5010 eV 495.73 nm

excitation	X coeff.
HOMO-3 => LUMO	-0.40388
HOMO-2 => LUMO+1	-0.83391
HOMO-1 => LUMO+1	0.29490
HOMO => LUMO	-0.15501

Transition dipole moment (debye):
X= 4.6828 Y= 1.5194 Z= -2.1029
Tot= 5.3534

Oscillator strength, f= 0.2718

Restricted Singlet Excited State 7:
2.7070 eV 458.01 nm

excitation	X coeff.
HOMO-3 => LUMO+1	0.92809
HOMO-2 => LUMO	-0.18354
HOMO-1 => LUMO	0.12435
HOMO-1 => LUMO+2	0.17491
HOMO => LUMO+1	-0.15612
HOMO => LUMO+3	-0.11575

Transition dipole moment (debye):
X= -0.2535 Y= 0.7636 Z= -0.2444
Tot= 0.8409

Oscillator strength, f= 0.0073

Restricted Singlet Excited State 8:
2.7263 eV 454.77 nm

excitation	X coeff.
HOMO-3 => LUMO	-0.86865
HOMO-2 => LUMO+1	0.31278
HOMO-1 => LUMO+1	-0.18374
HOMO => LUMO	0.23897
HOMO => LUMO+2	0.15343

Transition dipole moment (debye):
X= -5.1380 Y= -3.2027 Z= -0.6309
Tot= 6.0872

Oscillator strength, f= 0.3831

Restricted Singlet Excited State 9:
3.0251 eV 409.86 nm

excitation	X coeff.
HOMO-4 => LUMO	-0.17602
HOMO-3 => LUMO	0.17070
HOMO-3 => LUMO+2	0.11572
HOMO => LUMO+2	0.94588

Transition dipole moment (debye):
X= -0.0874 Y= 1.1406 Z= 2.9201
Tot= 3.1362

Oscillator strength, f= 0.1128

Restricted Singlet Excited State 10:
3.0452 eV 407.15 nm

excitation	X coeff.
HOMO-4 => LUMO+1	0.52857
HOMO-3 => LUMO+1	-0.19286
HOMO-3 => LUMO+3	-0.12062
HOMO-2 => LUMO+2	0.26617
HOMO-1 => LUMO+2	0.60270
HOMO => LUMO+3	-0.44421

Transition dipole moment (debye):
X= 0.1619 Y= -0.4630 Z= -0.0100
Tot= 0.4906

Oscillator strength, f= 0.0028

Restricted Singlet Excited State 11:
3.0981 eV 400.19 nm

excitation	X coeff.
HOMO-4 => LUMO	0.92761
HOMO-2 => LUMO+3	-0.11865
HOMO-1 => LUMO+3	0.20517
HOMO => LUMO+2	0.20068
HOMO => LUMO+8	0.10152

Transition dipole moment (debye):
X= -1.4040 Y= -0.2670 Z= 1.1252
Tot= 1.8189

Oscillator strength, f= 0.0389

Restricted Singlet Excited State 12:
3.1236 eV 396.93 nm

excitation	X coeff.
HOMO-4 => LUMO+1	-0.77321
HOMO-3 => LUMO+3	-0.17299
HOMO-2 => LUMO+2	0.39945
HOMO-1 => LUMO+2	0.39257
HOMO-1 => LUMO+8	0.10120

Transition dipole moment (debye):
X= 0.5488 Y= -1.0852 Z= 0.5105
Tot= 1.3189

Oscillator strength, f= 0.0206

Restricted Singlet Excited State 13:
3.2659 eV 379.63 nm

excitation	X coeff.
HOMO-5 => LUMO	-0.19165
HOMO-2 => LUMO+2	-0.56082
HOMO-1 => LUMO+2	0.59300
HOMO => LUMO+3	0.50791

Transition dipole moment (debye):
X= -0.0424 Y= -0.0329 Z= 0.0657
Tot= 0.0849

Oscillator strength, f= 0.0001

Restricted Singlet Excited State 14:
3.3023 eV 375.44 nm

excitation	X coeff.
HOMO-9 => LUMO	0.15460
HOMO-9 => LUMO+1	-0.18845
HOMO-9 => LUMO+4	0.10635
HOMO-9 => LUMO+5	-0.11989
HOMO-7 => LUMO	0.14153
HOMO-7 => LUMO+1	-0.39011
HOMO-6 => LUMO	-0.64551

HOMO-6 => LUMO+1 0.53091

Transition dipole moment (debye):
X= -0.0540 Y= -0.0281 Z= 0.0060
Tot= 0.0612

Oscillator strength, f= 0.0000

Restricted Singlet Excited State 15:
3.3025 eV 375.43 nm

excitation		X coeff.
HOMO-8 =>	LUMO	-0.15502
HOMO-8 =>	LUMO+1	-0.19297
HOMO-8 =>	LUMO+4	-0.11016
HOMO-8 =>	LUMO+5	-0.11523
HOMO-7 =>	LUMO	0.65526
HOMO-7 =>	LUMO+1	0.51639
HOMO-6 =>	LUMO	0.15859
HOMO-6 =>	LUMO+1	0.37967

Transition dipole moment (debye):
X= -0.0733 Y= -0.0450 Z= -0.0056
Tot= 0.0862

Oscillator strength, f= 0.0001

Restricted Singlet Excited State 16:
3.3107 eV 374.50 nm

excitation		X coeff.
HOMO-9 =>	LUMO+1	-0.32565
HOMO-8 =>	LUMO	0.68351
HOMO-8 =>	LUMO+1	0.52261
HOMO-7 =>	LUMO	0.14764
HOMO-7 =>	LUMO+1	0.14044
HOMO-7 =>	LUMO+5	-0.11931
HOMO-6 =>	LUMO	0.11695
HOMO-5 =>	LUMO	0.13704

Transition dipole moment (debye):
X= 0.2030 Y= -0.0529 Z= -0.1712
Tot= 0.2707

Oscillator strength, f= 0.0009

Restricted Singlet Excited State 17:
3.3110 eV 374.46 nm

excitation		X coeff.
HOMO-9 =>	LUMO	-0.66302
HOMO-9 =>	LUMO+1	0.53541
HOMO-8 =>	LUMO+1	0.30119
HOMO-7 =>	LUMO	0.14936
HOMO-6 =>	LUMO	-0.14137
HOMO-6 =>	LUMO+1	0.16417
HOMO-6 =>	LUMO+5	-0.11709
HOMO-5 =>	LUMO+1	0.12645
HOMO-1 =>	LUMO+3	0.13832

Transition dipole moment (debye):
X= 0.3953 Y= 0.0800 Z= -0.4888
Tot= 0.6337

Oscillator strength, f= 0.0050

Restricted Singlet Excited State 18:
3.3286 eV 372.48 nm

excitation		X coeff.
HOMO-4 =>	LUMO	0.21604
HOMO-1 =>	LUMO+3	-0.93422

Transition dipole moment (debye):
X= -4.0268 Y= -0.8034 Z= 3.4747
Tot= 5.3790

Oscillator strength, f= 0.3652

Restricted Singlet Excited State 19:
3.4495 eV 359.43 nm

excitation	X coeff.
HOMO-15 => LUMO+1	-0.11213
HOMO-12 => LUMO	-0.14599
HOMO-11 => LUMO+1	0.14764
HOMO-5 => LUMO	0.45510
HOMO-4 => LUMO+1	0.25132
HOMO-2 => LUMO+2	0.48036
HOMO => LUMO+3	0.59245

Transition dipole moment (debye):
X= -2.1021 Y= 3.5609 Z= -1.4997
Tot= 4.3986

Oscillator strength, f= 0.2531

Restricted Singlet Excited State 20:
3.4649 eV 357.82 nm

excitation	X coeff.
HOMO-11 => LUMO	0.15455
HOMO-5 => LUMO+1	0.78115
HOMO-3 => LUMO+2	-0.48436
HOMO-2 => LUMO+3	0.22547
HOMO => LUMO+4	-0.12282

Transition dipole moment (debye):
X= -0.1367 Y= -0.0575 Z= -0.2912
Tot= 0.3268

Oscillator strength, f= 0.0014

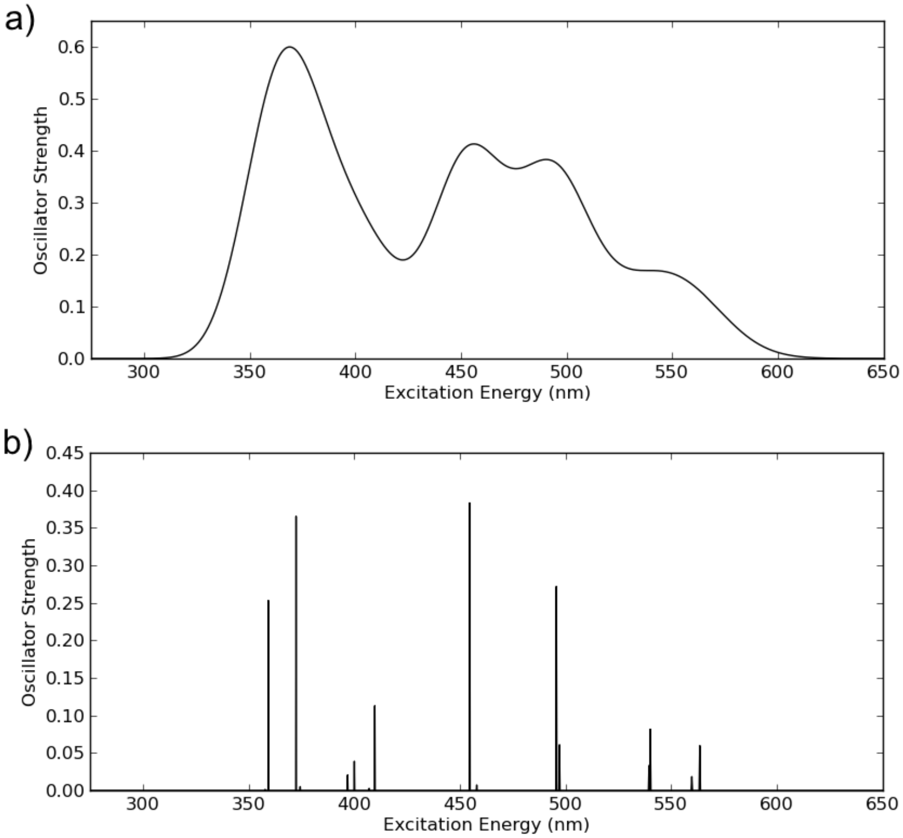

Figure S20. The simulated UV-visible absorbance spectrum of **PPDH-OPe** from TD-DFT (B3LYP/6-31G**), with (a) 35-nm-full-width-at-half-maximum electronic transitions and (b) zero-bandwidth. The energies of these transitions have not been scaled to match the experimental spectrum; instead, their wavelengths correspond to the singlet excited states listed above.

Part F: DFT-Optimized Molecular Structure of 5PPD-OPe

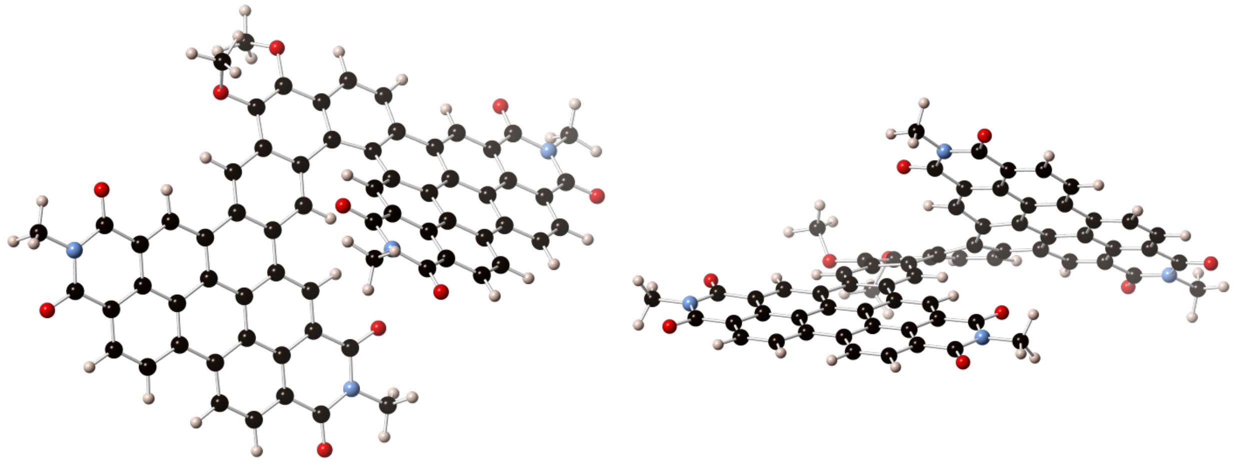

Figure S21. DFT-optimized model of *M*-**5PPD-OPe** (B3LYP/6-31G**) from different perspectives. Methyl groups substitute for the $CH(C_5H_{11})_2$ and C_5H_{11} chains to simplify the calculation.

Total energy = -3583.364068 hartrees

angstroms

atom	x	y	z
C1	0.2230794379	3.4575002304	3.3252731050
C2	-0.7074600619	2.3803165053	3.4137239584
C3	-1.8425784290	2.5041421581	4.2611978348
C4	-2.0788959710	3.6987486123	4.9670207894
C5	-1.1880769068	4.7585871742	4.8375895284
C6	-0.0550967433	4.6339039835	4.0366300681
C7	-0.5166206706	1.1706623001	2.6747686431
C8	-1.4107361173	0.0868283765	2.8308097745
C9	-2.5135561233	0.2362913540	3.7159087352
C10	-2.7366757665	1.4056911315	4.3970124116
C11	-3.9258990856	1.5103104366	5.2804757568
N12	-4.1114507346	2.7256753982	5.9482766376
C13	-3.2639607995	3.8431320253	5.8473400876
C14	1.4419361912	3.2730766145	2.5356045410
C15	1.6243759289	2.0544318103	1.8169820508
C16	0.6232058073	1.0293788731	1.8212443419
C17	2.4599678948	4.2367869919	2.4776292034
C18	3.6378098777	4.0215763363	1.7667320135
C19	3.8365558309	2.8269571804	1.0835337337
C20	2.8301474127	1.8443284288	1.0944949157
C21	3.0257131794	0.6090331413	0.4137350819
C22	2.0314730359	-0.3347122588	0.3770411431
C23	0.7771374161	-0.1322754747	1.0245551449
C24	5.1133448328	2.5903550136	0.3698988179
N25	5.2730274894	1.3413195300	-0.2474592880
C26	4.3145802010	0.3154002691	-0.2633614528

C27	-5.2910689567	2.8143146164	6.8151628247
C28	6.5566456836	1.1129737333	-0.9179040192
O29	6.0107357959	3.4211490273	0.3168287135
O30	4.5385792204	-0.7534796650	-0.8152290754
O31	-4.7216266773	0.5931926490	5.4365358287
O32	-3.5002369787	4.8754823228	6.4596991706
C33	-0.2577689789	-1.1554509390	1.0170241100
C34	-1.2195102164	-1.1263910965	2.0631970949
C35	-0.2838963026	-2.2620920320	0.0863737488
C36	-0.9168494244	-3.4587420674	0.4967389044
C37	-1.7351185768	-3.4630370151	1.6568677535
C38	-1.9555026567	-2.3091424797	2.3557365083
C39	0.2053267217	-2.2128039074	-1.2856235310
C40	0.3766400820	-3.4325137480	-2.0169939294
C41	-0.0354779058	-4.6792119510	-1.4300286445
C42	-0.7539849518	-4.6739273251	-0.2639191586
C43	0.3900301006	-1.0149645272	-1.9865467015
C44	0.8102044855	-0.9648107508	-3.3203672617
C45	1.1101653498	-2.1915324724	-3.9951732629
C46	0.8667104910	-3.3944138538	-3.3260169686
H47	-1.3901398940	5.6747120984	5.3814297092
H48	0.6217708412	5.4766992303	3.9760603481
H49	-3.2366637964	-0.5563823862	3.8547609334
H50	2.3485732119	5.1732282835	3.0093741895
H51	4.4220503844	4.7701471832	1.7425383698
H52	2.2443380275	-1.2624259094	-0.1346174790
H53	-5.2957728946	3.8022673133	7.2685676492
H54	-6.1968583822	2.6575081796	6.2255209464
H55	-5.2446373868	2.0380345957	7.5818183719
H56	7.3718604430	1.2012658293	-0.1964573189
H57	6.5328760980	0.1148055019	-1.3477125574
H58	6.7063671790	1.8650439603	-1.6952541342
H59	-2.2142416012	-4.3902790602	1.9459009870
H60	-2.6446059234	-2.3244626547	3.1907031215
H61	0.9934617097	-4.3440964736	-3.8258990774
C62	1.3297371827	0.3052162356	-5.3949310899
C63	0.9139952488	0.2958358083	-4.0430079112
C64	0.5783818614	1.5288653923	-3.4254533148
C65	0.6260736311	2.7175616315	-4.1092597968
H66	0.2614818044	1.5778318025	-2.3927281630
C67	1.0308092125	2.7507446091	-5.4707443896
C68	1.3976180743	1.5386597996	-6.1210908936
C69	1.8171749081	1.5811087346	-7.4853529480
C70	1.8357398562	2.8204035038	-8.1395836801
C71	1.4649639176	3.9995695970	-7.4952554129
C72	1.0650030429	3.9765933409	-6.1652506612
H73	2.1426858497	2.8812515225	-9.1765289736
H74	1.4823130554	4.9507058805	-8.0157601313
C75	1.6902686883	-0.9245738192	-6.0527917128

C76	1.6022612127	-2.1555324734	-5.3645141397
C77	2.0027408269	-3.3413214850	-6.0326231531
C78	2.4504144578	-3.3269133377	-7.3300192613
H79	1.9799274453	-4.3001735249	-5.5322894898
C80	2.5207636154	-2.1041215626	-8.0515336844
C81	2.1397234318	-0.8903140156	-7.4127444605
C82	2.2124423814	0.3328821892	-8.1456232605
C83	2.6611456058	0.2903524930	-9.4724137602
C84	3.0345387070	-0.9036736367	-10.0868755548
C85	2.9691390950	-2.1009600987	-9.3872029209
H86	2.7287845643	1.2029432055	-10.0507407589
H87	3.3815111434	-0.9206499848	-11.1141357342
C88	0.2410979113	3.9671870676	-3.4048435316
N89	0.2849633982	5.1499427665	-4.1504365547
C90	0.6750418768	5.2438920300	-5.4982284930
C91	-0.1104227416	6.3738678580	-3.4464089866
O92	0.6876078546	6.3200252339	-6.0806621040
O93	-0.1053394445	3.9900516912	-2.2301705770
C94	3.3745925138	-3.3623284878	-10.0521890312
N95	3.2952595359	-4.5375424640	-9.2916078167
C96	2.8585266020	-4.6104092348	-7.9607471518
C97	3.7087332287	-5.7730373334	-9.9648007081
O98	3.7667638493	-3.4063484248	-11.2108173668
O99	2.8177985481	-5.6770770299	-7.3612404459
H100	-0.0408284128	7.1988970301	-4.1508578697
H101	-1.1313128187	6.2706465632	-3.0726296767
H102	0.5504323302	6.5402934840	-2.5929744612
H103	3.5960501292	-6.5897159163	-9.2562593597
H104	3.0868689170	-5.9397418530	-10.8467958569
H105	4.7473987360	-5.6865566722	-10.2906736266
H106	0.1579933337	-0.0957200376	-1.4722493786
O107	-1.2839211423	-5.8220683618	0.2735656863
C108	-2.3026207952	-6.4648995882	-0.5115526792
O109	0.1932248527	-5.8291659345	-2.1449723171
C110	1.0679812265	-6.7765217467	-1.5076171191
H111	-3.1500087886	-5.7878103399	-0.6689387054
H112	-1.9121486703	-6.7938820201	-1.4779591466
H113	-2.6298058194	-7.3271389560	0.0719667542
H114	0.6450925066	-7.1317006273	-0.5635910986
H115	1.1709669324	-7.6076337928	-2.2068208897
H116	2.0509261432	-6.3282464394	-1.3254584339

Part G: DFT-Optimized Molecular Structure of PPPD-OPe

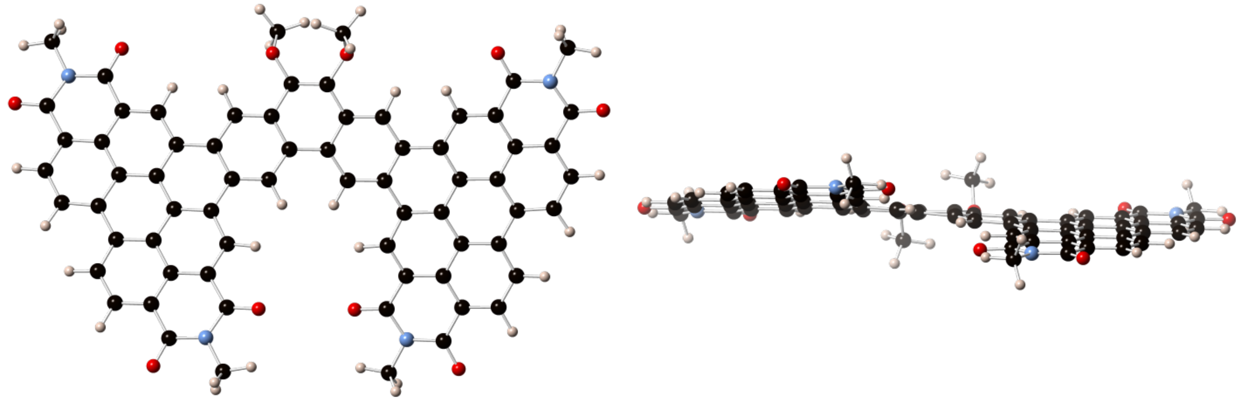

Figure S22. DFT-optimized model of **PPPD-OPe** (B3LYP/6-31G**) from different perspectives. Methyl groups substitute for the $CH(C_5H_{11})_2$ chains and C_5H_{11} chains to simplify the calculation.

Total energy = -3583.381789 hartrees

atom	x (angstroms)	y (angstroms)	z (angstroms)
C1	2.2598805513	1.3603290604	0.1812935206
C2	3.4122529676	0.5789790315	0.0945306698
C3	4.6720296636	1.2556581675	0.0586297546
C4	4.7077704088	2.6487375438	0.0380361469
C5	3.5452071677	3.4306326167	0.0873434689
C6	2.2844019426	2.7669877221	0.2016791977
C7	3.3958810283	-0.8866885057	0.0488671190
C8	4.6386700195	-1.5926674887	0.1052300813
C9	5.8888567960	-0.8643524407	0.1356623332
C10	5.9059378272	0.4980596755	0.0540292277
C11	3.5941916041	4.8846156191	0.0307814001
C12	2.4011968376	5.6380971944	0.1261047196
C13	1.1364305317	4.9735402866	0.2974148909
C14	1.0706665036	3.5596228206	0.3453175462
C15	4.8236278295	5.5750513947	-0.1363859946
C16	4.8830232919	6.9437786916	-0.2075156012
C17	3.6980530854	7.7225114547	-0.1092835337
C18	2.4445183278	7.0699651544	0.0619699433
C19	1.2596962131	7.8592531241	0.1660315770
C20	-0.0267141504	7.1842087052	0.3598647897
C21	-0.0569218004	5.7576254469	0.4253265271
C22	-1.3038376004	5.1013148573	0.6266292120
C23	-1.3417035271	3.6829908278	0.7068061160
C24	-0.1922205935	2.9481546224	0.5630426955
C25	-1.2334693086	7.8859686213	0.4891641585
C26	-2.4486093606	7.2314422836	0.6824863503
C27	-2.4932570419	5.8453811663	0.7550677640
C28	3.7725693164	9.1276752876	-0.1830133307

C29	2.6093657355	9.8811977430	-0.0865548263
C30	1.3767245162	9.2543167451	0.0854222252
C31	-3.7926907666	5.1631592842	0.9688730295
N32	-3.7710193296	3.7642015608	1.0525639126
C33	-2.6204180118	2.9666047990	0.9605554444
C34	6.2038133865	7.5982387520	-0.3925175700
N35	6.2123009218	8.9959236997	-0.4546067816
C36	5.0748442494	9.8167428876	-0.3628273931
O37	-2.6751880475	1.7506659235	1.0830396261
O38	-4.8506231627	5.7694273662	1.0758629612
O39	7.2507179814	6.9704471845	-0.4889338730
O40	5.1653897523	11.0352789237	-0.4295984493
C41	-5.0629792462	3.1077253354	1.2758604519
C42	7.5233924047	9.6284724129	-0.6333324946
C43	2.2279689162	-1.6410279930	-0.0586912522
C44	2.2210718958	-3.0475403730	-0.0833149626
C45	3.4640895520	-3.7409727474	0.0473868024
C46	4.6427239235	-2.9862243594	0.1204697082
C47	0.9913152356	-3.8098890729	-0.2464523483
C48	1.0234605432	-5.2252341099	-0.2013554952
C49	2.2703905593	-5.9200689329	-0.0171479624
C50	3.4793706827	-5.1959346621	0.0957594266
C51	-0.2530613900	-3.1659031936	-0.4788576933
C52	-1.4175703076	-3.8746475107	-0.6377249834
C53	-1.4145294789	-5.2942928665	-0.5591253972
C54	-0.1863546652	-5.9806654405	-0.3451241398
C55	-0.1910336557	-7.4075880211	-0.2822942966
C56	1.0773489630	-8.1130699250	-0.0795274344
C57	2.2792312309	-7.3529459787	0.0401473802
C58	3.5150432783	-8.0365564136	0.2188401064
C59	4.7172394654	-7.2869245250	0.3323277115
C60	4.6905451291	-5.9166503067	0.2703956984
C61	1.1612988381	-9.5106602677	-0.0070125975
C62	2.3762002134	-10.1685743855	0.1718904309
C63	3.5558259389	-9.4436473326	0.2835076808
C64	-2.6200274184	-6.0097942238	-0.7012863557
C65	-2.6098435972	-7.3966098303	-0.6287696042
C66	-1.4128757042	-8.0803326330	-0.4236676197
C67	-2.6765410311	-3.1289495607	-0.9057474842
N68	-3.8446963843	-3.8991035616	-1.0103823164
C69	-3.9004012039	-5.2970683821	-0.9282093072
C70	4.8395074150	-10.1653395804	0.4677023741
N71	5.9957938383	-9.3729696467	0.5712911992
C72	6.0208272085	-7.9746556305	0.5216283216
O73	-4.9712347731	-5.8780728614	-1.0467552957
O74	-2.7012736097	-1.9121709633	-1.0290843694
O75	7.0817425158	-7.3730501019	0.6306141447
O76	4.8997716788	-11.3862084556	0.5271830822
C77	7.2898559415	-10.0390886662	0.7520434642

C78	-5.1185902700	-3.2129100906	-1.2476083917
O79	7.0446986223	-1.6077013047	0.1725264146
O80	7.0785010605	1.2140929325	0.0404502641
C81	7.8991104229	1.0326987215	-1.1268287994
C82	7.8324466932	-1.4606462286	1.3669980930
H83	1.3048370253	0.8592102899	0.2435838444
H84	5.6842806638	3.1091257574	0.0011642720
H85	5.7607215362	5.0416516809	-0.2229655088
H86	-0.2935147992	1.8741432509	0.6417918710
H87	-1.2390657430	8.9673554179	0.4425830046
H88	-3.3746224133	7.7869912716	0.7818471760
H89	2.6844972635	10.9613227870	-0.1463593509
H90	0.4948239308	9.8776426498	0.1582550459
H91	-4.8919043039	2.0342007774	1.2797980548
H92	-5.4853320371	3.4313784308	2.2298329079
H93	-5.7579162000	3.3870001047	0.4817080054
H94	7.3716203605	10.7045180760	-0.6603931336
H95	8.1822060881	9.3528690829	0.1928721475
H96	7.9785096033	9.2803136566	-1.5630214682
H97	1.2857312957	-1.1181534373	-0.1346823955
H98	5.6077615425	-3.4686957445	0.1724616058
H99	-0.3288722708	-2.0891532342	-0.5552842879
H100	5.6392312214	-5.4063267729	0.3679713907
H101	0.2657414896	-10.1126304560	-0.0927883411
H102	2.4245507477	-11.2506294269	0.2246454874
H103	-3.5480328412	-7.9294782171	-0.7377234456
H104	-1.4448909417	-9.1612692095	-0.3775406992
H105	7.1122257956	-11.1115289519	0.7606783739
H106	7.9624635533	-9.7661461160	-0.0637432961
H107	7.7444389511	-9.7172317243	1.6914479147
H108	-4.9230971155	-2.1435772388	-1.2482973664
H109	-5.5373705979	-3.5259659487	-2.2066896265
H110	-5.8286880228	-3.4772602902	-0.4618094584
H111	8.7512806048	1.7022031055	-0.9998707756
H112	8.2440587352	-0.0011803340	-1.2120601619
H113	7.3469099146	1.3116482703	-2.0312993059
H114	8.1948574289	-0.4350641502	1.4784451938
H115	7.2477141323	-1.7419764556	2.2500432621
H116	8.6752550788	-2.1449420605	1.2564685202

Part H: Superimposition of the SCXRD Structures and DFT-Optimized Geometries

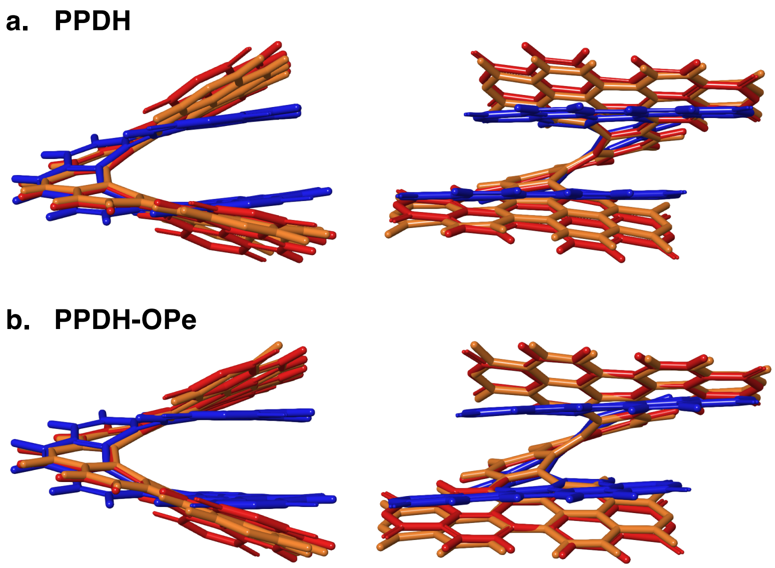

Figure S23. Optimization in the gas phase at the B3LYP/6-31G** level of theory returns geometries of **PPDH** and **PPDH-OPe** (depicted in red) that resemble the corresponding structures from SCXRD (orange). In contrast, optimization at the PW6B95-D3/6-31G** level of theory gives highly compressed bilayers (blue). We substituted methyl groups for the $CH(C_5H_{11})_2$ and C_5H_{11} chains to simplify the DFT calculations. These alkyl groups have been hidden in the structures above to provide an unobstructed view of the aryl surfaces.

VIII. Single-crystal X-ray Diffraction Data

Crystallographic data corresponding to **PPDH** have been deposited with the Cambridge Crystallographic Data Centre (CCDC #**1864290**).

PPDH·Anisole

**Formula**	$C_{113}H_{118}N_4O_9$
**MW**	1676.11
**Space group**	P-1
a **(Å)**	15.2191(5)
b **(Å)**	16.2207(6)
c **(Å)**	19.6692(8)
α **(°)**	75.736(3)
β **(°)**	74.285(3)
γ **(°)**	76.470(3)
**V ($Å^3$)**	4456.7(3)
**Z**	2
**ρ_{calc} (g cm^{-3})**	1.249
**T (K)**	100
**λ (Å)**	1.54184
**$2\theta_{min}$, $2\theta_{max}$**	7, 147
**Nref**	65476
**R(int), R(σ)**	.0667, .0662
**μ(mm^{-1})**	0.613
**Size (mm)**	.20 x .10 x .05
**T_{min} / T_{max}**	.795
**Data**	17715
**Restraints**	730
**Parameters**	1333
**R_1(obs)**	0.0947
**wR_2(all)**	0.3043
**S**	1.047
**Peak, hole (e^- $Å^{-3}$)**	0.83, -0.53

Crystallographic data corresponding to **PPDH-OPe** have been deposited with the Cambridge Crystallographic Data Centre (CCDC #**1864289**).

**PPDH-OPe·CF_3Ph**	
**Formula**	$C_{123}H_{135}F_3N_4O_{10}$
**MW**	1886.34
**Space group**	P-1
a **(Å)**	16.011(3)
b **(Å)**	17.232(3)
c **(Å)**	19.864(3)
α **(°)**	68.468(15)
β **(°)**	77.568(14)
γ **(°)**	82.152(13)
**V ($Å^3$)**	4968.3(15)
**Z**	2
**ρ_{calc} (g cm^{-3})**	1.261
**T (K)**	100
**λ (Å)**	1.54184
**$2\theta_{min}$, $2\theta_{max}$**	7, 78
**Nref**	13770
**R(int), R(σ)**	.0894, .1413
**μ(mm^{-1})**	0.659
**Size (mm)**	.16 x .03 x .02
**T_{min} / T_{max}**	.760
**Data**	5376
**Restraints**	101
**Parameters**	687
**R_1(obs)**	0.0960
**wR_2(all)**	0.2985
**S**	1.019
**Peak, hole (e^- $Å^{-3}$)**	0.46, -0.38

$R1 = [\Sigma(F_o - F_c)^2/\Sigma F_o^2]^{1/2}$; $wR2 = [\Sigma[w(F_o^2 - F_c^2)^2]/\Sigma w(F_o^2)^2]^{1/2}]$, $w = 1/[\sigma^2(F_o^2) + (aP)^2 + bP]$, where $P = [\max(F_o^2,0) + 2(F_c^2)]/3$

IX. References

(1) Rajasingh, P.; Cohen, R.; Shirman, E.; Shimon, L. J. W.; Rybtchinski, B. Selective Bromination of Perylene Diimides under Mild Conditions. *J. Org. Chem.* **2007**, *72*, 5973–5979.

(2) Scott, D. W.; Bunce, R. A.; Materer, N. F. Synthesis of 3,6-Dihalophenanthrene Derivatives. *Org. Prep. Proced. Int.* **2006**, *38*, 325–331.

(3) Francke, R.; Little, R. D. Optimizing Electron Transfer Mediators Based on Arylimidazoles by Ring Fusion: Synthesis, Electrochemistry, and Computational Analysis of 2-Aryl-1-Methylphenanthro[9,10-d]Imidazoles. *J. Am. Chem. Soc.* **2014**, *136*, 427–435.

(4) Blanc, E.; Schwarzenbach, D.; Flack, H. D. The Evaluation of Transmission Factors and Their First Derivatives with Respect to Crystal Shape Parameters. *J. Appl. Crystallogr.* **1991**, *24*, 1035–1041.

(5) Clark, R. C.; Reid, J. S. The Analytical Calculation of Absorption in Multifaceted Crystals. *Acta Crystallogr. Sect. A* **1995**, *51*, 887–897.

(6) Sheldrick, G. M. SHELXT – Integrated Space-Group and Crystal-Structure Determination. *Acta Crystallogr. Sect. A* **2015**, *71*, 3–8.

(7) Sheldrick, G. M. Crystal Structure Refinement with SHELXL. *Acta Crystallogr. Sect. C* **2015**, *71*, 3–8.

(8) Dolomanov, O. V.; Bourhis, L. J.; Gildea, R. J.; Howard, J. A. K.; Puschmann, H. OLEX2 : A Complete Structure Solution, Refinement and Analysis Program. *J. Appl. Crystallogr.* **2009**, *42*, 339–341.

(9) Bochevarov, A. D.; Harder, E.; Hughes, T. F.; Greenwood, J. R.; Braden, D. A.; Philipp, D. M.; Rinaldo, D.; Halls, M. D.; Zhang, J.; Friesner, R. A. Jaguar: A High-Performance Quantum Chemistry Software Program with Strengths in Life and Materials Sciences. *Int. J. Quantum Chem.* **2013**, *113*, 2110–2142.

(10) Goerigk, L.; Grimme, S. A thorough benchmark of density functional methods for general main group thermochemistry, kinetics, and noncovalent interactions. *Phys. Chem. Chem. Phys.* **2011**, *13*, 6670-6688.
